# Parallel Synthesis of Piperazine Tethered Thiazole Compounds with Antiplasmodial Activity

**DOI:** 10.3390/ijms242417414

**Published:** 2023-12-12

**Authors:** Ramanjaneyulu Rayala, Prakash Chaudhari, Ashley Bunnell, Bracken Roberts, Debopam Chakrabarti, Adel Nefzi

**Affiliations:** 1 Herbert Wertheim College of Medicine, Center for Translational Science, Florida International University, Miami, FL 33199, USA; rraya002@fiu.edu (R.R.); pchaudha@fiu.edu (P.C.); abunnell@fiu.edu (A.B.); 2 Division of Molecular Microbiology, Burnett School of Biomedical Sciences, University of Central Florida, Orlando, FL 32826, USA; broberts@ucf.edu (B.R.); debopam.chakrabarti@ucf.edu (D.C.)

**Keywords:** heterocyclic compounds, thiazole tethered piperazine, combinatorial chemistry, solid-phase synthesis, malaria, antiplasmodial activity

## Abstract

Thiazole and piperazine are two important heterocyclic rings that play a prominent role in nature and have a broad range of applications in agricultural and medicinal chemistry. Herein, we report the parallel synthesis of a library of diverse piperazine-tethered thiazole compounds. The reaction of piperazine with newly generated 4-chloromethyl-2-amino thiazoles led to the desired piperazine thiazole compounds with high purities and good overall yields. Using a variety of commercially available carboxylic acids, the parallel synthesis of a variety of disubstituted 4-(piperazin-1-ylmethyl)thiazol-2-amine derivatives is described. the screening of the compounds led to the identification of antiplasmodial compounds that exhibited interesting antimalarial activity, primarily against the *Plasmodium falciparum* chloroquine-resistant Dd2 strain. The hit compound **2291-61** demonstrated an antiplasmodial EC_50_ of 102 nM in the chloroquine-resistant Dd2 strain and a selectivity of over 140.

## 1. Introduction

Combinatorial chemistry is a highly efficient approach for the parallel synthesis of novel small-molecule heterocyclic compounds [1,2,3,4,5]. Due to the well-established protocols set forth for solid-phase synthesis [6,7,8,9], large collections of organic compounds can readily be made available for the purpose of drug discovery [6,10,11,12,13,14]. The focused efforts over the past twenty years for the target-oriented synthesis and diversity-oriented synthesis of low-molecular-weight acyclic and heterocyclic combinatorial libraries for therapeutic applications have been highly fruitful [15]. However, the need for more synthetic pathways for solid-phase synthesis, especially those yielding valuable drugs like heterocyclic-containing compounds such as thiazoles and piperazines, is never satisfied [16,17,18,19,20,21].

Piperazine and thiazole rings are found in a large number of pharmacologically active molecules [16,17,20,21,22,23,24,25,26,27]. Small molecules containing the thiazole moiety have been demonstrated to possess drug-like properties against a variety of diseases, resulting in, so far, 17 FDA-approved drugs containing the thiazole ring. The indications to which thiazole derivatives are prescribed include asthma (Cinalukast) [28], bacterial infections (Ceftizoxime) [29], diarrhea (Nitazoxanide) [30], myelogenous leukemia (Dasatinib) [31], pain (Meloxicam) [32], duodenal ulcers (famotidine) [33], anthelmintics (thiabendazole) [34], CNS neuroprotective and anticonvulsant (riluzole) [35], and vitamin (thiamine) [36].

Furthermore, the piperazine nucleus is one of the most important heterocycles, exhibiting remarkable pharmacological activities [23,24,37,38,39]. Piperazine scaffolds are among the most extensively used backbones in medicinal chemistry, and many bioactive compounds are built upon this template [23,39,40]. The physicochemical properties and the three-dimensional structures of the different piperazine chemotypes are of utmost importance to understanding their biological activities. Small molecules containing the piperazine moiety have been found in different therapeutics, resulting in 39 FDA-approved drugs containing the piperazine ring.

## 2. Research

**Figure 1:**
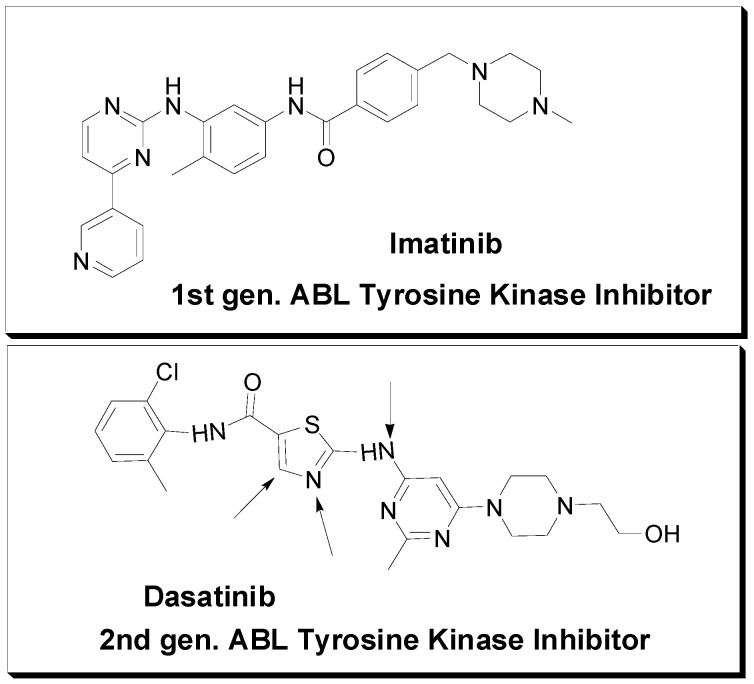
The 1st and 2nd generation ABL Tyrosine Kinase Inhibitors. The arrows indicate the key points of hydrogen and heteroatom interactions with ABL Tyrosine Kinase.

Continuing with our work toward the synthesis of drug-like compounds, we developed different strategies toward the synthesis of small-molecule compounds comprising the piperazine and the thiazole pharmacophores. A search of the FDA-approved drugs resulted in only 1 compound that contains both a thiazole and a piperazine group–Dasatinib [31,41] (Figure 1), which is marketed by the name Sprycel^®^ by Brystol-Meyers Squibb. Dasatinib is a 2nd-generation ABL Tyrosine kinase inhibitor (TKI) used for the treatment of chronic myelogenous leukemia and Philadelphia-chromosome-positive acute lymphoblastic leukemia. In contrast to its 1st generation TKI predecessor (Imatinib–Figure 1), it appears to have overcome the treatment resistance problems observed.

**Figure 2:**
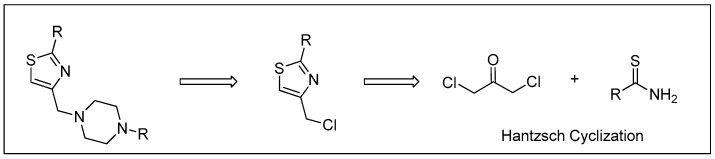
Retrosynthesis of piperazine-thiazoles via Hantzsch cyclization and nucleophilic displacement.

Approximately 2-Aminothiazoles are readily obtained by Hantzsch’s cyclocondensation of thiourea with α-haloketones or by the reaction of α-thiocyanate carbonyl compounds with aromatic or aliphatic amine hydrochlorides [42,43,44]. They can also be obtained following a one-pot reaction of ketones with a mixture of N-bromosuccinimide, thiourea, and benzoyl peroxide. We previously reported the use of 1,3-dichloroacetone to generate a methylchloride at C^4^ of the thiazole ring, which can be used as a center of diversity to incorporate different nucleophiles [20,45,46]. We also used the newly generated 4-chloromethyl thiazole as a cyclative approach for the solid-phase synthesis of thiazole-containing cyclic peptides [43]. In this paper, we describe the parallel synthesis of diverse thiazole-tethered piperazine libraries (**2291**) (Figure 2) following the displacement of the chloro group with piperazine.

**Scheme 1:**
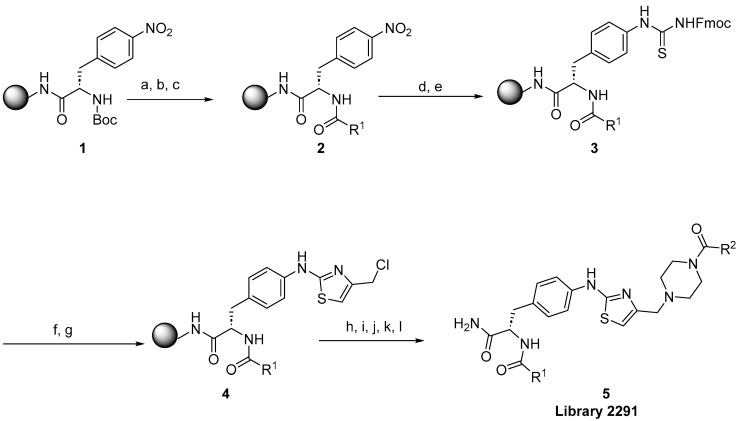
(**a**) TFA (55% in DCM); (**b**) 5% DIPEA in DCM; (**c**) R^1^COOH (10 eq.), DIC (10 eq.), DMF, R.T.; overnight; (**d**) SnCl_2-_2H_2_O (1M) in DMF, R.T.; overnight; (**e**) Fmoc-NCS, DMF, R.T.; (**f**) Piperidine (20% in DMF); (**g**) 1,3-dichloroacetone, DMF, 85 °C, overnight; (**h**) Boc-piperazine, DIPEA in DMF; (**i**) TFA (55% in DCM); (**j**) 5% DIPEA in DCM; (**k**) R^2^COOH, DIC, DMF, R.T.; overnight; (**l**) HF (95:5), 0 °C, 90 min.

Starting from p-methylbenzhydrylamine hydrochloride resin (MBHA·HCl)-bound Fmoc-L-4-nitrophenylalanine **1** (Scheme 1), a library of arylaminothiazole piperazine compounds was prepared. Thus, following Boc deprotection and neutralization of the amine, the free amine was functionalized with different commercially available carboxylic acids (diversity R^1^) (Scheme 1). The nitro group was reduced in the presence of tin chloride to generate the corresponding aniline derivative, which was then treated with Fmoc-protected isothiocyanate (Fmoc-NCS) to afford the protected thiourea **3**. Upon Fmoc deprotection [47], the thioureas were cyclized with 1,3-dichloroacetone via a Hantzsch cyclization [48] to afford the corresponding resin-bound 4-chloromethyl-thiazole **4** in a clean reaction confirmed by LC-MS. Boc-piperazine was then introduced via nucleophilic displacement of the chloro group. Following Boc deprotection and acylation of the piperazine using four different carboxylic acids (diversity R^2^) (Scheme 1), a library of 220 different arylaminothiazole-tethered piperazine compounds **5** was prepared in good purity (UV traces were monitored at 214 and 254 nm) and high yields (Table 1).

Continuing with our efforts to screen our small-molecule libraries against a variety of infectious diseases [45,49,50,51], all the compounds were screened against the *P. falciparum* (Pf) Dd2 strain. Malaria is a life-threatening disease that still afflicts about 300 million individuals worldwide, and half of the global population is at risk [52,53,54,55,56,57,58]. Unfortunately, most of the drugs that are currently being used for malaria treatment were developed more than 30 years ago, and many are derivatives of older drugs [54,55,58,59,60,61,62].

**Figure 3:**
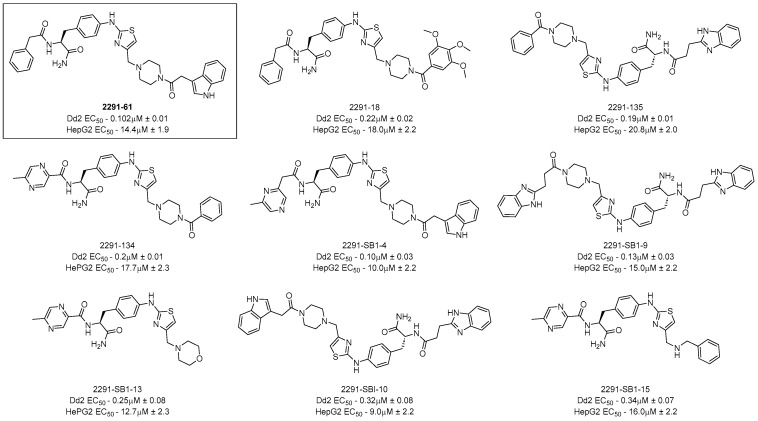
Structures and activity of identified antiplasmodial hits.

To test if the thiazole-tethered piperazine compounds are active against drug-resistant parasites, we used the multi-drug-resistant Pf Dd2 strain for the assay. The growth inhibition was determined by the SYBR green I fluorescence assay using standard culture conditions for 72 h as described in the experimental section [63,64,65]. Compounds with EC_50_ values ranging between 100 and 350 nM were identified (Figure 3). As a counterscreen, we evaluated the cytotoxicity of these compounds in the human hepatocyte cell line HepG2 using the MTS cell proliferation assay. The compounds exhibited a very promising selectivity of >60.

**Figure 4:**
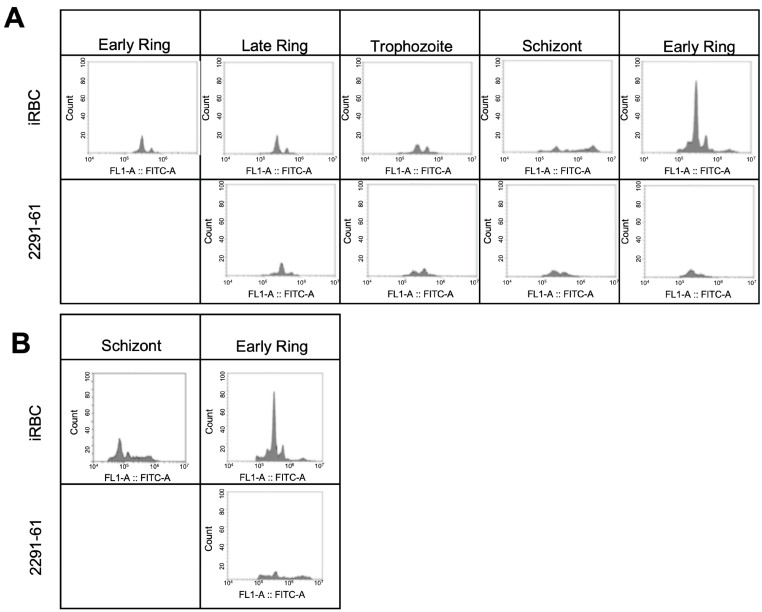
Stage-Specific Activity of TPIMS **2291-61**.

To test if our hit compound, **2291-61**, has a cellular mechanism of action distinct from known antimalarials, we determined the effect of **2291-61** on intraerythrocytic developmental stages. We analyzed the effect of the compound on tightly synchronized malaria parasite cultures. We noticed that when **2291-61** is added at early ring or late ring stages, it prevents development into the trophozoite stage; however, if given at trophozoite or schizont stages, it prevents schizont development (Figure 4). This demonstrates that **2291-61** is an early-acting compound that can inhibit multiple stages of development.

Tightly synchronized cultures were treated with **2291-61** at 6 (early ring), 18 (late ring), 30 (trophozoite), and 42 (schizont) hours post-invasion. Treated cultures were monitored for effect on parasite development at 12 h intervals. Inhibition of cellular growth was assessed in terms of reduction of DNA content as determined by flow cytometric analysis of YOYO-1-stained samples. As shown in Figure 4A, early ring treatment (6 h post-invasion) shows rapid activity of 2291 in blocking ring development. The treatment of **2291-61** developing trophozoites and schizonts results in blocked progression beyond schizont development (Figure 4B).

In conclusion, we have developed an efficient approach for the parallel synthesis of pharmacologically relevant bis-heterocycles containing piperazine and thiazole pharmacophores in the core. Using the strategy outlined in Scheme 1 and a large number of commercially available carboxylic acids, we prepared piperazine-tethered thiazole libraries as part of our drug discovery program. The screening of the piperazine-tethered thiazole compounds led to the identification of antiplasmodial compounds that exhibited interesting antimalarial activity, primarily against the chloroquine-resistant Dd2 strain. Hit compound **2291-61** demonstrated an antiplasmodial EC_50_ of 102 nM in the chloroquine-resistant Dd2 strain and a selectivity of over 140.

## 3. Experimental Procedures

### 3.1. Materials and General Methods

All reagents and solvents were purchased from various commercial sources (Sigma Aldrich (St. Louis, MO, USA), Chemimpex (Wood Dale, IL, USA), VWR (West Chester, PA, USA), Cambridge Isotopes (Tewksbury, MA, USA), etc.) and used without further purification unless otherwise stated. Yields were based on the manufacturer’s reported loading of MBHA resin of 1.15 mmol per gram of resin. ^1^H NMR spectra were recorded at 500 MHz and 400 MHz, and ^13^C NMR spectra were recorded at 125 MHz and 100 MHz in deuterated chloroform, and all chemical shifts are reported in δ units relative to TMS. All L-amino acids used were assumed to be enantiomerically pure from the supplier. LC-MS was performed on crude samples dissolved in 50:50 (acetonitrile and water) at a concentration of 1 mg/mL on a Shimadzu LC-MS equipped with a Vydac column with a gradient of 5–95% formic acid in acetonitrile over 7 min with their UV traces monitored at λ = 214 and 254 nm. HPLC purification was performed on a Phenomenex Luna 150 × 21.2 mm 5 micron column with a flow rate of 12 mL/min.

All compounds were synthesized following the strategy outlined in Scheme 1. The parallel solid phase synthesis was performed using the “tea-bag” methodology [66].

General Synthesis of the disubstituted arylaminothiazole-tethered piperazine compounds **5**:

A 100 mg sample of p-methylbenzhydrylamine hydrochloride (MBHA·HCl) resin with a molar loading of 1.15 mmole/g and size of 100–200 mesh was thermally sealed into a polypropylene mesh bag per compound. All bags were made and were washed in triplicate with DCM, 5% diisopropylethylamine (DIEA) in DCM, and finally DCM to neutralize the acidic resin. The neutralized resin bags were then treated with 3 equivalents of Boc-Phe(4-NO_2_)-OH (3.2 g), HOBt (1.6 g), and DIC (2.1 mL) in 75 mL of DMF in a 150 mL polystyrene bottle. The bottle was placed on a mechanical shaker and left for approximately 3 h. A ninhydrin test [67] was used on a few grains of the resin to confirm the complete acylation of the resin amine. The Boc protecting group was removed from the N-terminus by shaking the tea bags in a 55% solution of TFA in DCM for 30 min. Upon completion of the TFA treatment, the bags were thoroughly washed with DCM (×3), neutralized with 5% DIEA in DCM (×4), and washed with DCM (3 times). The N-terminus was acylated with different carboxylic acids (10 equivalents of the acid) in the presence of 10 equivalents of DIC in 25 mL of THF. The reactions were allowed to shake overnight (~15 h) before they were thoroughly washed with DMF, followed by DCM. A ninhydrin test of a few grains of resin confirmed that all the acylation reactions were complete. All bags were combined into a 150 mL polystyrene bottle. Stannous chloride (20 eq, 13.1 g) was added to the bottle along with 69 mL of DMF to afford a concentration of 1.0 M. The bottle was gently sonicated for approximately 20 min to ensure a homogenous solution and then set to shake overnight (~15 h). In the morning, the bags were washed 12 times with DMF, 6 times with MeOH, and 6 times with DCM to ensure complete removal of the tin. A few milligrams of resin were then removed from bag #5 and used as a control to confirm complete reduction by cleavage with HF for 1.5 h. LC-MS of the resulting product confirmed the reduction was complete. All bags were combined in a 150 mL polystyrene bottle. Fmoc-NCS (2.5 equivalents, 2.43 g) was weighed out and added to the polystyrene bottle. The bottle was purged with nitrogen gas for approximately 10 min before anhydrous DMF (~60 mL) was added via canula. The bottle was covered and sealed with parafilm and then sonicated for ~10 min to ensure a homogenous solution. The bottle was shaken on a mechanical shaker overnight. In the morning, the bags were thoroughly washed with DMF and DCM. All bags were combined in a 150 mL polystyrene bottle, and the Fmoc group was deprotected using a 20% solution of piperidine in DMF for 10 min at room temperature. The bags were then thoroughly washed with DMF and DCM to ensure complete removal of the piperidine. The bags were then treated with 10 equivalents of 1,3-dichloroacetone (4.38 g) in 115 mL of anhydrous DMF at 85 °C overnight to undergo Hantzch’s Cyclization. The bags were thoroughly washed with DMF and DCM, and the corresponding chloromethyl thiazole was treated with Boc-piperazine (6 equivalents, 3.86 g) in 100 mL of DMF at room temperature overnight. The Boc protecting group was removed from the piperazine in the presence of a 55% solution of trifluoroacetic acid in dichloromethane for 30 min. The bags were thoroughly washed with DCM (3 times), neutralized with 5% DIEA (×4) in dichloromethane, and washed with DCM (3 times). The generated piperazine was acylated using different carboxylic acids. The reactions were allowed to shake overnight before they were thoroughly washed with DMF, followed by DCM. The dried bags were treated with HF/anisole (95/5) at 0 °C for 1.5 h before the HF was blown off with nitrogen. Following extraction and lyophilization, the crude compounds were purified by RP-HPLC. All compounds were obtained in good yields.

**2-(2-phenylacetamido)-3-(4-((4-((4-(3,4,5-trimethoxybenzoyl)piperazin-1-yl)methyl)thiazol-2-yl)amino)phenyl)propanamide (2291-18)** ^1^H NMR (400 MHz, CDCl_3_, 300 K) 10.29 (s, 1H), 8.24 (d, *J* = 8 Hz, 2H), 7.51–7.49 (m, 3H), 7.19–7.14 (m, 5H), 6.44 (s, 1H), 7.08–7.06 (m, 4H), 6.73 (s, 2H), 4.45–4.39 (m, 3H), 4.32 (s, 4H), 3.80 (s, 6H), 3.69 (s, 3H), 3.44 (m, 4H), 3.22 (s, 2H), 3.00–2.95 (dd, *J* = 14, 4 Hz, 1H), 2.74–2.68 (m, 1H), 1.19 (s, 1H); 13C NMR (100 MHz, CDCl_3_, 300 K) 173.6, 170.3, 169.4, 164.9, 153.3 (2C), 141.1, 139.6, 139.2, 136.8, 131.7 (2CH), 130.4, 130.2 (2CH), 129.4 (2CH), 128.5 (2CH), 126.6 (2CH), 117.4 (2CH), 111.9, 105.2 (2C), 60.5 (2CH3), 56.5 (2CH2), 54.4, 51.2, 42.6 (2CH_2_), 37.6, 21.50. **LCMS:** C_35_H_40_N_6_O_6_S (M calculated) 672.27, C_35_H_38_FN_6_O_3_S (MH^+^ found) 673.

**5a** (**^1^H NMR, 400 MHz, CDCl_3_, 300 K)** 7.30–7.27 (m, 2H), 7.21–7.11 (m, 7H), 7.07–6.98 (m, 4H), 6.44 (s, 1H), 6.16 (d, *J* = 10.0 Hz, 1H), 5.94 (s_(broad)_, 1H), 5.61 (s_(broad)_, 1H), 4.64 (q, *J* = 9.3 Hz, 1H), 3.76 (s_(broad)_, 2H), 3.51 (s, 2H), 2.17 (s, 2H), 2.98 (d, *J* = 8.6 Hz, 2H), 2.54 (s_(broad)_, 2H), 2.24 (s_(broad)_, 2H), 1.42 (m, 2H), 1.18 (m, 2H)**. (^13^C NMR, 100 MHz, CDCl_3_, 300 K)** 172.6, 171.0, 170.8, 164.7, 140.6, 139.2, 130.9, 130.8, 130.7, 130.2, 128.7, 126.4, 125.3, 118.2, 116.0, 115.8, 77.2, 58.8, 53.9, 52.5, 42.6, 37.2, 29.3, 15.1. **LCMS:** C_35_H_37_FN_6_O_3_S (M calculated) 640.77, C_35_H_38_FN_6_O_3_S (MH^+^ found) 641.

**5b (^1^H NMR, 400 MHz, CDCl_3_, 300 K)** 7.18 (d, *J* = 13.3 Hz, 2H), 7.14 (t, *J* = 3.7 Hz, 2H), 7.06 (d, *J* = 8.8 Hz, 2H), 7.01 (t, *J* = 7.4 Hz, 2H), 6.64 (s, 2H), 6.55 (s_(broad)_, 1H), 6.22 (d, *J* = 8.1 Hz, 1H), 6.03 (s_(broad)_, 1H), 5.66 (s_(broad)_, 1H), 4.66 (q, *J* = 9.3 Hz, 1H), 3.87 (s, 12H), 3.63 (s, 2H), 3.53 (s, 2H), 3.00 (d *J* = 8.1 Hz, 2H), 2.65 (s_(broad)_, 4H). **(^13^C NMR, 100 MHz, CDCl_3_, 300 K)** 172.7, 170.8, 170.1, 164.7, 160.9, 158.8, 153.4, 139.1, 130.9, 130.8, 130.3, 118.2, 116.0, 115.7, 104.6, 77.2, 60.9, 56.3, 53.9, 42.6, 37.2. **LCMS:** C_35_H_39_FN_6_O_6_S (M calculated) 690.78, C_35_H_40_FN_6_O_6_S (MH^+^ found) 691.

**5c (^1^H NMR, 400 MHz, CDCl_3_, 300 K)** 7.20 (d, *J* = 7.4 Hz, 2H), 7.15 (t, *J* = 8.2 Hz, 2H), 7.05 (d, *J* = 8.2 Hz, 2H), 7.00 (t, *J* = 8.9 Hz, 2H), 6.50 (s, 1H), 6.25 (d, *J* = 8.2 Hz, 1H), 6.03 (s_(broad)_, 1H), 5.73 (s_(broad)_, 1H), 4.66 (q, *J* = 7.4 Hz, 1H), 3.71–3.68 (m, 2H), 3.67–3.58 (m, 2H), 3.56 (d, *J* = 7.0 Hz, 2H), 3.53 (s, 3H), 3.00 (d, *J* = 7.8 Hz, 2H), 2.89 (pent. *J* = 7.0 Hz, 1H), 2.58–2.52 (m, 5H), 1.88–1.64 (m, 8H), 1.62–1.51 (m, 3H). **(^13^C NMR, 100 MHz, CDCl_3_, 300 K)** 174.5, 172.7, 170.8, 164.7, 163.4, 139.2, 130.9, 130.8, 130.6, 130.2, 118.2, 115.9, 115.7, 77.2, 58.2, 53.9, 42.6, 41.0, 37.2, 30.1, 26.0. **LCMS:** C_31_H_37_FN_6_O_3_S (M calculated) 592.73, C_31_H_38_FN_6_O_3_S (MH^+^ found) 593.

**5d (^1^H NMR, 400 MHz, CDCl_3_, 300 K)** 7.20 (d, *J* = 8.2 Hz, 2H), 7.15 (t, *J* = 6.7 Hz, 2H), 7.06 (d, *J* = 8.2 Hz, 2H), 7.01 (t, *J* = 7.5 Hz, 2H), 6.50 (s, 1H), 6.25 (d, *J* = 8.2 Hz, 1H), 6.02 (s_(broad)_, 1H), 5.71 (s_(broad)_, 1H), 4.67 (q, *J* = 8.2 Hz, 1H), 3.71–3.68 (m, 2H), 3.67–3.58 (m, 4H), 3.53 (s, 3H), 3.00 (d, *J* = 9.8 Hz, 2H), 2.60–2.51 (m, 5H), 1.86–1.73 (m, 4H), 1.54–1.50 (m, 4H), 0.98 (d, *J* = 8.9 Hz, 2H), 0.90 (d, *J* = 7.2 Hz, 2H). **(^13^C NMR, 100 MHz, CDCl_3_, 300 K)** 174.6, 172.7, 170.8, 164.7, 139.2, 130.9, 130.8, 130.7, 130.2, 118.2, 116.0, 115.7, 105.7, 77.2, 53.9, 42.6, 40.1, 38.8, 37.3, 34.5, 32.1, 31.1, 29.3, 28.4, 24.9, 22.6, 18.9. **LCMS:** C_33_H_41_FN_6_O_3_S (M calculated) 620.78, C_33_H_42_FN_6_O_3_S (MH^+^ found) 621.

**5e (^1^H NMR, 400 MHz, CDCl_3_, 300 K)** 7.30 (t, *J* = 6.9 Hz, 1H), 7.21 (d, *J* = 8.4 Hz, 1H), 7.16 (d, *J* = 8.4 Hz, 4H), 7.08 (d, *J* = 6.8 Hz, 2H), 7.05 (d, *J* = 8.4 Hz, 2H), 6.86 (d, *J* = 8.4 Hz, 2H), 6.45 (s, 1H), 6.14 (d, *J* = 9.9 Hz, 1H), 6.10 (s_(broad)_, 1H), 5.65 (s_(broad)_, 1H), 4.66 (q, *J* = 7.6 Hz, 1H), 3.80 (s, 3H), 3.74 (s_(broad)_, 2H), 3.55 (s_(broad)_, 2H), 3.50 (s, 3H), 2.98 (d, *J* = 6.5 Hz, 2H), 2.55 (s_(broad),_ 2H), 2.26 (s_(broad)_, 2H), 1.44–1.42 (m, 2H), 1.21–1.18 (m, 2H). **(^13^C NMR, 100 MHz, CDCl_3_, 300 K)** 172.8, 171.6, 171.0, 164.9, 159.0, 140.6, 139.1, 130.9, 130.4, 130.3, 128.7, 126.4, 126.2, 125.3, 118.3, 114.5, 77.2, 57.9, 55.3, 53.9, 52.4, 42.7, 37.0, 29.3, 15.1. **LCMS:** C_36_H_40_N_6_O_4_S (M calculated) 652.81, C_36_H_41_N_6_O_4_S (MH^+^ found) 653.

**5f (^1^H NMR, 400 MHz, CDCl_3_, 300 K)** 7.19 (d, *J* = 9.1 Hz, 2H), 7.08 (d, *J* = 6.1 Hz, 2H), 7.06 (d, *J* = 4.6 Hz, 2H), 6.87 (d, *J* = 7.6 Hz, 2H), 6.64 (s, 2H), 6.55 (s_(broad)_, 1H), 6.10 (d, *J* = 5.4 Hz, 1H), 6.07 (s_(broad)_, 1H), 5.58 (s_(broad)_, 1H), 4.66 (q, *J* = 8.4 Hz, 1H), 3.87 (s, 3H), 3.80 (s, 4H), 3.64 (s_(broad)_, 2H), 3.50 (s_(broad)_, 2H), 3.01–2.96 (m, 2H), 2.66 (s_(broad)_, 6H). **(^13^C NMR, 100 MHz, CDCl_3_, 300 K)** 172.8, 171.6, 170.1, 165.2, 159.0, 153.4, 139.0, 131.1, 130.4, 130.3, 126.2, 118.4, 114.5, 104.6, 77.2, 60.9, 56.3, 55.3, 42.7, 37.0. **LCMS:** C_36_H_42_N_6_O_7_S (M calculated) 702.82, C_36_H_43_N_6_O_7_S (MH^+^ found) 703.

**5g (^1^H NMR, 400 MHz, CDCl_3_, 300 K)** 7.19 (d, *J* = 9.1 Hz, 2H), 7.08 (d, *J* = 6.1 Hz, 2H), 7.06 (d, *J* = 4.6 Hz, 2H), 6.52 (s_(broad)_, 1H), 6.15 (d, *J* = 5.4 Hz, 1H), 6.10 (s_(broad)_, 1H), 5.66 (s_(broad)_, 1H), 4.67 (q, *J* = 8.4 Hz, 1H), 3.80 (s, 3H), 3.73 (s_(broad)_, 2H), 3.66–3.56 (m, 4H), 3.51 (s, 2H), 2.99 (d, *J* = 6.9 Hz, 2H), 2.88 (pent. *J* = 7.7 Hz, 1H), 2.64–2.52 (m, 4H), 1.89–1.69 (m, 7H), 1.64–1.52 (m, 3H)**. (^13^C NMR, 100 MHz, CDCl_3_, 300 K)** 174.5, 172.8, 171.6, 164.9, 159.0, 139.1, 130.9, 130.4, 130.3, 126.2, 118.3, 114.5, 77.2, 58.0, 55.3, 53.9, 52.7, 45.1, 42.7, 41.0, 37.0, 30.1, 26.0. **LCMS:** C_32_H_40_N_6_O_4_S (M calculated) 604.76, C_32_H_41_N_6_O_4_S (MH^+^ found) 605.

**5h (^1^H NMR, 400 MHz, CDCl_3_, 300 K)** 7.18 (d, *J* = 9.1 Hz, 2H), 7.09 (d, *J* = 6.1 Hz, 2H), 7.06 (d, *J* = 4.6 Hz, 2H), 6.87 (d, *J* = 9.0 Hz, 2H), 6.46 (s, 1H), 6.12 (d, *J* = 5.4 Hz, 1H), 6.06 (s_(broad)_, 1H), 5.64 (s_(broad)_, 1H), 4.66 (q, *J* = 8.4 Hz, 1H), 3.81 (s, 3H), 3.67 (s_(broad)_, 2H), 3.59–3.51 (m, 4H), 3.51 (s, 2H), 2.98 (d, *J* = 6.9 Hz, 2H), 2.660–2.46 (m, 5H), 1.85–1.70 (m, 4H), 1.57–1.46 (m, 4H), 0.99 (d, *J* = 5.4 Hz, 2H), 0.90 (d, *J* = 5.7 Hz, 2H)**. (^13^C NMR, 100 MHz, CDCl_3_, 300 K)** 174.7, 172.8, 171.6, 164.9, 159.0, 139.1, 130.8, 130.4, 130.3, 126.2, 118.3, 114.5, 105.2, 77.2, 58.2, 55.3, 53.9, 42.7, 40.2, 38.8, 37.0, 34.6, 32.1, 29.3, 28.4, 24.9, 22.6, 18.9. **LCMS:** C_34_H_44_N_6_O_4_S (M calculated) 632.82, C_34_H_45_N_6_O_4_S (MH^+^ found) 633.

**5i (^1^H NMR, 400 MHz, CDCl_3_, 300 K)** 9.24 (s, 1H), 8.41 (s, 1H), 8.30 (d, *J* = 12.1 Hz, 1H), 8.17 (s, 1H), 7.31–7.27 (m, 4H), 7.24 (d, *J* = 7.1 Hz, 2H), 7.20 (t, *J* = 7.1 Hz, 1H), 7.16 (d, *J* = 10.9 Hz, 2H), 6.46 (s, 1H), 6.02 (s_(broad)_, 1H), 5.69 (s_(broad)_, 1H), 4.88 (q, *J* = 8.4 Hz, 1H), 3.76 (s_(broad)_, 2H), 3.57 (s_(broad),_ 2H), 3.52 (s, 2H), 3.22 (d, *J* = 9.1 Hz, 2H), 2.67 (s, 3H), 2.59 (s_(broad)_, 2H), 2.30 (s_(broad)_, 2H), 1.44–1.40 (m, 2H), 1.22–1.16 (m, 2H)**. (^13^C NMR, 100 MHz, CDCl_3_, 300 K)** 172.5, 172.2, 171.0, 165.5, 163.4, 157.6, 150.2, 143.3, 142.6, 141.1, 140.5, 139.0, 131.3, 130.4, 128.8, 126.4, 125.3, 118.5, 57.2, 54.2, 52.2, 37.5, 29.3, 21.8, 15.1. **LCMS:** C_33_H_36_N_8_O_3_S (M calculated) 624.76, C_33_H_37_N_8_O_3_S (MH^+^ found) 625.

**5j (^1^H NMR, 400 MHz, CDCl_3_, 300 K)** 9.15 (s, 1H), 8.32 (s, 1H), 8.22 (d, *J* = 8.6 Hz, 1H), 7.17 (d, *J* = 5.9 Hz, 4H), 6.55 (s, 2H), 6.38 (s_broad_, 1H), 5.90 (s_broad_, 1H), 5.53 (s_broad_, 1H), 5.23 (s, 2H), 4.77 (q, *J* = 7.6 Hz, 1H), 3.79 (s, 9H), 3.78 (s, 2H), 3.46 (s, 2H), 3.12 (d, *J* = 6.5 HZ, 2H), 2.58 (s, 3H), 2.48 (s_v. broad_, 4H), 1.80 (s_v.broad_, 4H)**. (^13^C NMR, 100 MHz, CDCl_3_, 300 K)** 172.4, 170.1, 164.8, 163.4, 157.6, 153.3, 143.3, 142.6, 141.2, 139.4, 139.2, 131.1, 130.4, 118.3, 104.6, 60.9, 58.2, 56.3, 54.2, 53.4, 37.5, 21.8. **LCMS:** C_33_H_38_N_8_O_6_S (M calculated) 674.77, C_33_H_39_N_8_O_6_S (MH^+^ found) 675.

**5k (^1^H NMR, 400 MHz, CDCl_3_, 300 K)** 9.24 (s, 1H), 8.42 (s, 1H), 8.32 (d, *J* = 8.3 Hz, 1H), 7.26 (d, *J* = 6.0 Hz, 4H), 6.47 (s, 1H), 5.97 (s_broad_, 1H), 5.64 (s_broad_, 1H), 5. 32 (s, 2H), 4.87 (q, *J* = 6.8 Hz, 1H), 3.73–3.66 (m, 2H), 3.62–3.57 (m, 2H), 3.53 (s, 2H), 3.24–3.19 (m, 2H), 2.89 (pent., *J* = 7.6 Hz, 1H), 2.67 (s, 3H), 2.57–2.49 (m, 4H), 1.97 (s_v. broad_, 2H), 1.86–1.70 (m, 6H), 1.63–1.53 (m, 2H)**. (^13^C NMR, 100 MHz, CDCl_3_, 300 K)** 174.5, 172.3, 164.8, 163.4, 157.6, 143.3, 142.6, 141.2, 139.2, 131.0, 130.4, 118.3, 58.2, 54.2, 53.4, 53.4, 52.9, 41.0, 37.5, 30.1, 26.0, 21.8. **LCMS:** C_29_H_36_N_8_O_3_S (M calculated) 576.71, C_29_H_37_N_8_O_3_S (MH^+^ found) 577.

**5l (^1^H NMR, 400 MHz, CDCl_3_, 300 K)** 9.14 (s, 1H), 8.32 (s, 1H), 8.23 (d, *J* = 8.4 Hz, 1H), 7.17 (d, *J* = 4.9 Hz, 4H), 6.37 (s, 2H), 5.91 (s_broad_, 1H), 5.59 (s_broad_, 1H), 5.53 (s_broad_, 1H), 5.23 (s, 2H), 4.78 (q, *J* = 8.3 Hz, 1H), 3.59 (s_broad_, 2H), 3.47 (s_broad_, 2H), 3.43 (s, 2H), 3.14–3.10 (m, 2H), 2.58 (s, 3H), 2.49–2.38 (m, 2H), 1.86 (s_v. broad_, 4H), 1.76–1.70 (m, 4H), 1.51–1.37 (m, 4H), 0.90 (d, *J* = 6.8 Hz, 2H), 0.81 (d, *J* = 6.6 Hz, 2H)**. (^13^C NMR, 100 MHz, CDCl_3_, 300 K)** 174.5, 172.4, 164.7, 163.4, 157.6, 143.3, 142.6, 141.2, 139.2, 131.0, 130.4, 118.3, 105.4, 58.3, 54.2, 53.6, 41.4, 40.2, 38.8, 37.5, 34.6, 32.1, 31.1, 29.3, 28.4, 24.9, 22.6, 21.8, 18.9. **LCMS:** C_31_H_40_N_8_O_3_S (M calculated) 604.29, C_31_H_41_N_8_O_3_S (MH^+^ found) 605.

**5m (^1^H NMR, 400 MHz, CDCl_3_, 300 K)** 7.24–7.05 (m, 10H), 6.32 (s, 1H), 6.17 (d, *J* = 9.1 Hz, 1H), 5.87 (S_broad_, 1H), 5.46 (s_broad_, 1H), 5.23 (s, 2H), 4.56 (q, *J* = 7.5 Hz, 1H), 3.62 (s_broad_, 2H), 3.43 (s_broad_, 2H), 3.34 (s, 2H), 2.97 (s, *J* = 7.1 Hz, 2H), 2.40 (s_v. broad_, 2H), 2.13 (s_v. broad_, 2H), 1.95 (s_broad_, 2H), 1.72 (m, 6H), 1.62 (q, *J* = 15.5 Hz, 8H), 1.35–1.32 (m, 2H), 1.12–1.08 (m, 2H)**. (^13^C NMR, 100 MHz, CDCl_3_, 300 K)** 178.1, 173.1, 170.9, 164.9, 140.7, 139.2, 131.3, 130.4, 128.7, 126.3, 125.3, 118.4, 105.1. **LCMS:** C_38_H_46_N_6_O_3_S (M calculated) 666.88, C_38_H_47_N_6_O_3_S (MH^+^ found) 667.

**5n (^1^H NMR, 400 MHz, CDCl_3_, 300 K)** 7.14 (d, *J* = 5.5 Hz, 4H), 6.55 (s, 2H), 6.38 (s, 1H), 6.18 (d, *J* = 7.8 Hz, 1H), 5.87 (s_broad_, 1H), 5.48 (s_broad_, 1H), 5.23 (s, 2H), 4.56 (q, *J* = 7.7 Hz, 1H), 3.79 (s, 9H), 3.78 (s, 2H), 3.46 (d, *J* = 2.8 Hz, 2H), 2.98 (d, *J* = 6.7 Hz, 2H), 2.48 (s_v.broad_, 2H), 1.95 (s_broad_, 2H), 1.72 (s_broad_, 7H), 1.63 (q, *J* = 15.8 Hz, 7H)**. (^13^C NMR, 100 MHz, CDCl_3_, 300 K)** 178.1, 173.1, 170.0, 165.0, 153.3, 139.4, 139.1, 131.4, 131.1, 130.4, 118.4, 105.4, 104.6, 60.9, 58.3, 56.3, 53.6, 40.7, 39.1, 37.3, 36.4, 28.0. **LCMS:** C_38_H_48_N_6_O_6_S (M calculated) 716.89, C_38_H_48_N_6_O_6_S (MH^+^ found) 717.

**5o (^1^H NMR, 400 MHz, CDCl_3_, 300 K)** 7.23 (q, *J* = 10.6 Hz, 4H), 6.46 (s, 1H), 6.31 (d, *J* = 8.7 Hz, 1H), 6.05 (s_broad_, 1H), 5.69 (s_broad_, 1H), 5.32 (s, 1H), 4.67 (q, *J* = 6.7 Hz, 1H), 3.76–3.65 (m, 2H), 3.62–3.56 (m, 2H), 3.53 (d, *J* = 6.7 Hz, 2H), 3.01 (d, *J* = 6.3 Hz, 2H), 2.89 (pent. *J* = 7.9 Hz, 1H), 2.55 (s_broad_, 2H), 2.51 (s_broad_, 2H), 2.04 (s_broad_, 4H), 1.82 (s, 9H), 1.72 (q, *J* = 14.1 Hz, 9H), 1.60–1.50 (m, 2H)**. (^13^C NMR, 100 MHz, CDCl_3_, 300 K)** 178.1, 174.5, 173.2, 165.0, 139.2, 131.3, 130.4, 118.4, 105.3, 58.3, 53.6, 53.5, 52.9, 45.3, 41.6, 41.0, 40.7, 39.1, 37.4, 36.4, 30.1, 28.0, 26.0. **LCMS:** C_34_H_46_N_6_O_3_S (M calculated) 618.83, C_34_H_47_N_6_O_3_S (MH^+^ found) 619.

**5p (^1^H NMR, 400 MHz, CDCl_3_, 300 K)** 7.27 (d, *J* = 15.5 Hz, 4H), 6.46 (s, 1H), 6.23 (d, *J* = 8.4 Hz, 1H), 5.87 (s_broad_, 1H), 5.42 (s_broad_, 1H), 4.65 (q, *J* = 8.4 Hz, 1H), 3.68 (s_broad_, 2H), 3.56 (s_broad_, 2H), 3.52 (pent., *J* = 6.9 Hz, 1H), 3.13–3.1 (m, 2H), 2.54 (s_broad_, 4H), 2.05 (s, 4H), 1.82 (s, 9H), 1.73 (q, *J* = 14.7 Hz, 6H), 1.58–1.47 (m, 6H), 1.30 (s, 2H), 1.23 (t, *J* = 7.3 Hz, 2H), 1.0 (d, *J* = 5.5 Hz, 3H), 0.94–0.86 (m, 3H)**. (^13^C NMR, 100 MHz, CDCl_3_, 300 K)** 178.1, 173.1, 139.1, 131.4, 130.4, 118.5, 105.3, 65.8, 58.3, 53.6, 40.7, 39.1, 38.8, 37.3, 36.4, 31.6, 31.1, 28.4, 28.0, 24.9, 22.6, 18.9, 15.2, 14.1. **LCMS:** C_36_H_50_N_6_O_3_S (M calculated) 646.89, C_36_H_51_N_6_O_3_S (MH^+^ found) 647.

**5q (^1^H NMR, 400 MHz, CDCl_3_, 300 K)** 7.31 (d, *J* = 7.5 Hz, 2H), 7.24–7.20 (m, 5H), 7.16 (d, *J* = 7.3 Hz, 2H), 6.41 (s, 1H), 6.17 (d, *J* = 7.2 Hz, 1H), 5.96 (s_broad_, 1H), 5.60 (s_broad_, 1H), 4.69 (q, *J* = 7.2 Hz, 1H), 3.72 (s_broad_, 2H), 3.50 (q, *J* = 7.7 Hz, 4H), 3.44 (s, 2H), 3.07 (d, *J* = 8.3 Hz, 2H), 2.50 (s_broad_, 2H), 2.51 (s_broad_, 2H), 2.16 (dd, J_AB_ = 7.0 Hz, J_BC_ = 7.6 Hz, 1H), 2.03 (dd, J_AB_ = 8.7 Hz, J_BC_ = 6.5 Hz, 1H), 1.89–1.80 (m, 2H), 1.49–1.40 (m, 5H), 1.33–1.26 (m, 3H), 1.23 (t, *J* = 7.2 Hz, 4H), 1.21–1.16 (m, 3H), 1.15–1.06 (m, 3H), 1.06–0.98 (m, 2H), 0.93–0.86 (m, 3H)**. (^13^C NMR, 100 MHz, CDCl_3_, 300 K)** 173.0, 172.6, 171.0, 164.8, 140.7, 139.1, 131.2, 130.3, 128.7, 126.3, 125.3, 118.3, 65.8, 58.1, 53.8, 52.6, 43.5, 41.0, 38.9, 37.7, 37.4, 36.7, 35.2, 31.6, 29.8, 29.4, 28.5, 22.6, 15.2, 15.2, 14.1. **LCMS:** C_36_H_44_N_6_O_3_S (M calculated) 640.84, C_36_H_45_N_6_O_3_S (MH^+^ found) 641.

**5r (^1^H NMR, 400 MHz, CDCl_3_, 300 K)** 7.23 (d, *J* = 9.2 Hz, 4H), 6.64 (s, 2H), 6.47 (s, 1H), 6.20 (t, *J* = 7.4 Hz, 1H), 5.97 (s_broad_, 1H), 5.63 (s_broad_, 1H), 4.69 (q, *J* = 8.0 Hz, 1H), 3.88 (s, 9H), 3.87 (s, 2H), 3.55 (q, *J* = 7.5 Hz, 3H), 3.50 (Q, *J* = 7.0 Hz, 1H), 3.06 (d, *J* = 8.0 Hz, 2H), 2.57 (s_v.broad_, 4H), 2.24–2.18 (m, 1H), 2.16 (dd, J_AB_ = 4.2 Hz, J_BC_ = 3.8 Hz, 1H), 2.13 (s, 1H), 2.04 (dd, J_AB_ = 3.4 Hz, J_BC_ = 4.2 Hz, 1H), 1.95 (s,1H), 1.90–1.80 (m, 4H), 1.51–1.42 (m, 3H), 1.33–1.26 (m, 2H), 1.23 (t, *J* = 7.2 Hz, 3H), 1.20–0.97 (m, 6H), 0.93–0.86 (m, 1H)**. (^13^C NMR, 100 MHz, CDCl_3_, 300 K)** 173.0, 172.6, 170.1, 153.3, 139.4, 139.2, 131.2, 131.1, 130.3, 118.3, 118.2, 105.4, 104.6, 60.9, 58.3, 56.3, 53.8, 43.5, 41.1, 41.0, 38.9, 37.7, 37.5, 36.7, 35.2, 35.2, 29.8, 28.5. **LCMS:** C_36_H_46_N_6_O_6_S (M calculated) 690.85, C_36_H_47_N_6_O_6_S (MH^+^ found) 691.

**5s (^1^H NMR, 400 MHz, CDCl_3_, 300 K)** 7.24 (q, *J* = 7.2 Hz, 4H), 6.46 (s, 1H), 6.17 (t, *J* = 7.7 Hz, 1H), 5.91 (s_broad_, 1H), 5.56 (s_broad_, 1H), 4.69 (q, *J* = 7.3 Hz, 1H), 3.73–3.66 (m, 2H), 3.62–3.55 (m, 2H), 3.54–3.78 (m, 3H), 3.10–3.05 (m, 2H), 2.90 (pent *J* = 7.9 Hz, 1H), 2.58–2.47 (m, 4H), 2.24–2.13 (m, 2H), 2.07–1.99 (m, 1H), 1.95 (s_broad_, 1H), 1.90–1.70 (m, 13H), 1.65–1.54 ( m, 2H), 1.52–1.42 (m, 4H), 1.34–1.07 (m, 8H), 0.94–0.86 (m, 1H). **(^13^C NMR, 100 MHz, CDCl_3_, 300 K)** 174.5, 173.0, 139.2, 131.1, 130.3, 118.3, 105.2, 75.0, 65.8, 58.4, 53.8, 53.5, 52.9, 45.4, 43.5, 41.7, 41.1, 41.0, 38.9, 37.7, 37.5, 36.7, 35.2, 31.5, 30.8, 30.1, 29.8, 28.5, 26.0, 15.2, 14.0. **LCMS:** C_32_H_44_N_6_O_3_S (M calculated) 592.80, C_32_H_45_N_6_O_3_S (MH^+^ found) 593.

**5t (^1^H NMR, 400 MHz, CDCl_3_, 300 K)** 7.23 (q, *J* = 12.7 Hz, 4H), 6.46 (s, 1H), 6.24 (t, *J* = 9.5 Hz, 1H), 6.0 (d, *J* = 7.4 Hz, 1H), 6.68 (s_broad_, 1H), 4.70 (q, *J* = 7.4 Hz, 1H), 3.67 (s_v. broad_, 2H), 3.59–3.48 (m, 4H), 3.06 (d, *J* = 7.4 Hz, 2H), 2.54 (s_broad_, 2H), 2.50 (s_broad_, 2H), 2.23 (s_broad_, 2H), 2.19 (s, 2H), 2.17–2.13 (m, 1H), 2.07–1.99 (m, 1H), 1.95 (s_borad_, 2H), 1.87 (s_broad_, 2H), 1.85–1.70 (m, 6H), 1.57–1.42 (m, 8H), 1.32–1.06 (m, 7H), 0.99 (d, *J* = 7.5 Hz, 3H), 094–0.88 (m, 3H). **(^13^C NMR, 100 MHz, CDCl_3_, 300 K)** 174.5, 173.0, 172.6, 139.2, 131.1, 130.3, 118.3, 118.2, 105.3, 58.3, 53.8, 45.2, 43.5, 41.1, 41.0, 40.2, 39.0, 38.8, 37.7, 37.5, 36.7, 35.2, 31.1, 29.8, 29.3, 28.5, 28.4, 24.9, 22.6, 18.9. **LCMS:** C_34_H_48_N_6_O_3_S (M calculated) 620.85, C_34_H_49_N_6_O_3_S (MH^+^ found) 621.

**5u (^1^H NMR, 400 MHz, CDCl_3_, 300 K)** 7.29 (d, *J* = 6.40 Hz, 2H), 7.22 (d, *J* = 3.4 Hz, 4H), 7.17 (d, *J* = 7.4 Hz, 2H), 6.41 (s, 1H), 6.22 (d, *J* = 8.9 Hz, 1H), 5.94 (s_broad_, 1H), 5.62 (s_broad_, 1H), 4.69 (q, *J* = 8.9 Hz, 1H), 3.71 (s_broad_, 2H), 3.51 (pent, *J* = 7.8 Hz, 2H), 3.44 (d, *J* = 4.3 Hz, 2H), 3.06 (t, *J* = 5.8 Hz, 2H), 2.50 (s_broad_, 2H), 2.20 (t, *J* = 5.3 Hz, 5H), 1.61 (pent, *J* = 7.3 Hz, 2H), 1.44–1.41 (m, 2H), 1.35–1.22 (m, 6H), 1.22–1.17 (m, 2H), 0.89 (t, *J* = 6.6 Hz, 4H). **(^13^C NMR, 100 MHz, CDCl_3_, 300 K)** 173.2, 173.0, 170.9, 164.7, 140.7, 139.2, 131.1, 130.3, 128.7, 126.3, 125.3, 118.3, 105.3, 58.2, 53.9, 52.6, 37.6, 36.5, 31.3, 29.4, 25.2, 22.3, 15.2, 13.9. **LCMS:** C_33_H_42_N_6_O_3_S (M calculated) 602.79, C_33_H_43_N_6_O_3_S (MH^+^ found) 603.

**5v (^1^H NMR, 400 MHz, CDCl_3_, 300 K)** 7.23 (q, *J* = 7.6 Hz, 4H), 6.64 (s, 2H), 6.46 (s, 1H), 6.22 (d, *J* = 7.6 Hz, 1H), 5.93 (s_broad_, 1H), 5.63 (s_broad_, 1H), 4.69 (q, *J* = 8.1 Hz, 1H), 3.88 (s, 6H), 3.87 (s, 3H), 3.55 (d, *J* = 4.5 Hz, 2H), 3.53–3.48 (m, 1H), 3.09–3.04 (m, 2H), 2.56 (s_v.broad_, 4H), 2.20 (t, *J* = 6.7 Hz, 3H), 1.61 (pent, *J* = 8.9 Hz, 1H), 1.36–1.21 (m, 6H), 0.89 (t, *J* = 6.7 Hz, 4H)**. (^13^C NMR, 100 MHz, CDCl_3_, 300 K)** 173.2, 173.0, 170.1, 153.3, 139.4, 139.1, 131.2, 131.1, 130.3, 118.3, 104.6, 60.9, 58.2, 56.3, 53.9, 37.5, 36.5, 31.3, 25.2, 22.3, 13.9. **LCMS:** C_33_H_44_N_6_O_6_S (M calculated) 652.80, C_33_H_45_N_6_O_6_S (MH^+^ found) 653.

**5w (^1^H NMR, 400 MHz, CDCl_3_, 300 K)** 7.27 (d, *J* = 7.0 Hz, 2H), 7.20 (d, *J* = 7.7 Hz, 2H), 6.45 (s, 2H), 6.30 (d, *J* = 9.8 Hz, 1H), 6.02 (s_broad_, 1H), 5.76 (s_broad_, 1H), 4.70 (q, *J* = 7.7 Hz, 1H), 3.76–3.65 (m, 2H), 3.61–3.55 (m, 2H), 3.52 (d, *J* = 11.5 Hz, 2H), 3.09–3.03 (m, 2H), 2.89 (pent, *J* = 8.5 Hz, 1H), 2.54 (s_broad_, 2H), 2.50 (s_broad, 2H),_ 2.21 (t, *J* = 7.7 Hz, 2H), 2.19 (s, 2H), 1.88–1.72 (7H), 1.66–1.55 (m, 5H), 1.36–1.21 (m, 5H), 0.89 (t, *J* = 6.6 Hz, 3H)**. (^13^C NMR, 100 MHz, CDCl_3_, 300 K)** 174.5, 173.2, 173.1, 164.7, 148.7, 139.3, 131.0, 130.3, 118.2, 105.3, 58.3, 53.9, 53.5, 52.9, 45.3, 41.6, 41.0, 37.6, 36.5, 31.3, 30.8, 30.1, 26.0, 25.2, 22.3, 13.9. **LCMS:** C_29_H_42_N_6_O_3_S (M calculated) 554.75, C_29_H_43_N_6_O_3_S (MH^+^ found) 555.

**5x (^1^H NMR, 400 MHz, CDCl_3_, 300 K)** 7.26 (d, *J* = 8.0 Hz, 2H), 7.20 (d, *J* = 8.7 Hz, 2H), 6.45 (s, 1H), 6.27 (d, *J* = 8.0 Hz, 1H), 5.97 (s_broad_, 1H), 5.71 (s_broad_, 1H), 4.69 (q, *J* = 9.8 Hz, 1H), 3.67 (s_vbroad_, 2H), 3.59–3.46 (m, 4H), 3.06 (t, *J* = 5.4 Hz, 2H), 2.55 (s_broad_, 2H), 2.50 (s_broad_, 2H), 2.21 (d, *J* = 7.3 Hz, 1H), 2.19 (s, 3H), 1.90 (S_v.broad_, 2H), 1.85–1.72 (m, 5H), 1.65–1.47 (m, 7H), 1.34–1.23 (m, 5H), 0.99 (d, *J* = 7.9 Hz, 3H), 0.89 (t, *J* = 7.1 Hz, 3H)**. (^13^C NMR, 100 MHz, CDCl_3_, 300 K)** 174.5, 173.2, 173.0, 164.7, 148.8, 139.2, 131.1, 130.3, 118.2, 105.3, 58.4, 53.9, 40.2, 38.8, 37.6, 36.5, 34.6, 32.1, 30.9, 29.3, 28.4, 25.2, 24.9, 22.6, 22.3, 18.9, 13.9. **LCMS:** C_31_H_46_N_6_O_3_S (M calculated) 582.80, C_31_H_47_N_6_O_3_S (MH^+^ found) 583.

### 3.2. P. falciparum Culture and Antiplasmodial Activity Assay

*P. falciparum* Dd2 (multi-drug-resistant) cells were cultured using a modified Trager and Jensen method [68] in RPMI 1640 medium containing l-glutamine (Invitrogen, Carlsbad, CA, USA) and supplemented with 25 mM HEPES, pH 7.4, 26 mM NaHCO_3_, 2% dextrose, 15 mg/L hypoxanthine, 25 mg/L gentamycin, and 0.5% Albumax II in human A^+^ erythrocytes. Cultures were incubated at 37 °C in a humidified environment of 5% CO_2_ and 95% air. Serial dilutions of the compound in DMSO were added to the *P. falciparum* culture at a 1% parasitemia and 2% hematocrit in 96-well plates. The maximum DMSO concentration did not exceed 0.125%. Following incubation for 72 h at 37 °C, the inhibition of parasite growth was determined by a SYBR green I-based fluorescent assay, which measures the DNA content of the parasite [63,64,65]. Plates were frozen at −80 °C and thawed, followed by the addition of the lysis buffer (100 µL, 20 mM Tris-HCl, 0.08% saponin, 5 mM EDTA, 0.8% Triton X-100, and 0.01% SYBR Green I) to each well. Following incubation in the dark for 30 min at 37 °C, the fluorescence emission from the samples was measured using a Synergy H4 multimode plate reader (Biotek, Winooski, VT, USA) at wavelengths of 485 nM for excitation and 530 nM for emission. A reduction in fluorescent signal compared to the control reflects a reduction in DNA content that resulted from inhibition of growth.

### 3.3. Cytotoxicity Assay

Serial dilutions of compounds were used to determine cytotoxicity [69] in HepG2 human hepatocyte cells (2500 cells/well) in 384 well clear bottom plates (Santa Cruz Biotechnology, Dallas, TX, USA). The plates were incubated for 48 h at 37 °C in a humidified atmosphere (5% CO_2_, 95% air)_._ Following the addition of 20 µL MTS ((3-(4,5-dimethylthiazol-2-yl)-5-(3-carboxymethoxyphenyl)-2-(4-sulfophenyl)-2*H*-tetrazolium), CellTiter 96^®^ Aqueous cell proliferation assay, Promega, Tokyo, Japan) reagent to each well, the plates were further incubated for an additional 3 h, and cell viability was determined by measurement of the absorbance at 490 nm using Synergy H4 plate reader (Biotek, Winooski, VT, USA).

### 3.4. Stage-Specific Inhibition Assays

*P. falciparum* Dd2 cultures were tightly synchronized employing magnetic separation of schizonts [70], followed by sorbitol lysis [71]. Synchronized cultures were exposed to the hit compound at 5 × EC_50_ at 6, 18, 30, and 42 h post-invasion. At 12 h time intervals following treatment, samples were collected for flow cytometric cell cycle analysis. Samples were fixed in 0.04% glutaraldehyde in PBS, permeabilized with 0.25% Triton X-100, treated with RNAse (50 µg/mL), and stained with 10.24 µM YOYO-1 DNA-binding dye (Invitrogen) [72]. YOYO-1 is highly fluorescent when intercalated with double-stranded DNA. Flow cytometry acquisition was conducted in a CytoFLEX flow cytometer (Beckman Coulter, Indianapolis, IN, USA) at an excitation wavelength of 488 nM and an optical filter of 530/30. The data were analyzed using the Cytexpert program.

## Figures and Tables

**Table 1:** Solid-phase Synthesis of aminothiazole tethered piperazines. Representative examples.

Entry Library 2291	R^1^	R^2^	Yield (mg) ^a^	Purity (%) ^b^
**5a**	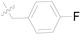	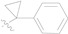	82	92%
**5b**	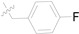	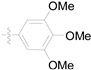	75	94%
**5c**	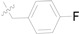	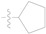	70	76%
**5d**	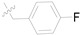	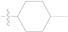	77	100%
**5e**	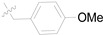	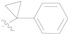	73	86%
**5f**	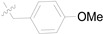	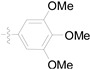	80	90%
**5g**	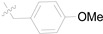	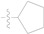	76	71%
**5h**	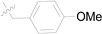	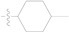	79	100%
**5i**	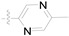	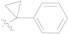	87	89%
**5j**	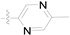	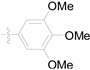	79	88%
**5k**	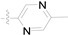	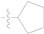	77	85%
**5l**	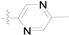	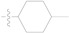	74	88%
**5m**	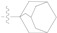	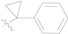	72	100%
**5n**	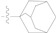	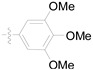	89	96%
**5o**	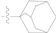	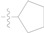	81	75%
**5p**	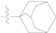	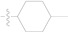	79	100%
**5q**	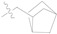	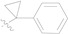	79	86%
**5r**	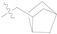	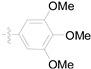	82	90%
**5s**	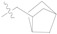	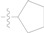	88	86%
**5t**	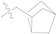	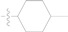	82	83%
**5u**	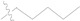	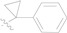	83	100%
**5v**	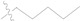	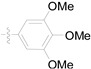	80	95%
**5w**	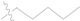	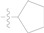	83	90%
**5x**	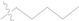	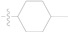	76	95%

^a^ based on crude yield. ^b^ Crude samples were analyzed on a Vydac column with a gradient of 5–95% formic acid in MeCN over 7 min. The purity was based on the analytical traces at λ = 214 and 254 nm.

## Data Availability

Data contained within the article and Supplementary Materials.

## Supplementary Materials

The following supporting information can be downloaded at: https://www.mdpi.com/article/10.3390/ijms242417414/s1.

## Abbreviations

ESI-MS	electron spray ionization mass spectrometry
^1^H NMR	H-nuclear magnetic resonance
^13^C NMR	C (isotope 13)-nuclear magnetic resonance
LC-MS	liquid chromatography coupled to mass spectrometry
RP-HPLC	reverse phase high-performance liquid chromatography
TFA	trifluoroacetic acid
UV	ultraviolet
DCM	dichloromethane
THF	tetrahydrofuran
HF	hydrogen fluoride
TKI	tyrosine kinase inhibitor
DMF	dimethylformamide
DIEA	diisopropylethylamine
