# Supplementary material for: Parallel Synthesis of Piperazine Tethered Thiazole Compounds with Antiplasmodial Activity
Source: Int J Mol Sci. 2023 Dec 12;24(24):17414. doi: 10.3390/ijms242417414 (PMC10743568; doi:10.3390/ijms242417414)
Supplement: Supplementary file 1 [file ijms-24-17414-s001.zip › ijms-2750626-supplementary.pdf]

Parallel Synthesis of Piperazine Tethered Thiazole Compounds

with Antiplasmodial Activity

Ramanjaneyulu Rayala [1], Prakash Chaudhari [1], Ashley Bunnell [1],

Bracken Roberts[2], Debopam Chakrabarti[2], and Adel Nefzi [1*]

[1] Herbert Wertheim College of Medicine, Center for Translational Science, Florida International University

[2] Division of Molecular Microbiology, Burnett School of Biomedical Sciences, University of Central Florida, Orlando, FL 32826, USA

Supporting Information

Table of Contents:

Materials and General Methods	1
Synthesis (6)	2 - 5
Characterization Data	6 - 78
Antiplasmadoil activity	79- 90

Materials and General Methods:

Reagents and solvents were purchased from various commercial sources (Sigma Aldrich, Chemimpex, VWR, Cambridge Isotopes, etc.) and used without further purification unless otherwise stated. Yields were based on the manufacturer's reported loading of MBHA resin of 1.15 mmole per gram of resin. ^{1}H NMR spectra were recorded at 400 MHz and ^{13}C NMR were recorded at 100 MHz in deuterated chloroform and all chemical shifts are reported in δ units relative to TMS. All L-amino acids used were assumed to be enantiomerically pure from the supplier. LC-MS was performed on crude samples dissolved in 50:50 (acetonitrile and water) at a concentration of 1 mg/ mL on a Shimadzu LC-MS equipped with a Vydac column with a gradient of 5 – 95 % formic acid in acetonitrile over 7 minutes with their UV traces monitored at $\lambda = 214$ and 254 nm. HPLC purification was performed on a Phenomenex Luna 150 x 21.2 mm 5 micron column with a flow rate of 12 mL/min.

Synthesis of compounds 2291:

- **Bag preparation and resin functionalization**

A 100-mg sample of p-methylbenzhydrylamine hydrochloride (MBHA ·HCl) resin (CHEM-IMPEX INTERNATIONAL) with a molar loading of 1.15 mmole/g and size of 100 -200 mesh, was thermally sealed into a polypropylene mesh bag. All bags were made in the same fashion. The bags were washed in triplicate with dichloromethane, 5 % diisopropylethylamine in dichloromethane, and finally dichloromethane to neutralize the acidic resin. The neutralized resin bags were then treated with 3 equivalents of Boc-Phe(4-NO_2)-OH (3.2 grams), HOBt (1.6 grams) and DIC (2.1 mL) in 75 mL of DMF in a 150-mL polystyrene bottle. The bottle was placed on a mechanical shaker and left for approximately 15 hours. A ninhydrin test was used on a few grains of the resin to confirm the complete acylation of the resin amine.

- **Boc Deprotection**

The Boc protecting group was routinely removed from the N-terminus by shaking the tea bags in a 55 % solution of trifluoroacetic acid in dichloromethane for 30 minutes. Upon completion of the TFA treatment, the bags were thoroughly washed with dichloromethane (x3), neutralized with 5 % DIEA in dichloromethane (x 4) and washed with DCM (x3). Upon addition of the DIEA, the resin within the tea bags typically changes from a red/ orange color to a yellow/ white color.

- **N-Terminus Acylation**

The N-terminus was acylated using different carboxylic acids. All acylations were carried out with 10 equivalents of the acid, and 10 equivalents of DIC in DMF. The reactions were allowed to shake overnight (~15 hours) before they were thoroughly washed with DMF followed by DCM. A ninhydrin test of a few grains of resin confirmed that all of the acylations were complete.

- **Stannous Chloride Reduction**

All bags were combined into a 150-mL polystyrene bottle. Stannous chloride (20 eq, 13.1 grams) was added to the bottle along with 69 mL of DMF to afford a concentration of 1.0 M. The bottle was gently sonicated for approximately 20 minutes to ensure a homogenous solution and then set

to shaking overnight (~15 hours). In the morning the bags were washed 12 times with DMF, 6 times with MeOH and 6 times with DCM to ensure complete removal of the tin. A few milligrams of resin were then removed from the bag acylated with 4-Fluoropheyl acetic acid and used as a control to confirm complete reduction by cleavage in HF for 1.5 hours. LC-MS of the resulting product confirmed the reduction was complete.

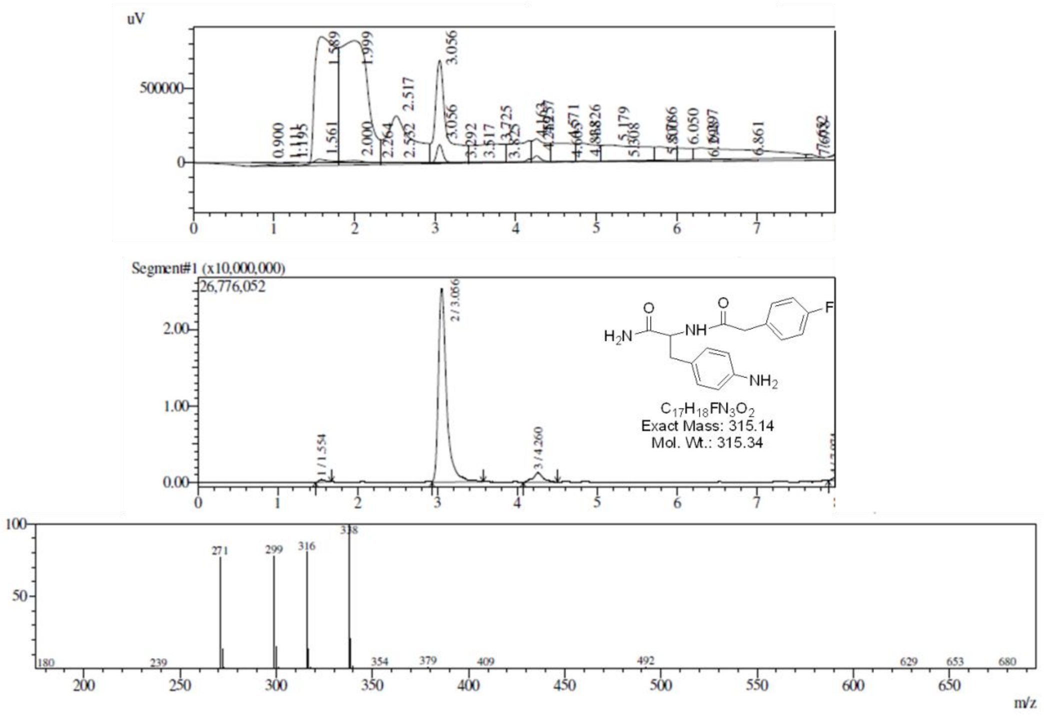

- **Installation of Fmoc Protected thio-amide**

All bags were combined in a 150-mL polystyrene bottle. Fmoc-NCS (2.5 equivalents) was weighed out quickly and added to the polystyrene bottle. The bottle was purged with nitrogen gas for approximately 10 minutes before anhydrous DMF (~ 60 mL) was added via canula. The bottle was covered and sealed with parafilm and then sonicated for ~10 minutes to ensure a homogenous solution. The bottle was shaken on a mechanical shaker overnight. In the morning, the bags were thoroughly washed with DMF and DCM.

- **Fmoc Cleavage**

All bags were combined in a 150-mL polystyrene bottle. The bottle was then filled half way with a 20 % solution of piperidine in DMF. The bottle was allowed to shake for 10 minutes at room temperature before the solution was poured off and the procedure repeated. The bags were then thoroughly washed with DMF and DCM to ensure complete removal of the piperidine.

- **Hantzch's Cyclization**

All 30 bags were sealed into a mesh bag and then added to a reflux glassware equipped with a condensing column and heating mantle. The system was purged with nitrogen for 10 minutes before 10 equivalents of 1,3-dichloroacetone was added. 115 mL of anhydrous DMF was added via canula with all bags submerged below the solvent. An internal thermometer was added and the

cyclization was carried out at 85 °C overnight. In the morning the colorless solution had turned a dark brown color, which is indicative of this reaction. The bags were thoroughly washed with DMF and DCM and placed on the under vacuum at 0° C until they were used in the next step. A few milligrams of the same bag was again used as a control and cleaved for 1.5 hours in HF. The LC-MS of the resulting product confirmed complete cyclization of the thio-amide.

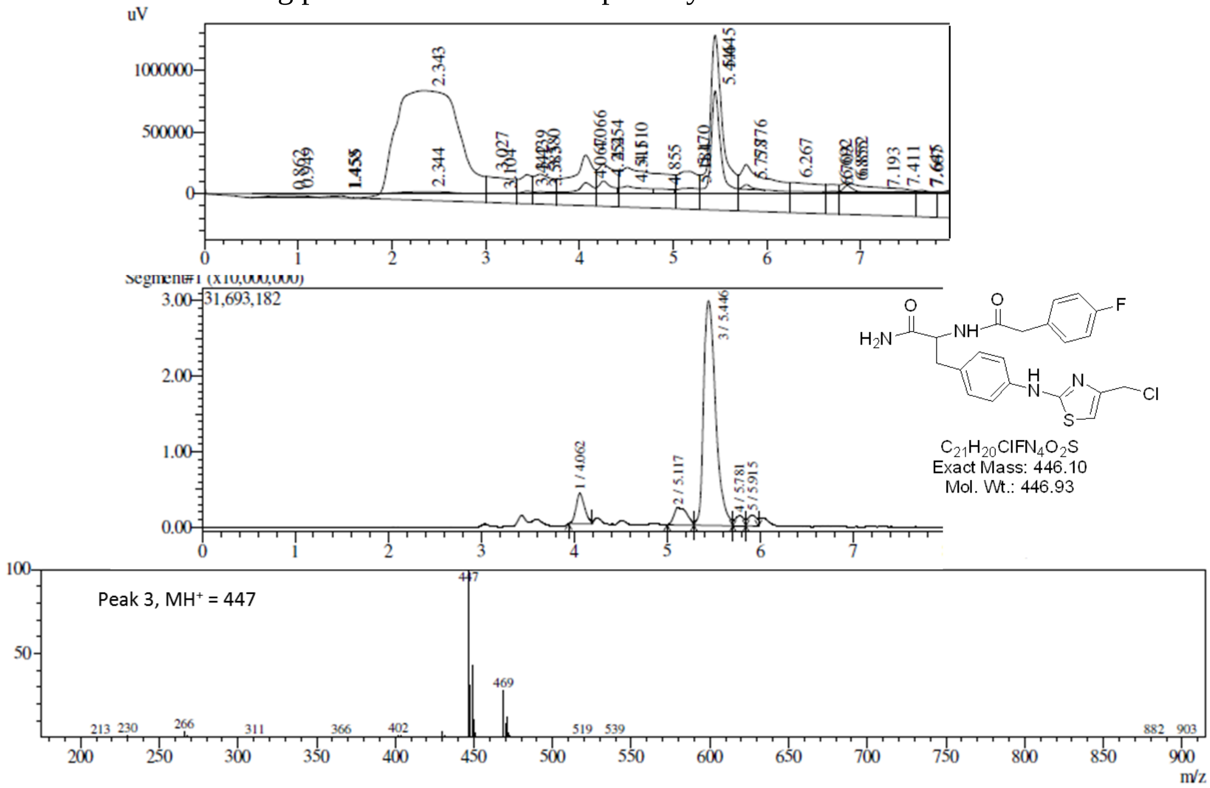

- **Boc-Piperazine Displacement of Chloride**

All bags were added to a 150-mL polystyrene bottle. Boc-piperazine (6 equivalents) was added to the bottle along with 100 mL of DMF. Diisopropylethylamine (6 equivalents, 3.61 mL) was added lastly and the bottle was closed and allowed to shake at room temperature overnight. In the morning, the bags were washed with DMF and DCM. A few milligrams of bag #24 were removed from the main bag, treated with TFA to remove the Boc protecting group and cleaved as a control in HF for 1.5 hours. The LC-MS of the resulting product confirmed complete nucleophilic displacement of the chloride.

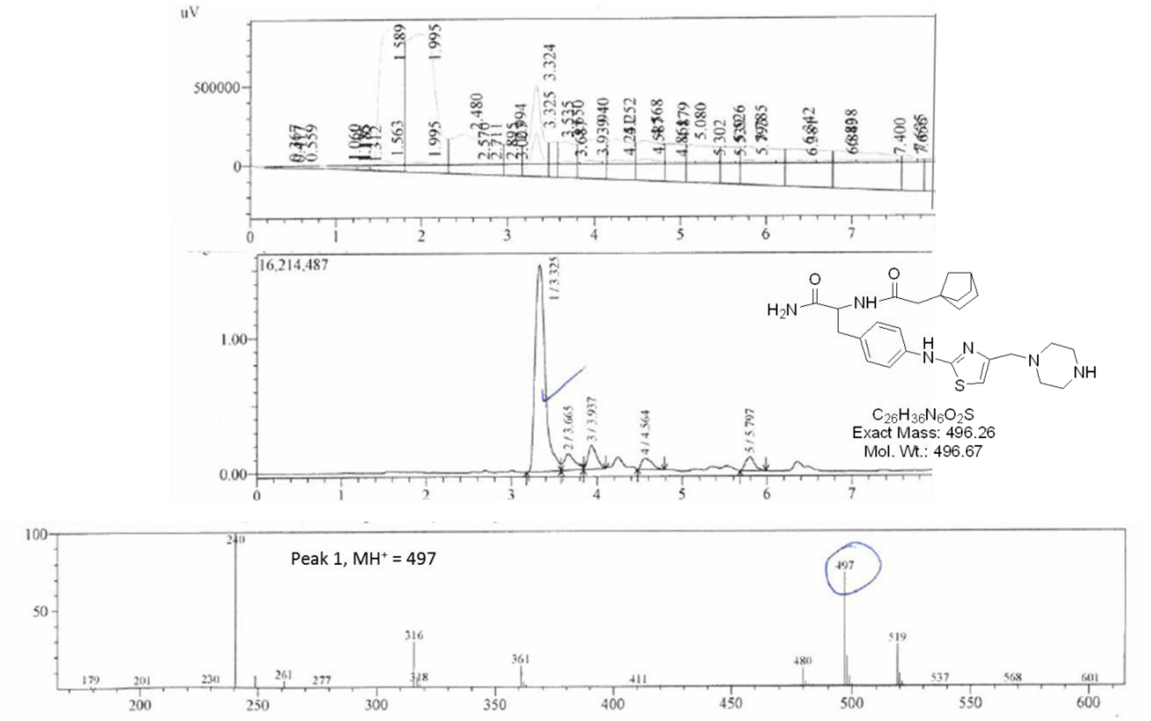

- **Boc Protecting Group Cleavage**

The Boc protecting group was removed from the piperazine by shaking the tea bags in a 55 % solution of trifluoroacetic acid in dichloromethane for 30 minutes, twice. Upon completion of the second TFA treatment, the bags were thoroughly washed with dichloromethane (x3), neutralized with 5 % DIPEA (x 4) in dichloromethane and washed with DCM (x3). Upon addition of the DIPEA, the resin within the teabags typically changes from a red/ orange color to a yellow/ white color.

- **Piperazine Acylation**

The piperazine was acylated using different carboxylic acids such that bags. All acylations were carried out with 10 equivalents of the acid, and 10 equivalents of DIC in 25 mL of THF. The reactions were allowed to shake overnight (~15 hours) before they were thoroughly washed with DMF followed by DCM.

- **Cleavage from the Solid Support**

The dried bags were treated with hydrofluoric acid at 0 ºC for 1.5 hours before the HF was blown-off with nitrogen. The bags were then extracted for 1 hour in 5 mL of 95 % acetic acid. The extraction was repeated twice. The acetic acid extracts were then transferred to glass scintillation flasks, frozen and lyophilized. The crude samples were taken up in 6 – 8 mL of 50:50 acetonitrile and water and a sample was submitted for LC-MS analysis as a 1 mg/ mL sample. The crude samples were frozen and lyophilized again. This procedure was repeated for a total of three times. Upon completion of the third round, the crude samples were transferred to tared glass scintillation vials, frozen and lyophilized a final time. The final crude yields were obtained by subtracting the mass of the empty vial from the mass of the filled vial.

Product Characterization (5)

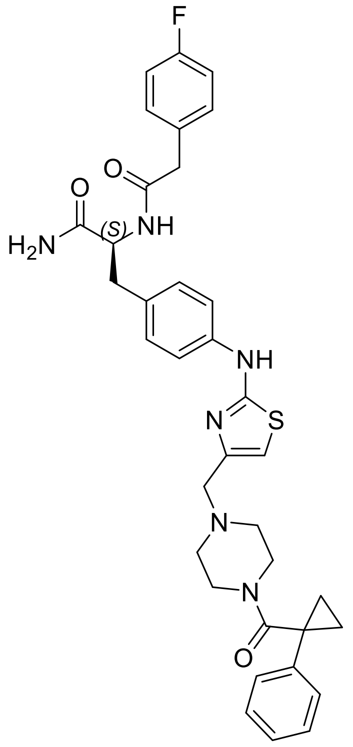

5a
$C_{35}H_{37}FN_6O_3S$
Exact Mass: 640.26
Mol. Wt.: 640.77

5a (^{1}H NMR, 400 MHz, $CDCl_3$, 300 K) δ 7.30 – 7.27 (m, 2H), 7.21 – 7.11 (m, 7H), 7.07 – 6.98 (m, 4H), 6.44 (s, 1H), 6.16 (d, J = 10.0 Hz, 1H), 5.94 ($s_{(broad)}$, 1H), 5.61 ($s_{(broad)}$, 1H), 4.64 (q, J = 9.3 Hz, 1H), 3.76 ($s_{(broad)}$, 2H), 3.51 (s, 2H), 2.17 (s, 2H), 2.98 (d, J = 8.6 Hz, 2H), 2.54 ($s_{(broad)}$, 2H), 2.24 ($s_{(broad)}$, 2H), 1.42 (m, 2H), 1.18 (m, 2H)

5a (^{13}C NMR, 100 MHz, $CDCl_3$, 300 K) δ 172.6, 171.0, 170.8, 164.7, 140.6, 139.2, 130.9, 130.8, 130.7. 130.2, 128.7, 126.4, 125.3, 118.2, 116.0, 115.8, 77.2, 58.8, 53.9, 52.5, 42.6, 37.2, 29.3, 15.1

LCMS: $C_{35}H_{37}FN_6O_3S$ (M calculated) 640.77, $C_{35}H_{38}FN_6O_3S$ (MH^+ found) 641

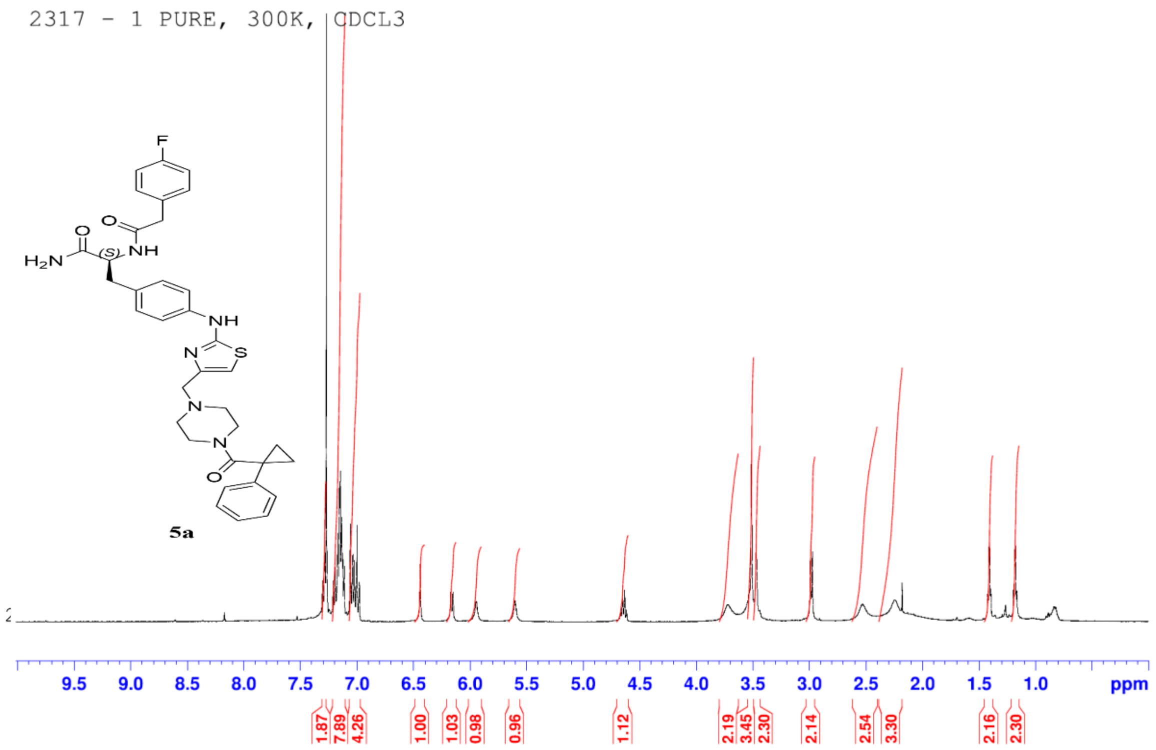
2317 - 1 PURE, 300K, CDCL3
F
O
NH
H2N
(S)
NH
N
S
N
N
O
5a
9.5 9.0 8.5 8.0 7.5 7.0 6.5 6.0 5.5 5.0 4.5 4.0 3.5 3.0 2.5 2.0 1.5 1.0 ppm
1.87 7.89 4.26 1.00 1.03 0.98 0.96 1.12 2.19 3.45 2.30 2.14 2.54 3.30 2.16 2.30

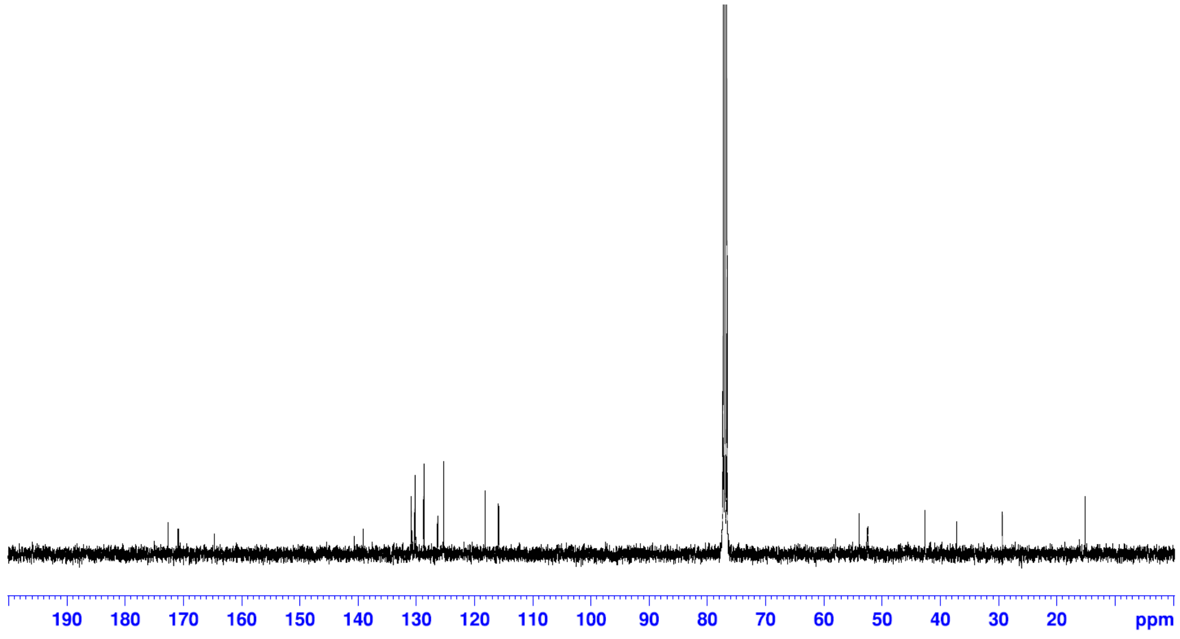
190 180 170 160 150 140 130 120 110 100 90 80 70 60 50 40 30 20 ppm

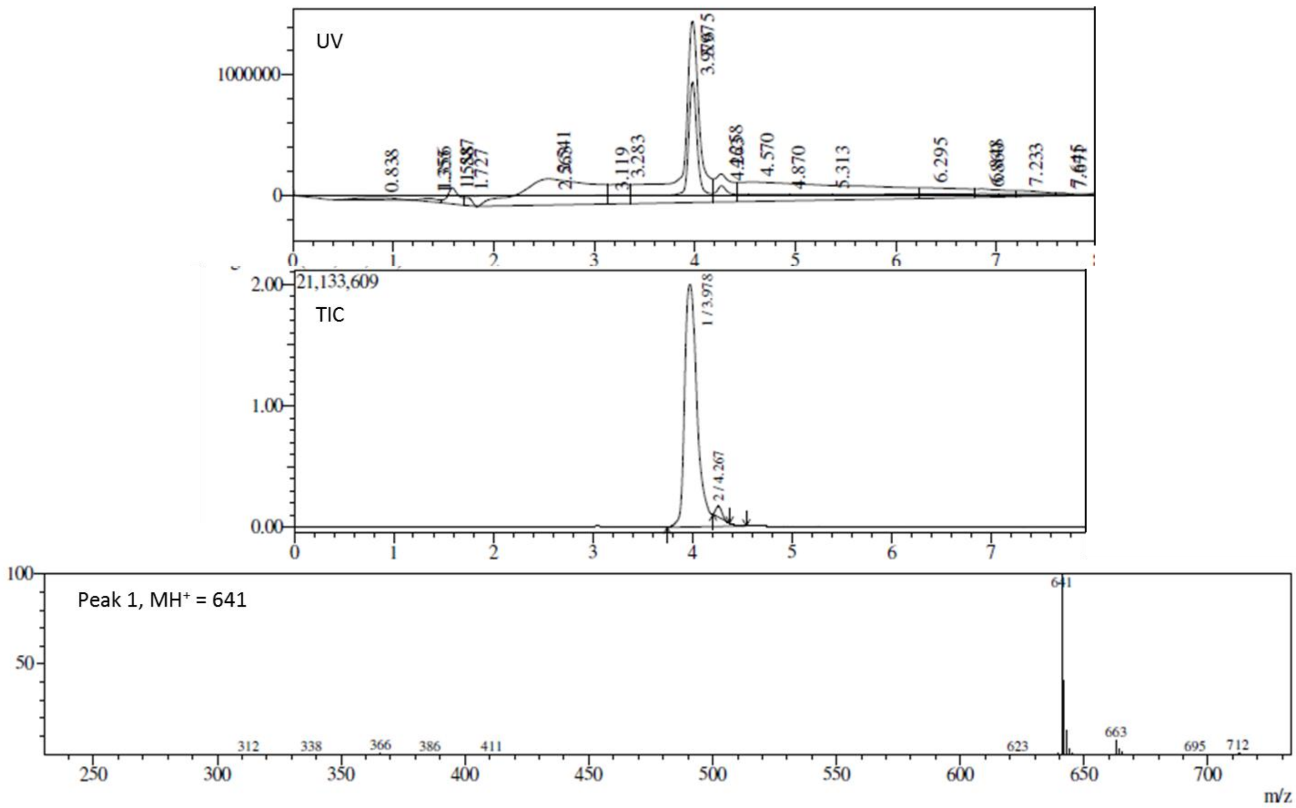
UV
TIC
21,133,609
1 / 3.978
2 / 4.267
Peak 1, MH⁺ = 641
m/z

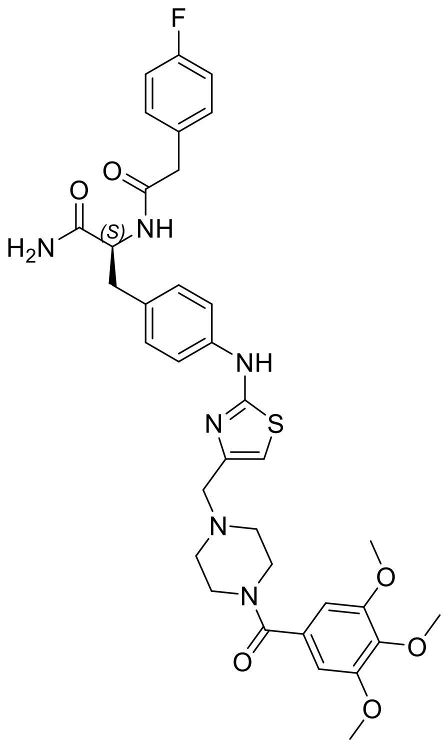

5b

$C_{35}H_{39}FN_6O_6S$

Exact Mass: 690.26

Mol. Wt.: 690.78

5b (^{1}H NMR, 400 MHz, $CDCl_3$, 300 K) δ 7.18 (d, J = 13.3 Hz, 2H), 7.14 (t, J = 3.7 Hz, 2H), 7.06 (d, J = 8.8 Hz, 2H), 7.01 (t, J = 7.4 Hz, 2H), 6.64 (s, 2H), 6.55 ($s_{(broad)}$, 1H), 6.22 (d, J = 8.1 Hz, 1H), 6.03 ($s_{(broad)}$, 1H), 5.66 ($s_{(broad)}$, 1H), 4.66 (q, J = 9.3 Hz, 1H), 3.87 (s, 12H), 3.63 (s, 2H), 3.53 (s, 2H), 3.00 (d J = 8.1 Hz, 2H), 2.65 ($s_{(broad)}$, 4H)

5b (^{13}C NMR, 100 MHz, $CDCl_3$, 300 K) 1 δ 72.7, 170.8, 170.1, 164.7, 160.9, 158.8, 153.4, 139.1, 130.9, 130.8, 130.3, 118.2, 116.0, 115.7, 104.6, 77.2, 60.9, 56.3, 53.9, 42.6, 37.2

LCMS: $C_{35}H_{39}FN_6O_6S$ (M calculated) 690.78, $C_{35}H_{40}FN_6O_6S$ (MH^+ found) 691

2317 - 3 PURE, 300K, CDCL3

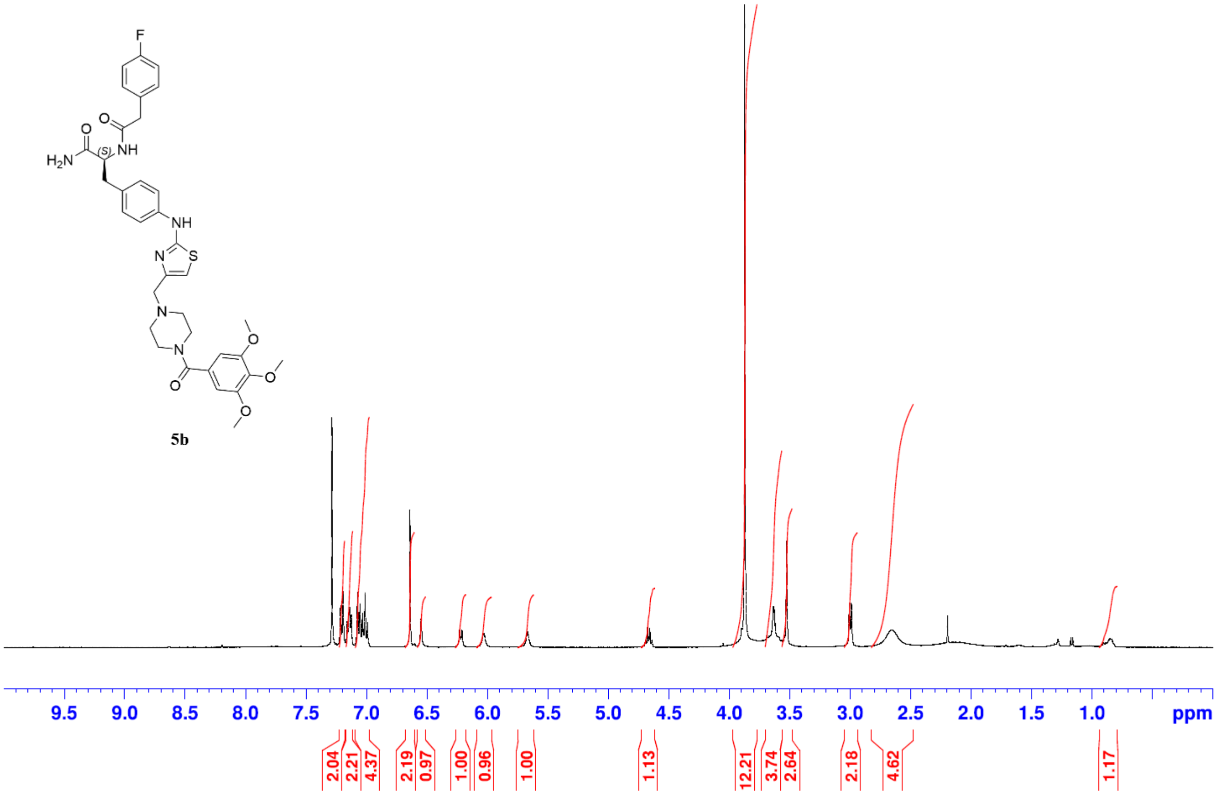

317 - 3 PURE, 300K, CDCL3

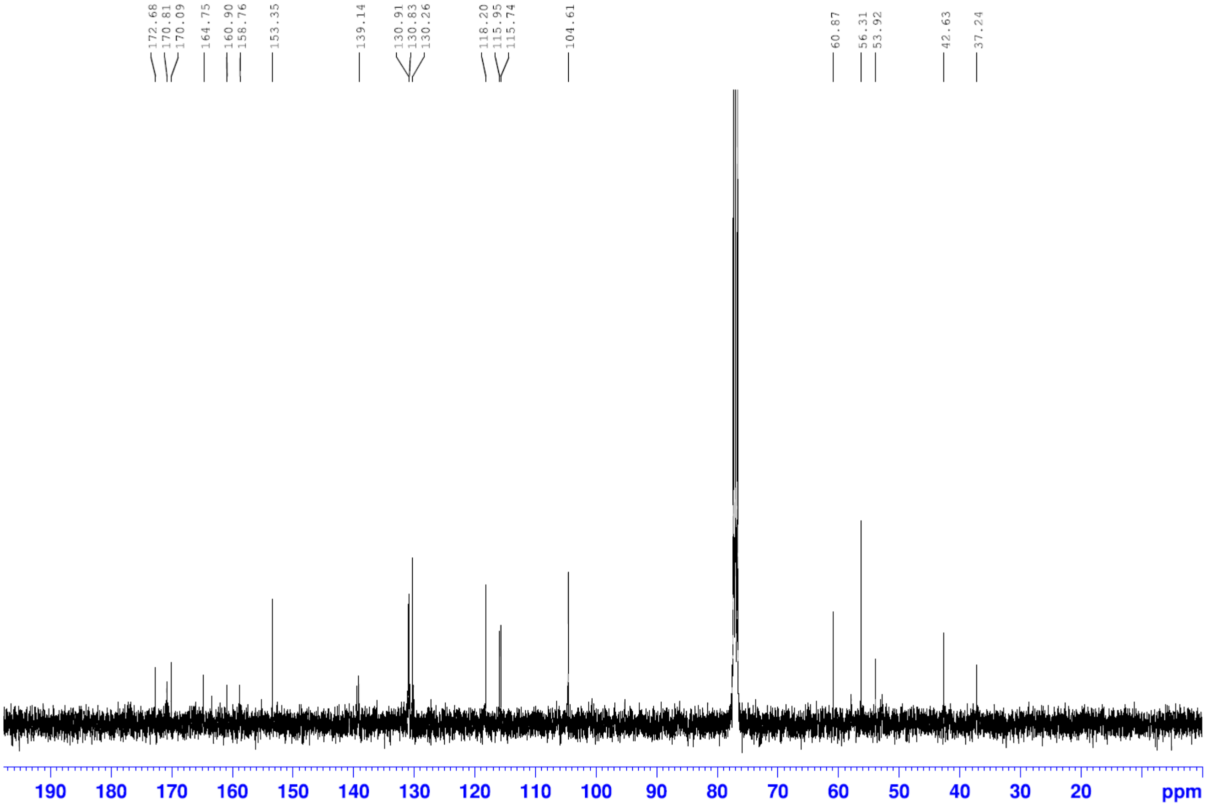

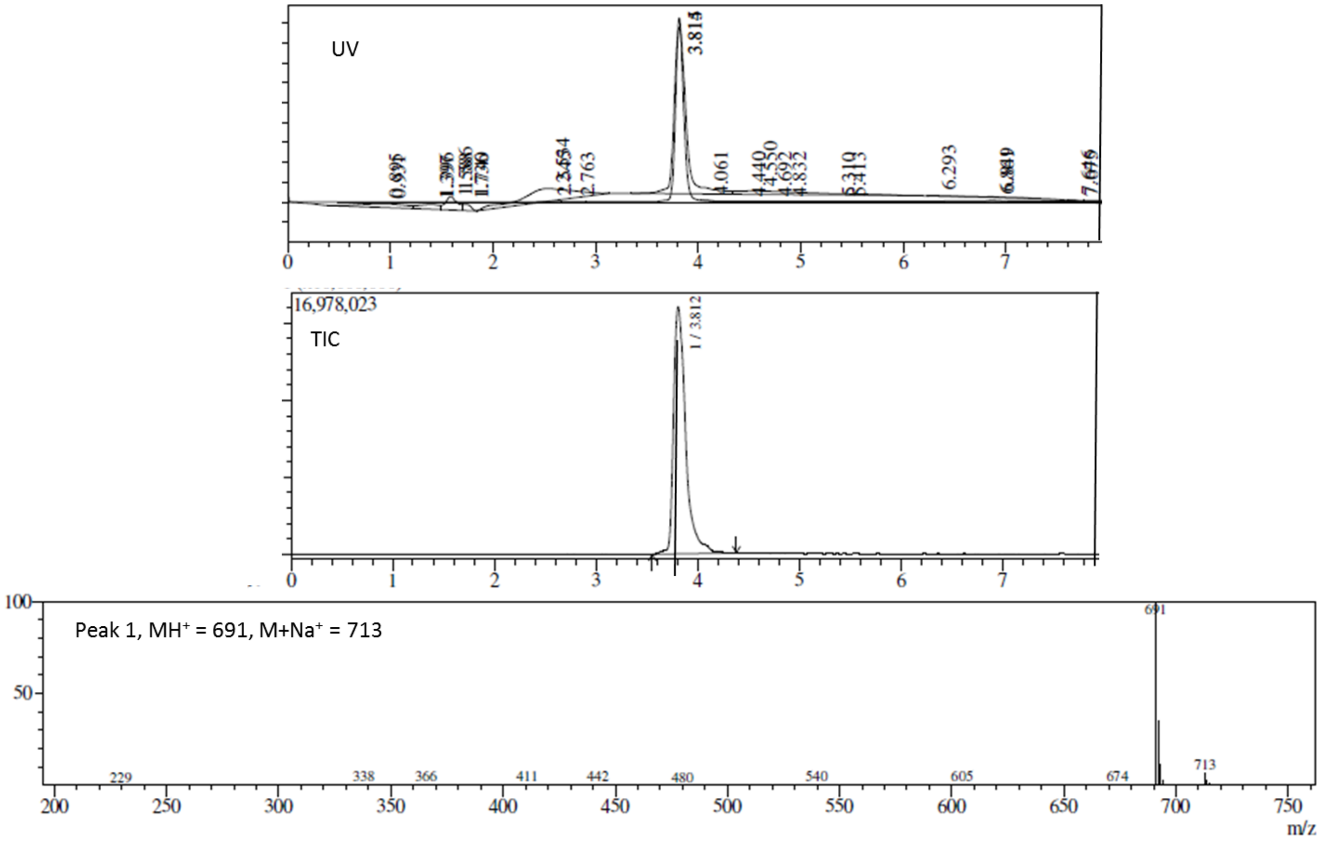
UV
TIC
16,978,023
1 / 3.812
Peak 1, MH⁺ = 691, M+Na⁺ = 713
691
713
m/z

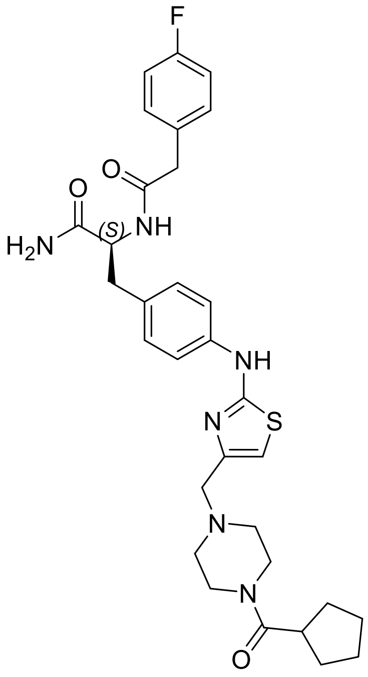

5c
$C_{31}H_{37}FN_6O_3S$
Exact Mass: 592.26
Mol. Wt.: 592.73

5c (^{1}H NMR, 400 MHz, CDCl₃, 300 K) δ 7.20 (d, J = 7.4 Hz, 2H), 7.15 (t, J = 8.2 Hz, 2H), 7.05 (d, J = 8.2 Hz, 2H), 7.00 (t, J = 8.9 Hz, 2H), 6.50 (s, 1H), 6.25 (d, J = 8.2 Hz, 1H), 6.03 ($s_{(broad)}$, 1H), 5.73 ($s_{(broad)}$, 1H), 4.66 (q, J = 7.4 Hz, 1H), 3.71 – 3.68 (m, 2H), 3.67 – 3.58 (m, 2H), 3.56 (d, J = 7.0 Hz, 2H), 3.53 (s, 3H), 3.00 (d, J = 7.8 Hz, 2H), 2.89 (pent. J = 7.0 Hz, 1H), 2.58 – 2.52 (m, 5H), 1.88 – 1.64 (m, 8H), 1.62 – 1.51 (m, 3H)

5c (^{13}C NMR, 100 MHz, CDCl₃, 300 K) δ 174.5, 172.7, 170.8, 164.7, 163.4, 139.2, 130.9, 130.8, 130.6, 130.2, 118.2, 115.9, 115.7, 77.2, 58.2, 53.9, 42.6, 41.0, 37.2, 30.1, 26.0

LCMS: $C_{31}H_{37}FN_6O_3S$ (M calculated) 592.73, $C_{31}H_{38}FN_6O_3S$ (MH^+ found) 593

317 - 4 PURE, 300K, CDCL3

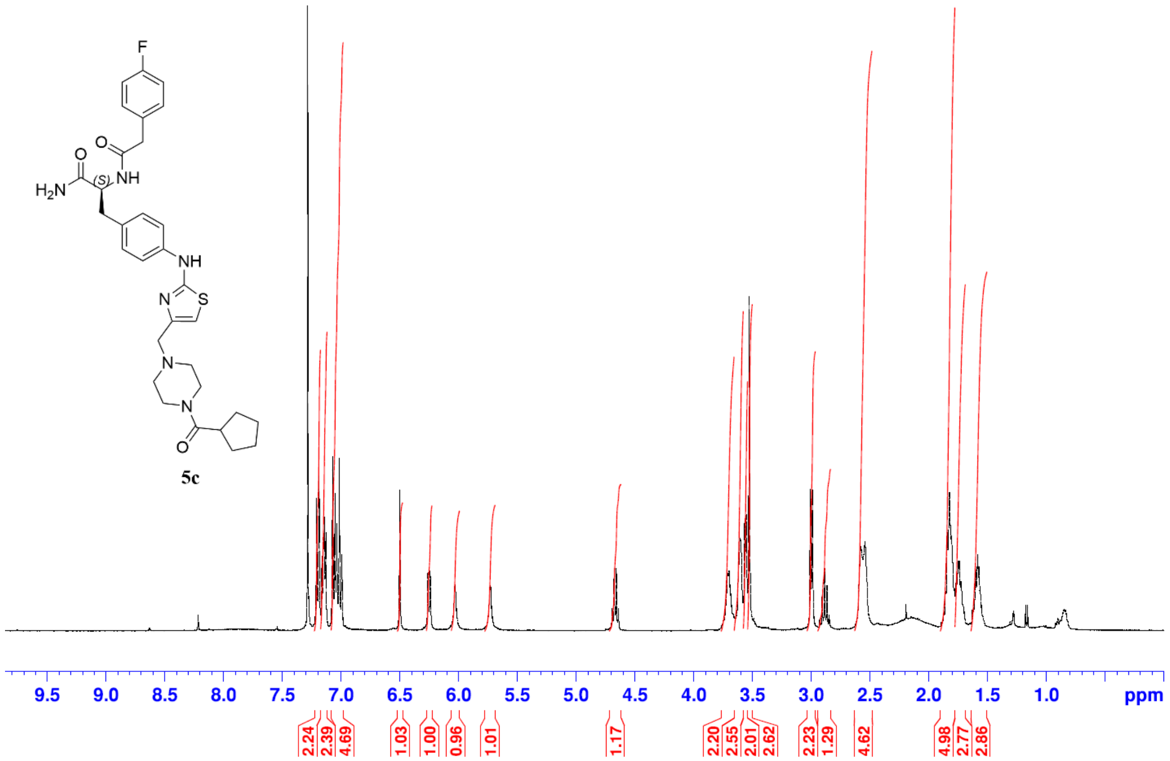

317 - 4 PURE, 300K, CDCL3

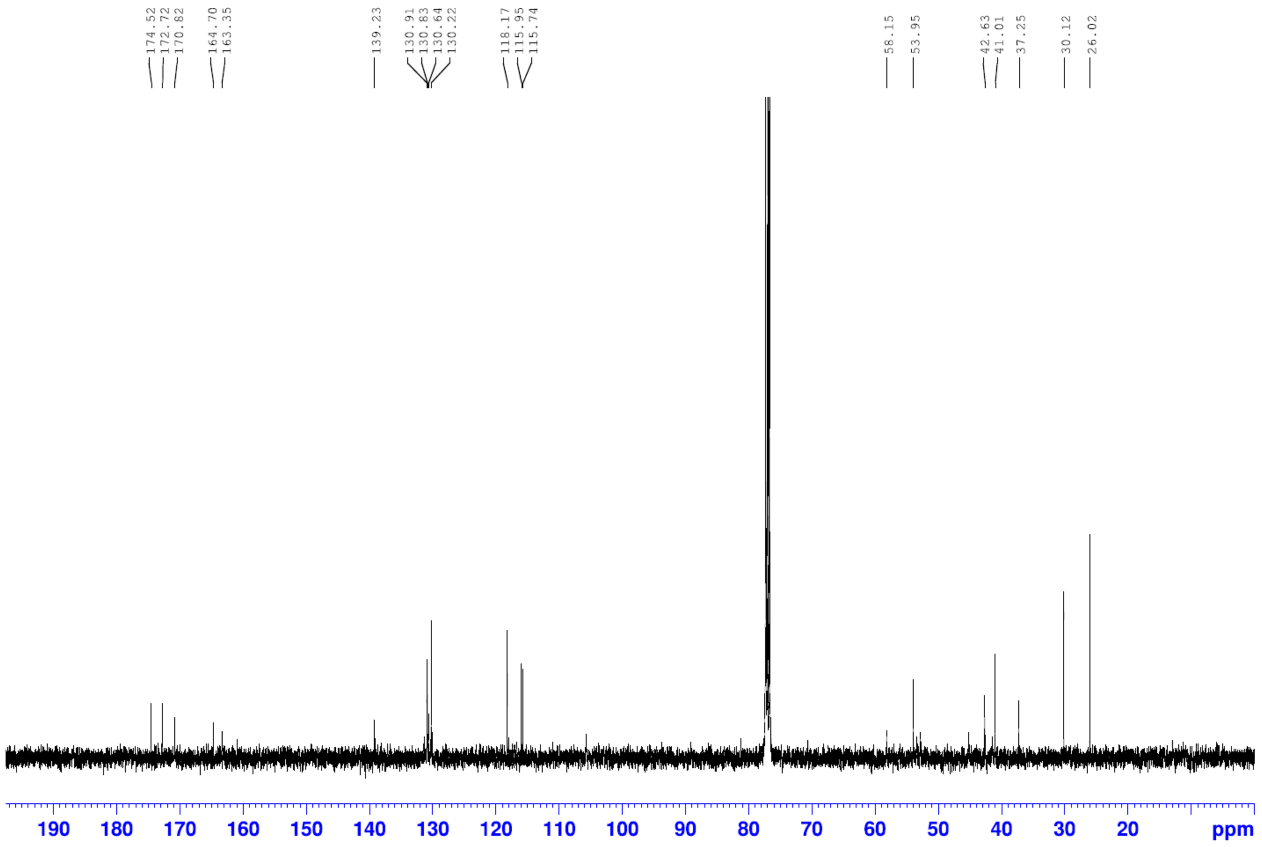

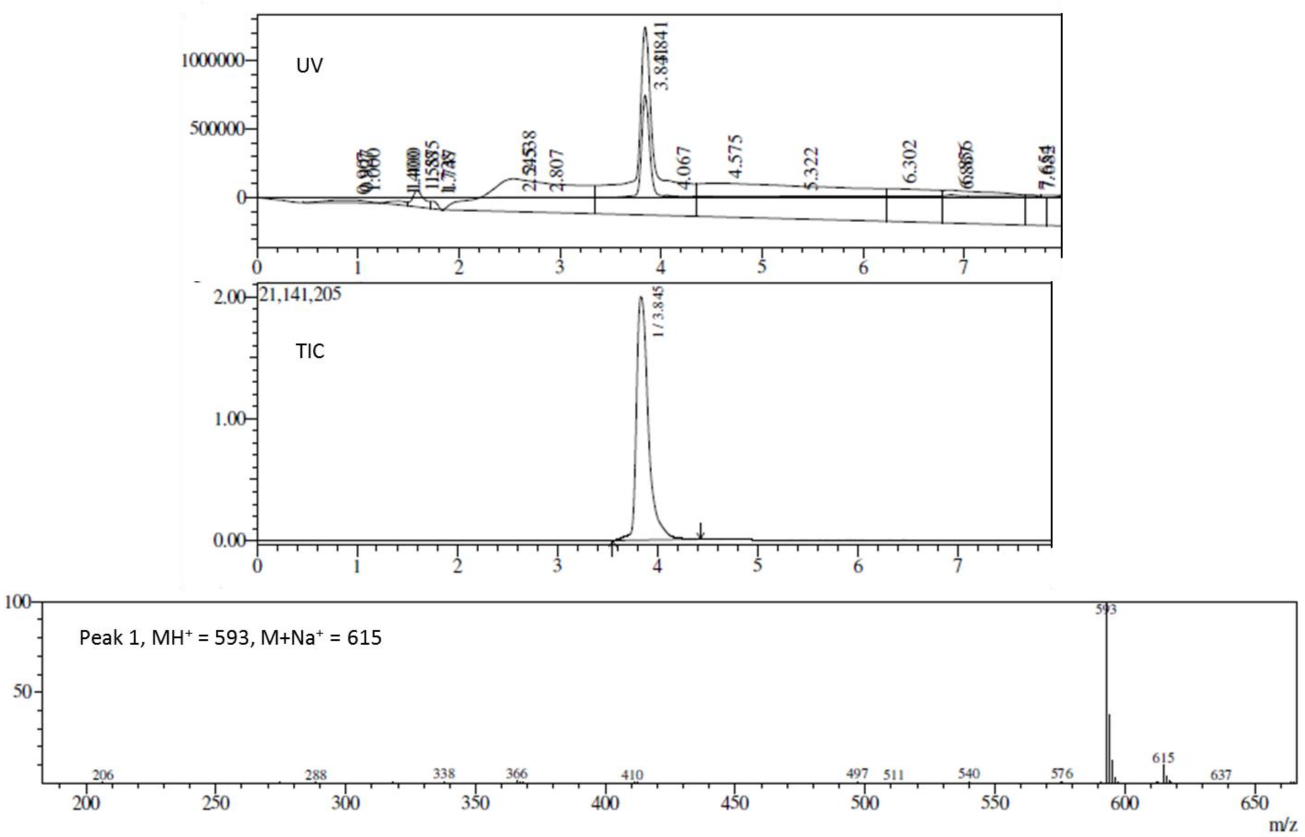
UV
TIC
21,141,205
1 / 3.845
Peak 1, MH+ = 593, M+Na+ = 615
m/z

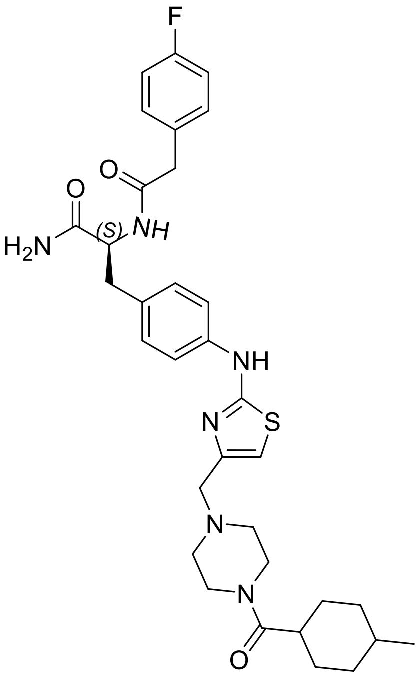

5d
$C_{33}H_{41}FN_6O_3S$
Exact Mass: 620.29
Mol. Wt.: 620.78

5d (¹H NMR, 400 MHz, $CDCl_3$, 300 K) δ 7.20 (d, J = 8.2 Hz, 2H), 7.15 (t, J = 6.7 Hz, 2H), 7.06 (d, J = 8.2 Hz, 2H), 7.01 (t, J = 7.5 Hz, 2H), 6.50 (s, 1H), 6.25 (d, J = 8.2 Hz, 1H), 6.02 ($s_{(broad)}$, 1H), 5.71 ($s_{(broad)}$, 1H), 4.67 (q, J = 8.2 Hz, 1H), 3.71 – 3.68 (m, 2H), 3.67 – 3.58 (m, 4H), 3.53 (s, 3H), 3.00 (d, J = 9.8 Hz, 2H), 2.60 – 2.51 (m, 5H), 1.86 – 1.73 (m, 4H), 1.54 – 1.50 (m, 4H), 0.98 (d, J = 8.9 Hz, 2H), 0.90 (d, J = 7.2 Hz, 2H)

5d (¹³C NMR, 100 MHz, $CDCl_3$, 300 K) δ 174.6, 172.7, 170.8, 164.7, 139.2, 130.9, 130.8, 130.7, 130.2, 118.2, 116.0, 115.7, 105.7, 77.2, 53.9, 42.6, 40.1, 38.8, 37.3, 34.5, 32.1, 31.1, 29.3, 28.4, 24.9, 22.6, 18.9

LCMS: $C_{33}H_{41}FN_6O_3S$ (M calculated) 620.78, $C_{33}H_{42}FN_6O_3S$ (MH^+ found) 621

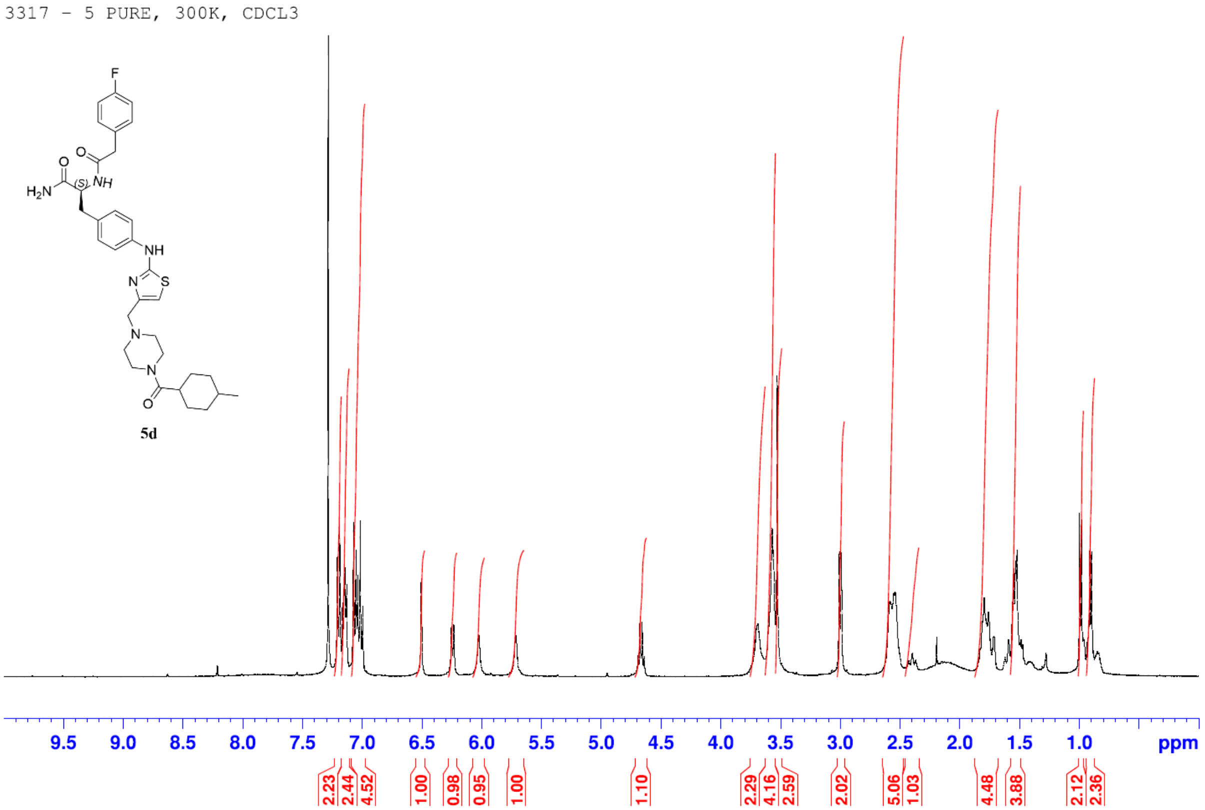
3317 - 5 PURE, 300K, CDCL3
F
O
O
H2N
(S)
NH
NH
N
S
N
N
O
5d
9.5 9.0 8.5 8.0 7.5 7.0 6.5 6.0 5.5 5.0 4.5 4.0 3.5 3.0 2.5 2.0 1.5 1.0 ppm
2.23 2.44 4.52 1.00 0.98 0.95 1.00 1.10 2.29 4.16 2.59 2.02 5.06 1.03 4.48 3.88 2.12 2.36

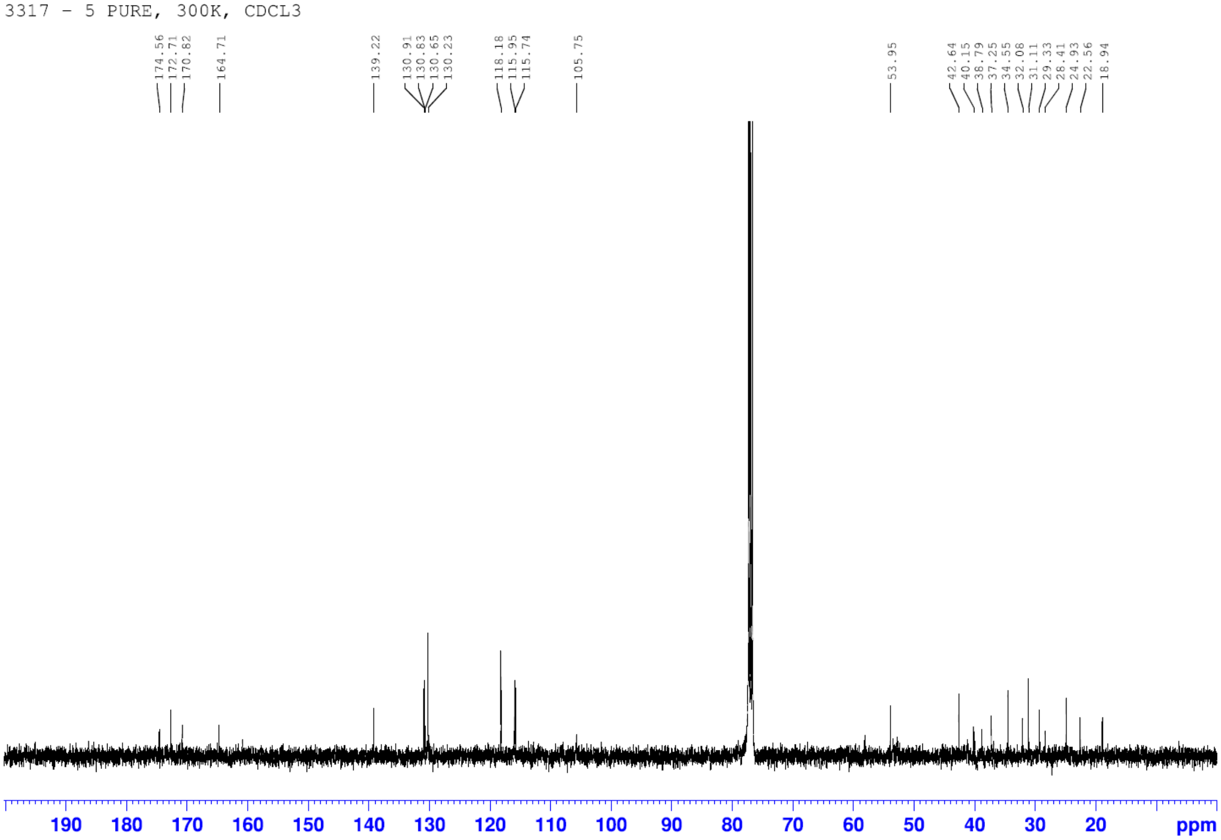
3317 - 5 PURE, 300K, CDCL3
174.56 172.71 170.82 164.71 139.22 130.91 130.83 130.65 130.23 118.18 115.95 115.74 105.75 53.95 42.64 40.15 38.79 37.25 34.55 32.08 31.11 29.33 28.41 24.93 22.56 18.94
190 180 170 160 150 140 130 120 110 100 90 80 70 60 50 40 30 20 ppm

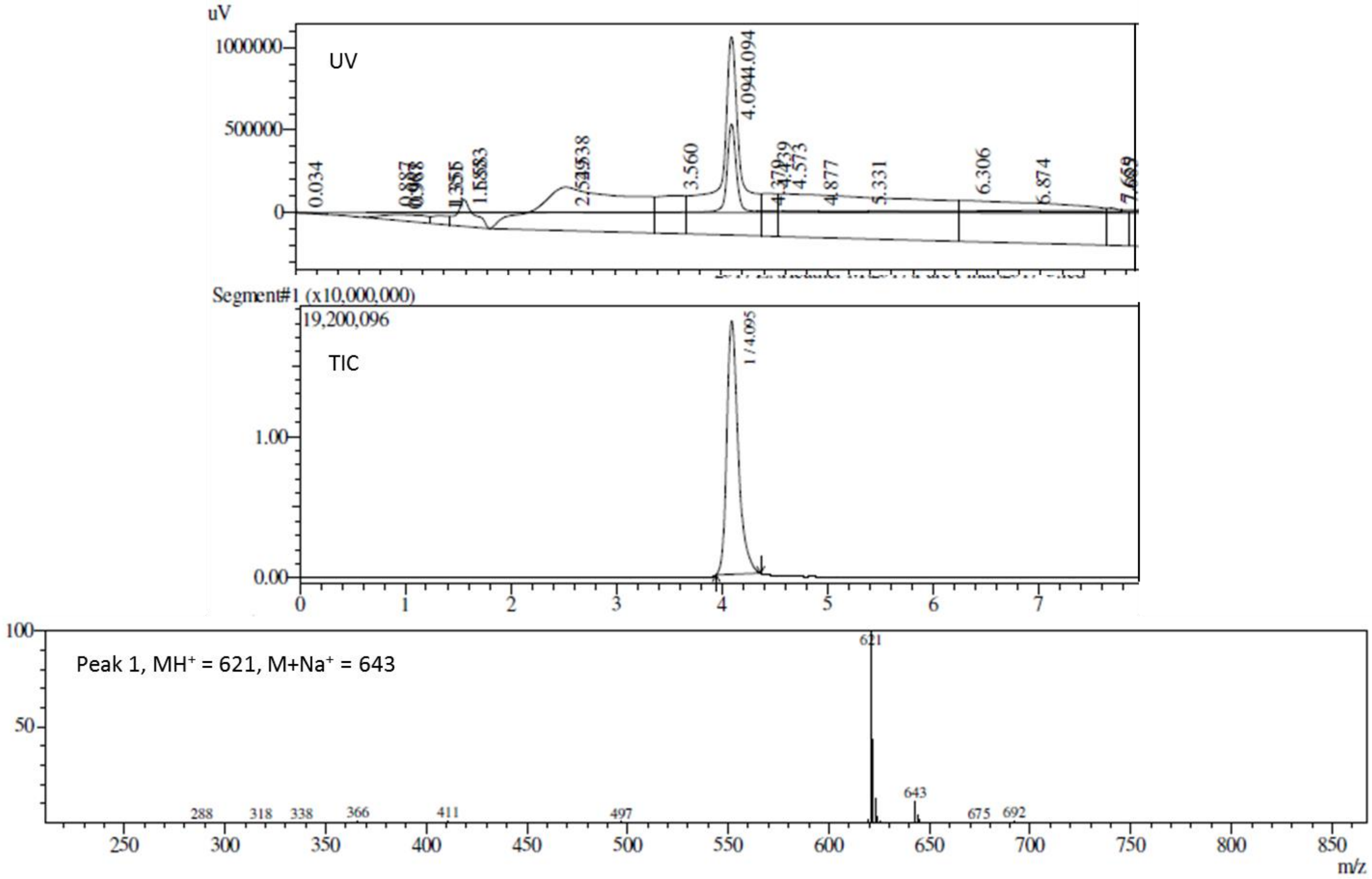
uV
UV
1000000
500000
0
4.094
3.560
6.306
6.874
Segment#1 (x10,000,000)
19,200,096
TIC
1/4.095
1.00
0.00
0 1 2 3 4 5 6 7
Peak 1, MH+ = 621, M+Na+ = 643
100
50
621
643
288 318 338 366 411 497 675 692
250 300 350 400 450 500 550 600 650 700 750 800 850
m/z

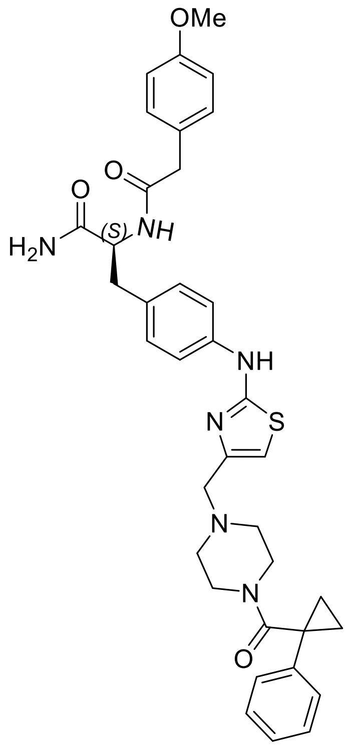

5e
$C_{36}H_{40}N_6O_4S$
Exact Mass: 652.28
Mol. Wt.: 652.81

5e (^{1}H NMR, 400 MHz, CDCl$_3$, 300 K) δ 7.30 (t, J = 6.9 Hz, 1H), 7.21 (d, J = 8.4 Hz, 1H), 7.16 (d, J = 8.4 Hz, 4H), 7.08 (d, J = 6.8 Hz, 2H), 7.05 (d, J = 8.4 Hz, 2H), 6.86 (d, J = 8.4 Hz, 2H), 6.45 (s, 1H), 6.14 (d, J = 9.9 Hz, 1H), 6.10 ($s_{(broad)}$, 1H), 5.65 ($s_{(broad)}$, 1H), 4.66 (q, J = 7.6 Hz, 1H), 3.80 (s, 3H), 3.74 ($s_{(broad)}$, 2H), 3.55 ($s_{(broad)}$, 2H), 3.50 (s, 3H), 2.98 (d, J = 6.5 Hz, 2H), 2.55 ($s_{(broad),}$ 2H), 2.26 ($s_{(broad)}$, 2H), 1.44 – 1.42 (m, 2H), 1.21 – 1.18 (m, 2H)

5e (^{13}C NMR, 100 MHz, CDCl$_3$, 300 K) δ 172.8, 171.6, 171.0, 164.9, 159.0, 140.6, 139.1, 130.9, 130.4, 130.3, 128.7, 126.4, 126.2, 125.3, 118.3, 114.5, 77.2, 57.9, 55.3, 53.9, 52.4, 42.7, 37.0, 29.3, 15.1

LCMS: $C_{36}H_{40}N_6O_4S$ (M calculated) 652.81, $C_{36}H_{41}N_6O_4S$ (MH^+ found) 653

317 - 6 PURE, 300K, CDCL3

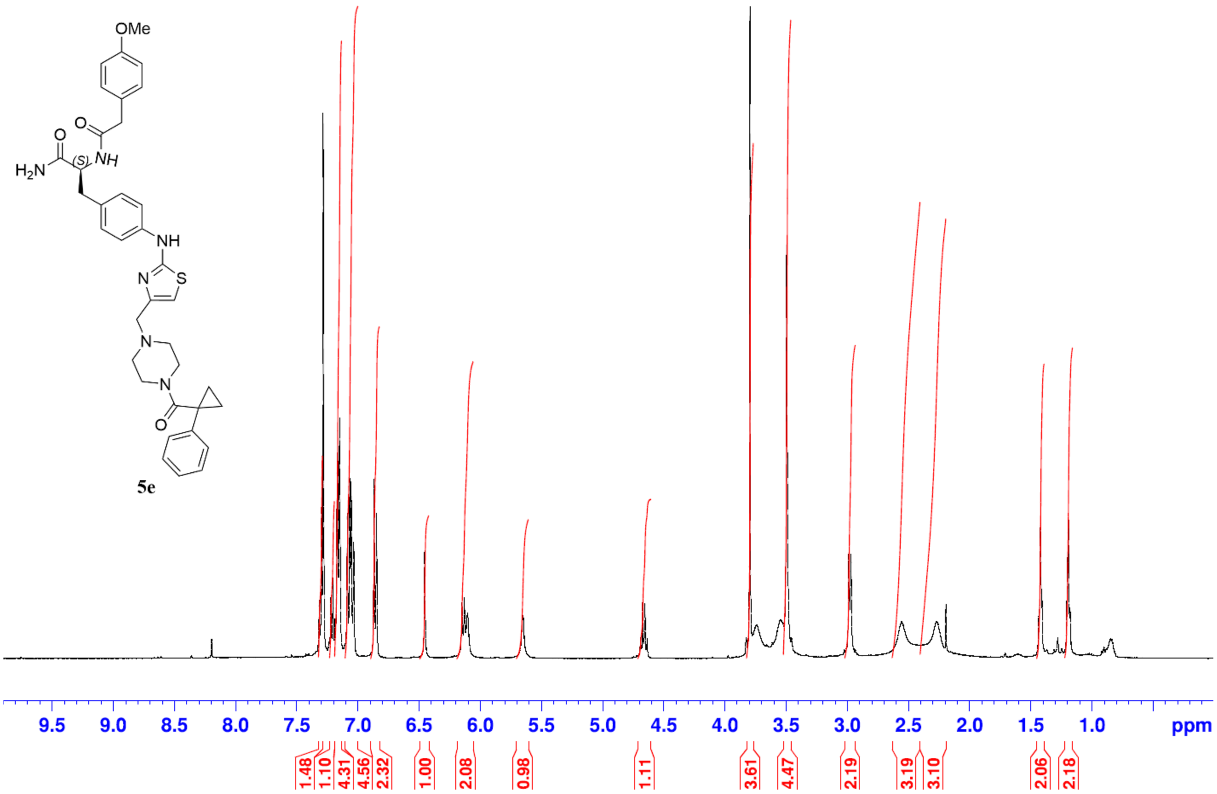

2317 - 6 PURE, 300K, CDCL3

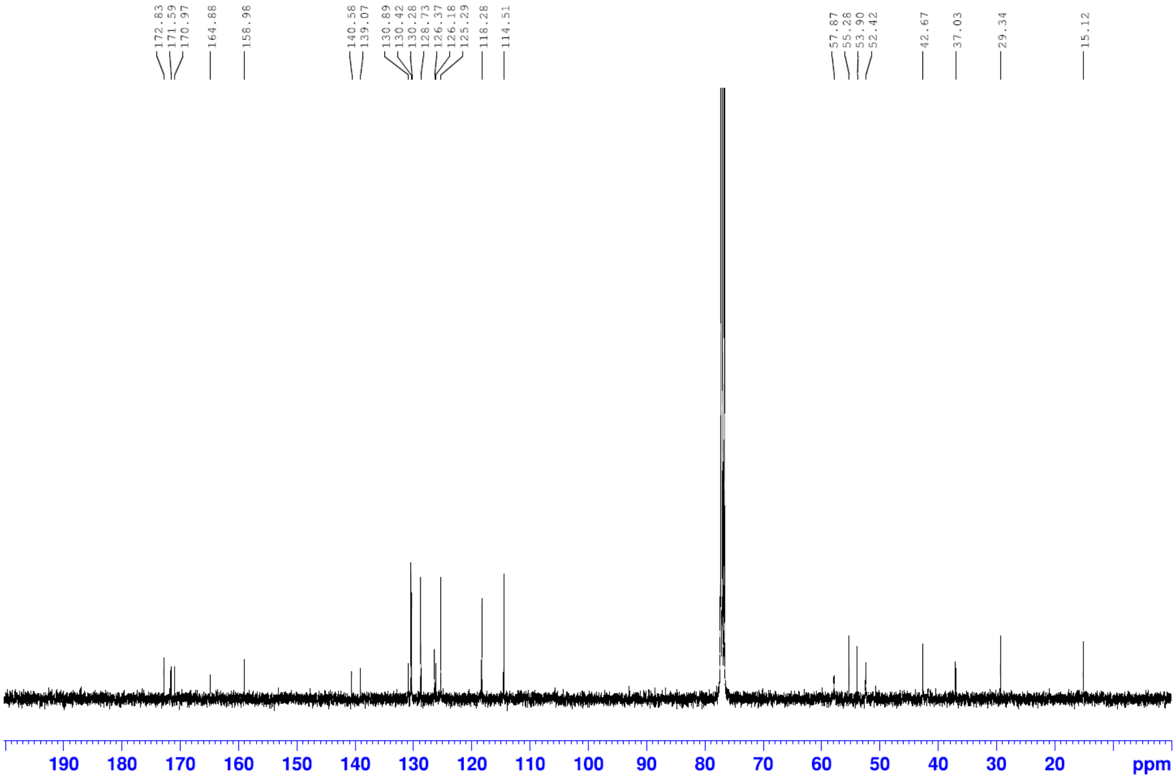

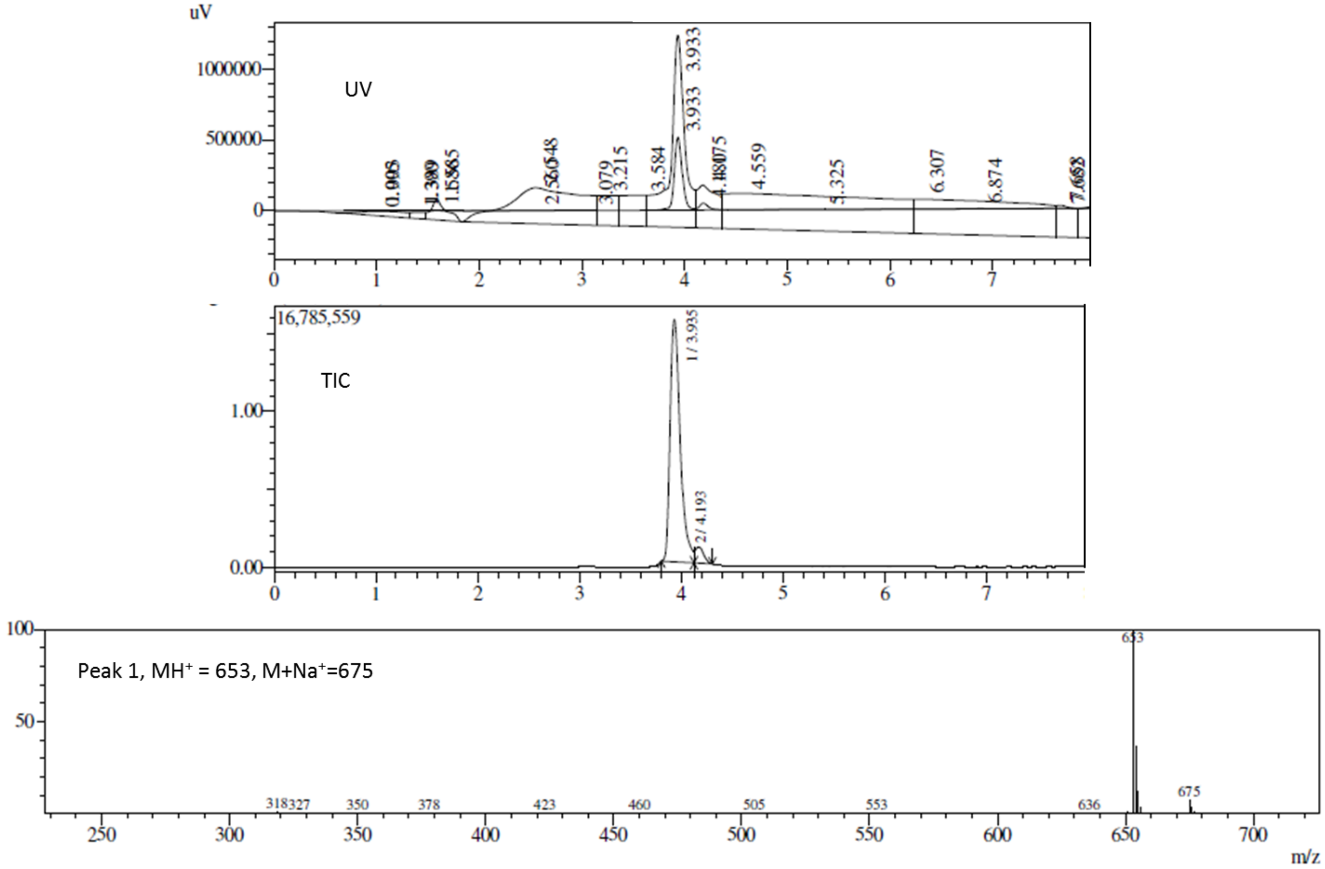
uV
UV
1000000
500000
0
3.933
3.584
4.559
5.325
6.307
6.874
16,785,559
TIC
1.00
0.00
1 / 3.935
2 / 4.193
Peak 1, MH+ = 653, M+Na+=675
100
50
653
675
636
553
505
460
423
378
350
318327
m/z

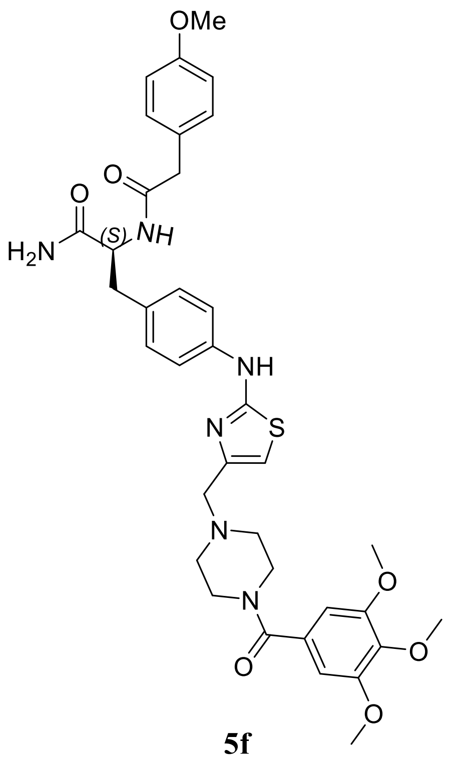

5f

$C_{36}H_{42}N_6O_7S$
Exact Mass: 702.28
Mol. Wt.: 702.82

5f (^{1}H NMR, 400 MHz, $CDCl_3$, 300 K) δ 7.19 (d, J = 9.1 Hz, 2H), 7.08 (d, J = 6.1 Hz, 2H), 7.06 (d, J = 4.6 Hz, 2H), 6.87 (d, J = 7.6 Hz, 2H), 6.64 (s, 2H), 6.55 ($s_{(broad)}$, 1H), 6.10 (d, J = 5.4 Hz, 1H), 6.07 ($s_{(broad)}$, 1H), 5.58 ($s_{(broad)}$, 1H), 4.66 (q, J = 8.4 Hz, 1H), 3.87 (s, 3H), 3.80 (s, 4H), 3.64 ($s_{(broad)}$, 2H), 3.50 ($s_{(broad)}$, 2H), 3.01 – 2.96 (m, 2H), 2.66 ($s_{(broad)}$, 6H)

5f (^{13}C NMR, 100 MHz, $CDCl_3$, 300 K) δ 172.8, 171.6, 170.1, 165.2, 159.0, 153.4, 139.0, 131.1, 130.4, 130.3, 126.2, 118.4, 114.5, 104.6, 77.2, 60.9, 56.3, 55.3, 42.7, 37.0

LCMS: $C_{36}H_{42}N_6O_7S$ (M calculated) 702.82, $C_{36}H_{43}N_6O_7S$ (MH^+ found) 703

2317 - 8 PURE, 300K, CDCL3

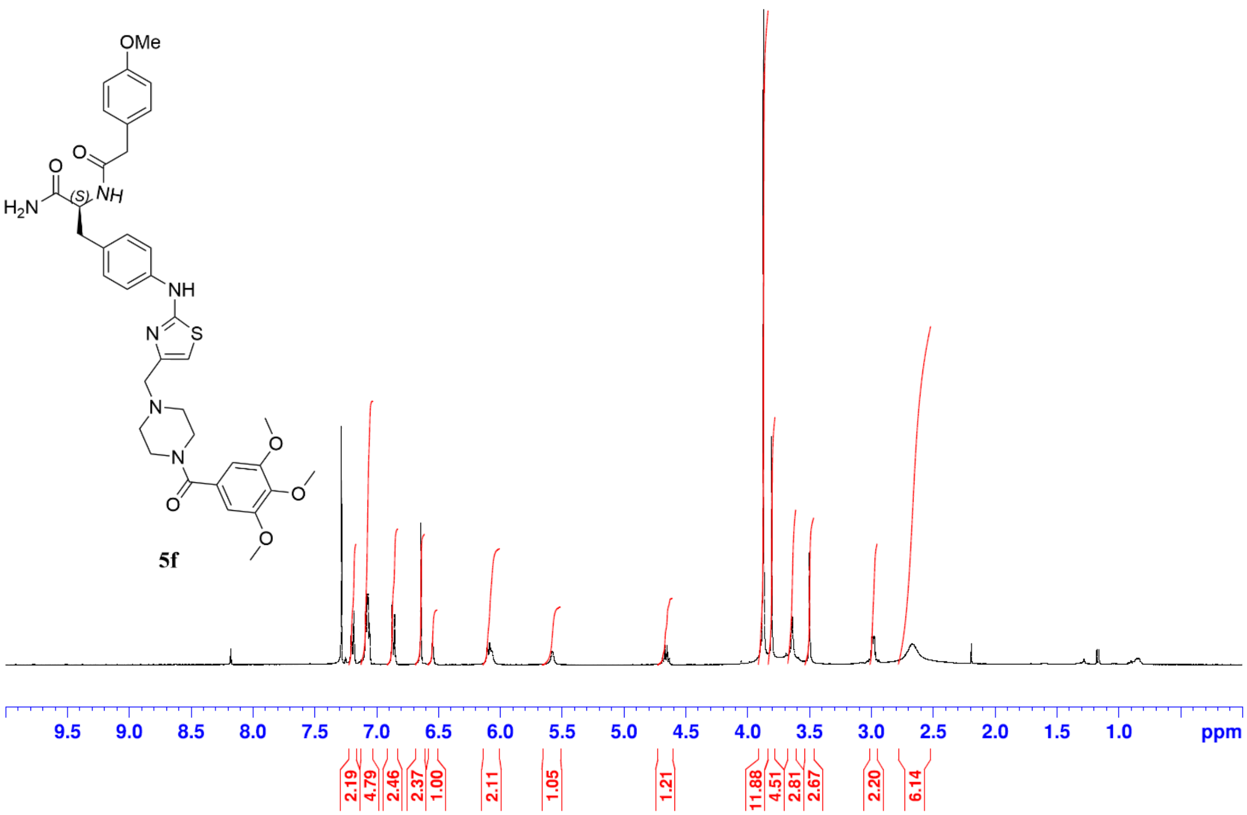

5f

317 - 8 PURE, 300K, CDCL3

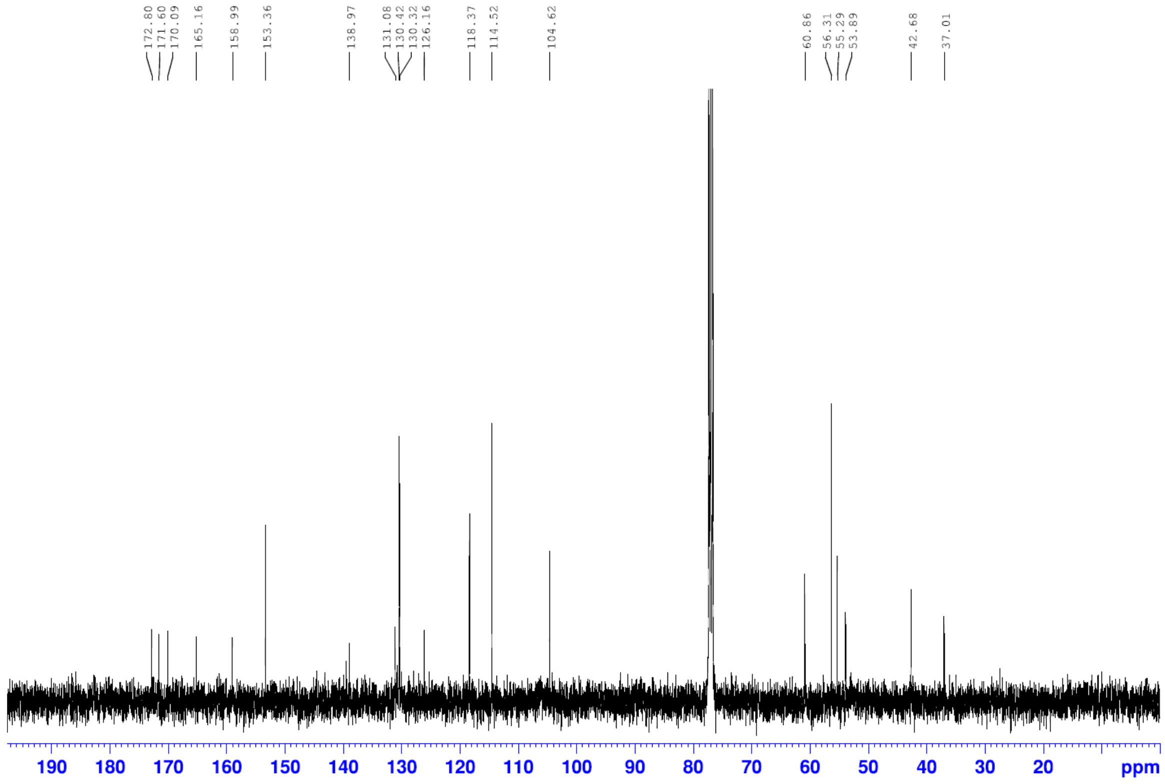

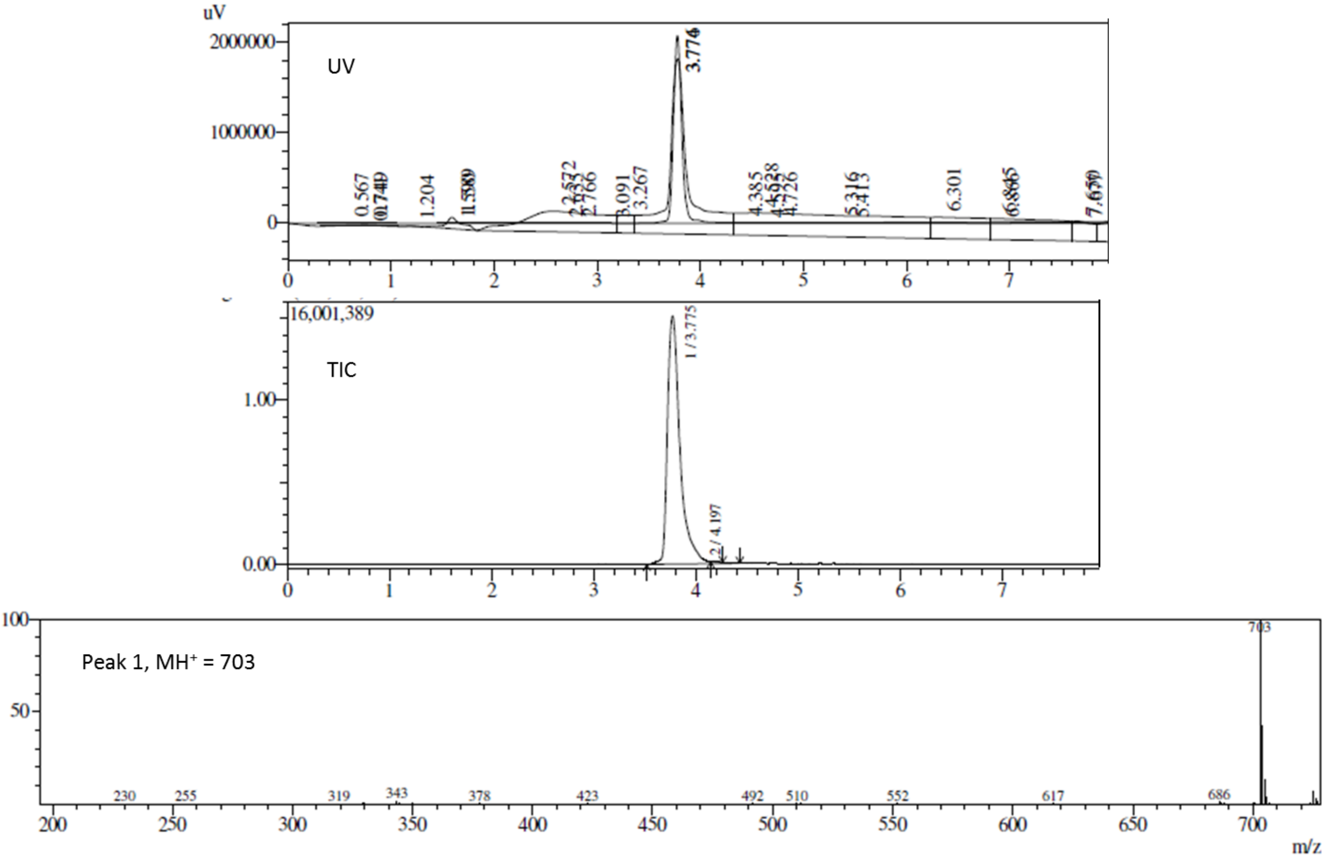
uV
UV
3.774
TIC
16,001,389
1 / 3.775
2 / 4.197
Peak 1, MH+ = 703
703
m/z

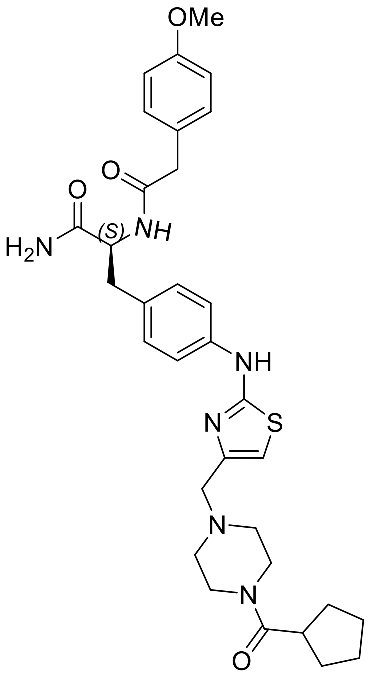

5g
$C_{32}H_{40}N_6O_4S$
Exact Mass: 604.28
Mol. Wt.: 604.76

5g (^{1}H NMR, 400 MHz, CDCl$_3$, 300 K) δ 7.19 (d, J = 9.1 Hz, 2H), 7.08 (d, J = 6.1 Hz, 2H), 7.06 (d, J = 4.6 Hz, 2H), 6.52 ($s_{(broad)}$, 1H), 6.15 (d, J = 5.4 Hz, 1H), 6.10 ($s_{(broad)}$, 1H), 5.66 ($s_{(broad)}$, 1H), 4.67 (q, J = 8.4 Hz, 1H), 3.80 (s, 3H), 3.73 ($s_{(broad)}$, 2H), 3.66 – 3.56 (m, 4H), 3.51 (s, 2H), 2.99 (d, J = 6.9 Hz, 2H), 2.88 (pent. J = 7.7 Hz, 1H), 2.64 – 2.52 (m, 4H), 1.89 – 1.69 (m, 7H), 1.64 – 1.52 (m, 3H)

5g (^{13}C NMR, 100 MHz, CDCl$_3$, 300 K) δ 174.5, 172.8, 171.6, 164.9, 159.0, 139.1, 130.9, 130.4, 130.3, 126.2, 118.3, 114.5, 77.2, 58.0, 55.3, 53.9, 52.7, 45.1, 42.7, 41.0, 37.0, 30.1, 26.0

LCMS: $C_{32}H_{40}N_6O_4S$ (M calculated) 604.76, $C_{32}H_{41}N_6O_4S$ (MH^+ found) 605

317 - 9 PURE, 300K, CDCL3

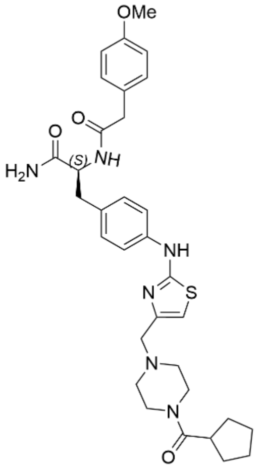

5g

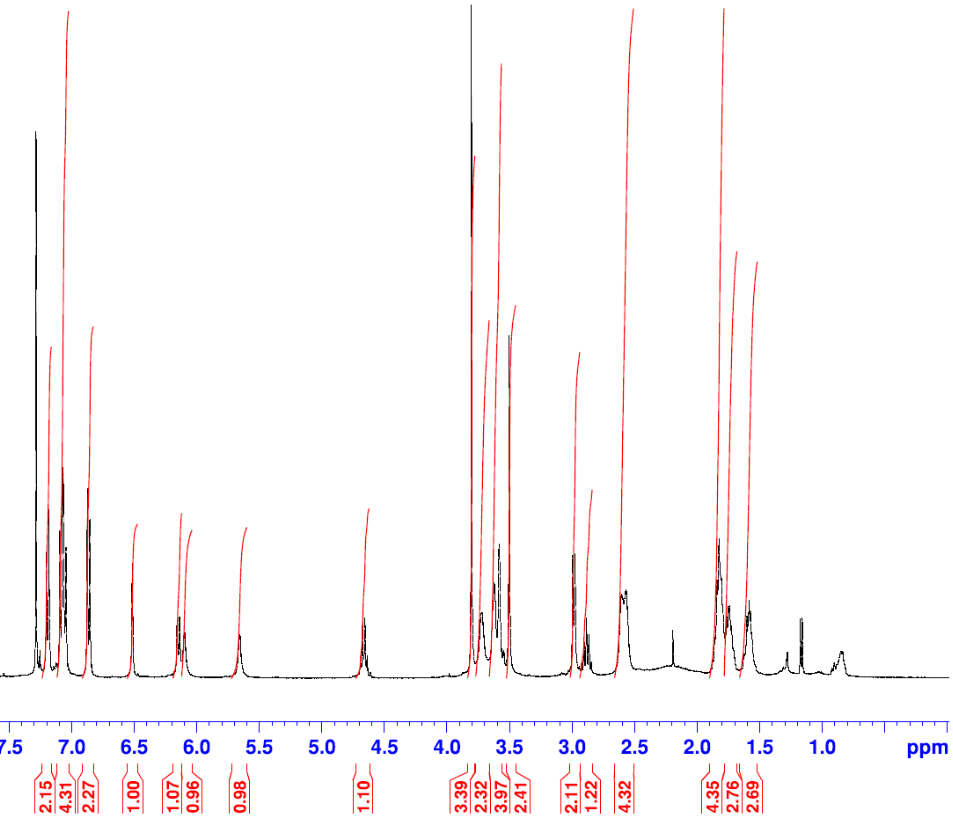

317 - 9 PURE, 300K, CDCL3

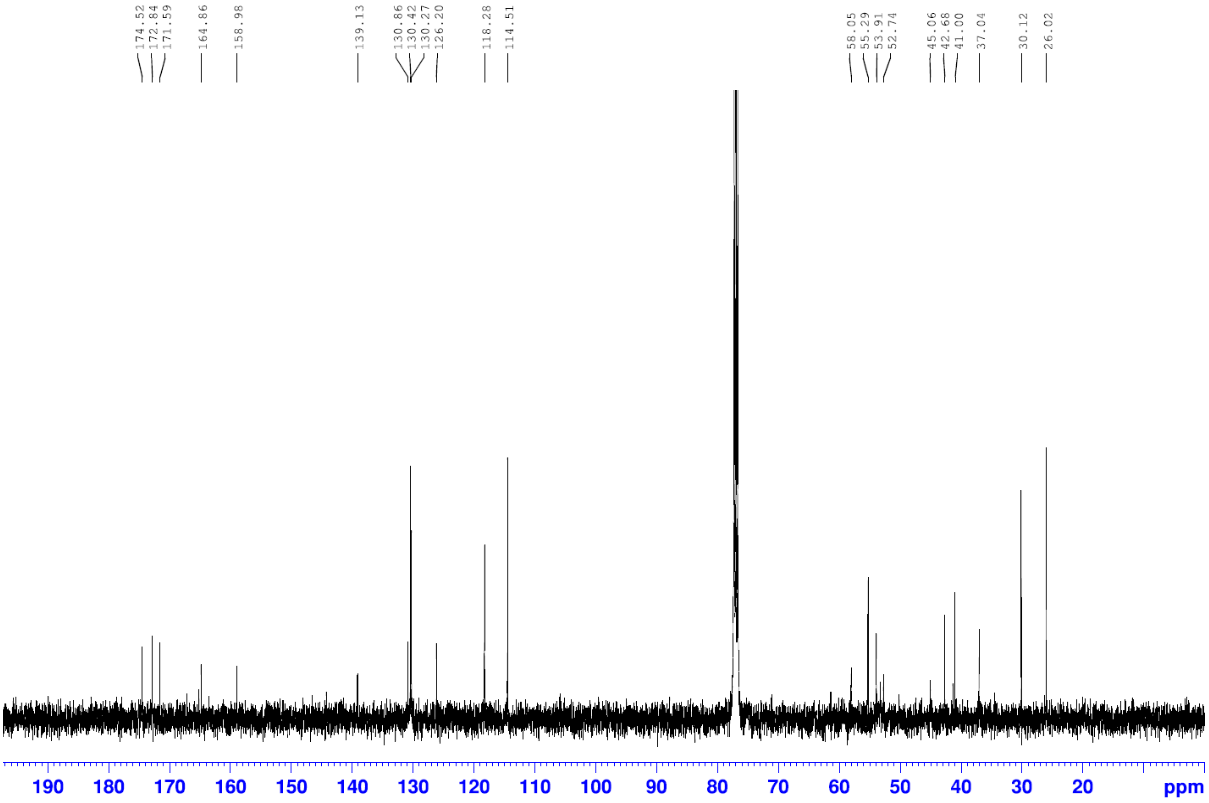

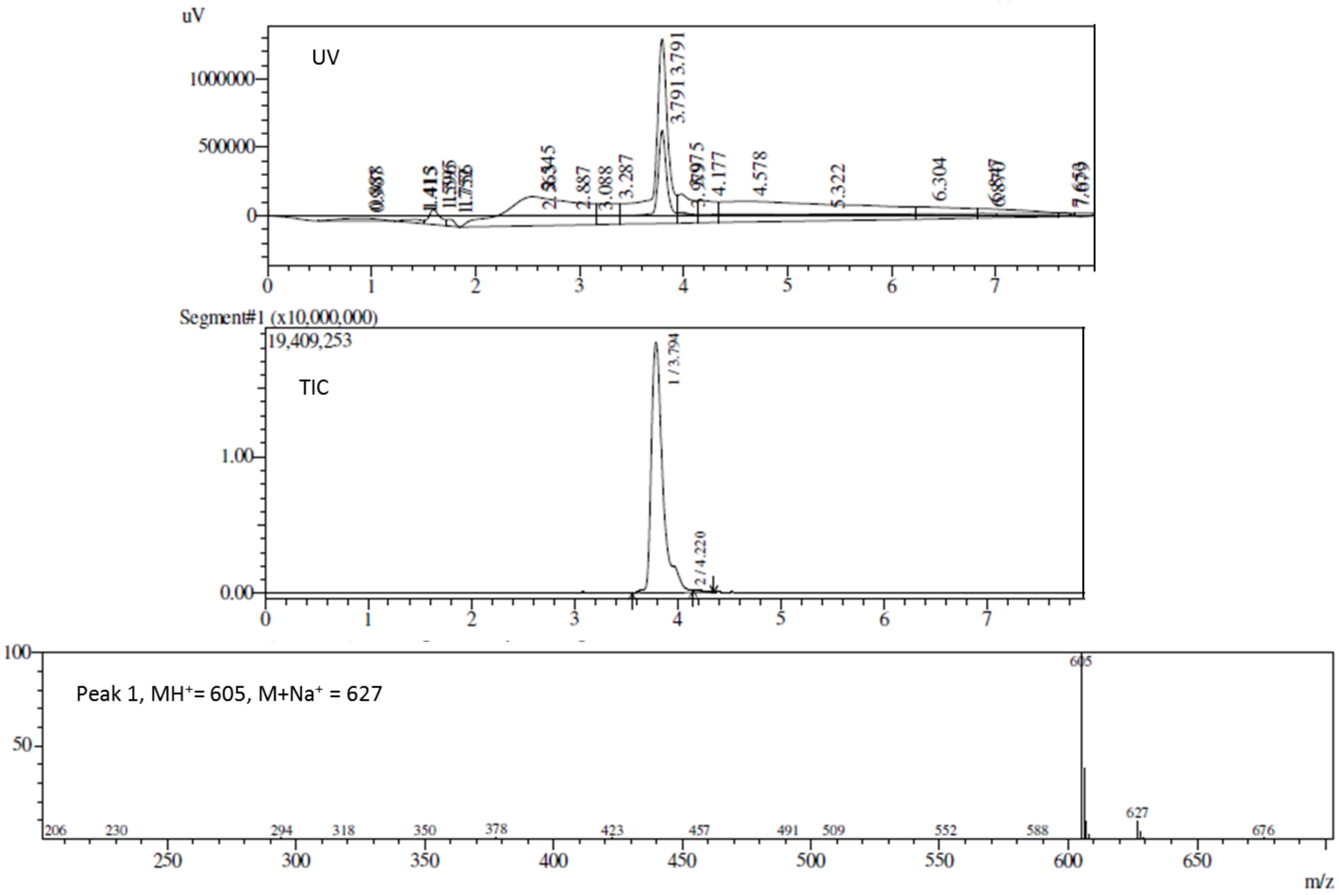

uV
UV
Segment#1 (x10,000,000)
19,409,253
TIC
1 / 3.794
2 / 4.220
Peak 1, MH⁺= 605, M+Na⁺ = 627
605
627
m/z

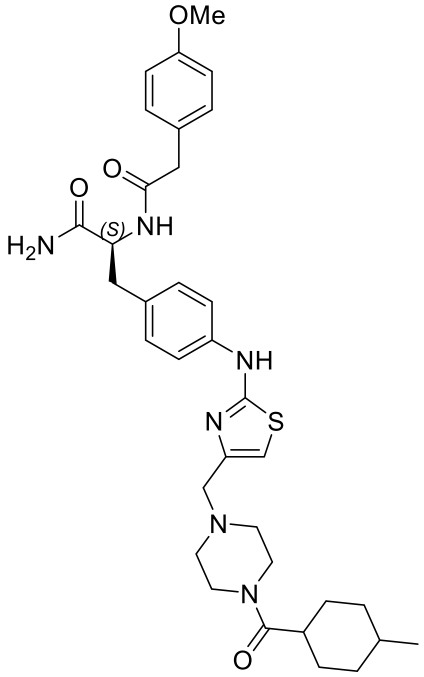

5h
$C_{34}H_{44}N_6O_4S$
Exact Mass: 632.31
Mol. Wt.: 632.82

5h (^{1}H NMR, 400 MHz, CDCl$_3$, 300 K) δ 7.18 (d, J = 9.1 Hz, 2H), 7.09 (d, J = 6.1 Hz, 2H), 7.06 (d, J = 4.6 Hz, 2H), 6.87 (d, J = 9.0 Hz, 2H), 6.46 (s, 1H), 6.12 (d, J = 5.4 Hz, 1H), 6.06 ($s_{(broad)}$, 1H), 5.64 ($s_{(broad)}$, 1H), 4.66 (q, J = 8.4 Hz, 1H), 3.81 (s, 3H), 3.67 ($s_{(broad)}$, 2H), 3.59 – 3.51 (m, 4H), 3.51 (s, 2H), 2.98 (d, J = 6.9 Hz, 2H), 2.660 – 2.46 (m, 5H), 1.85 – 1.70 (m, 4H), 1.57 – 1.46 (m, 4H), 0.99 (d, J = 5.4 Hz, 2H), 0.90 (d, J = 5.7 Hz, 2H)

5h (^{13}C NMR, 100 MHz, CDCl$_3$, 300 K) δ 174.7, 172.8, 171.6, 164.9, 159.0, 139.1, 130.8, 130.4, 130.3, 126.2, 118.3, 114.5, 105.2, 77.2, 58.2, 55.3, 53.9, 42.7, 40.2, 38.8, 37.0, 34.6, 32.1, 29.3, 28.4, 24.9, 22.6, 18.9

LCMS: $C_{34}H_{44}N_6O_4S$ (M calculated) 632.82, $C_{34}H_{45}N_6O_4S$ (MH^+ found) 633

2317 - 10 PURE, 300K, CDCL3

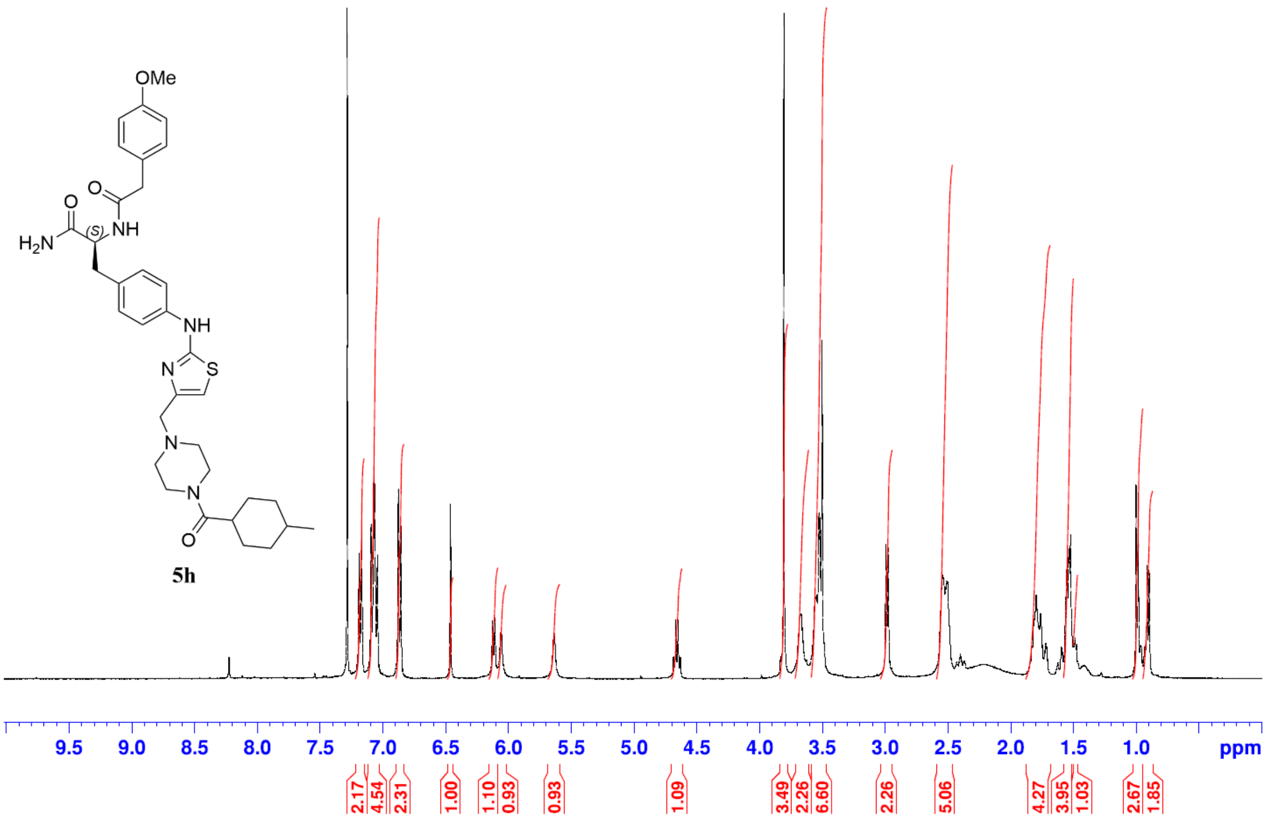

2317 - 10 PURE, 300K, CDCL3

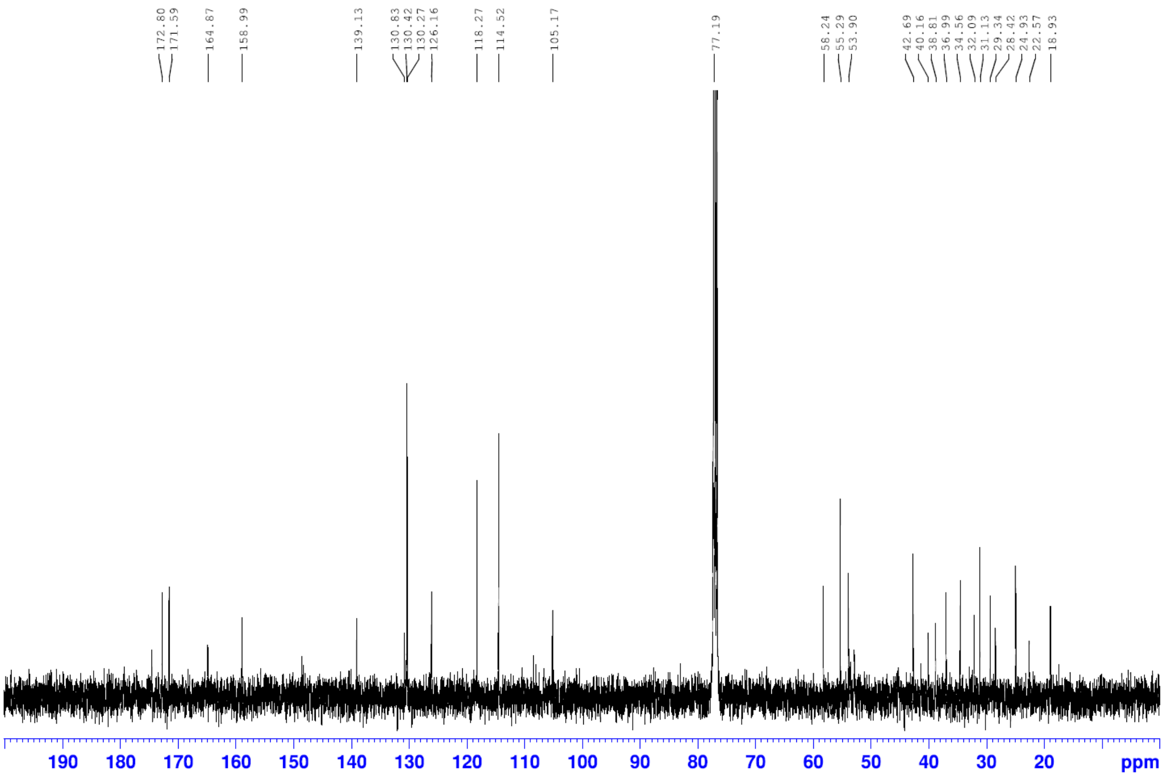

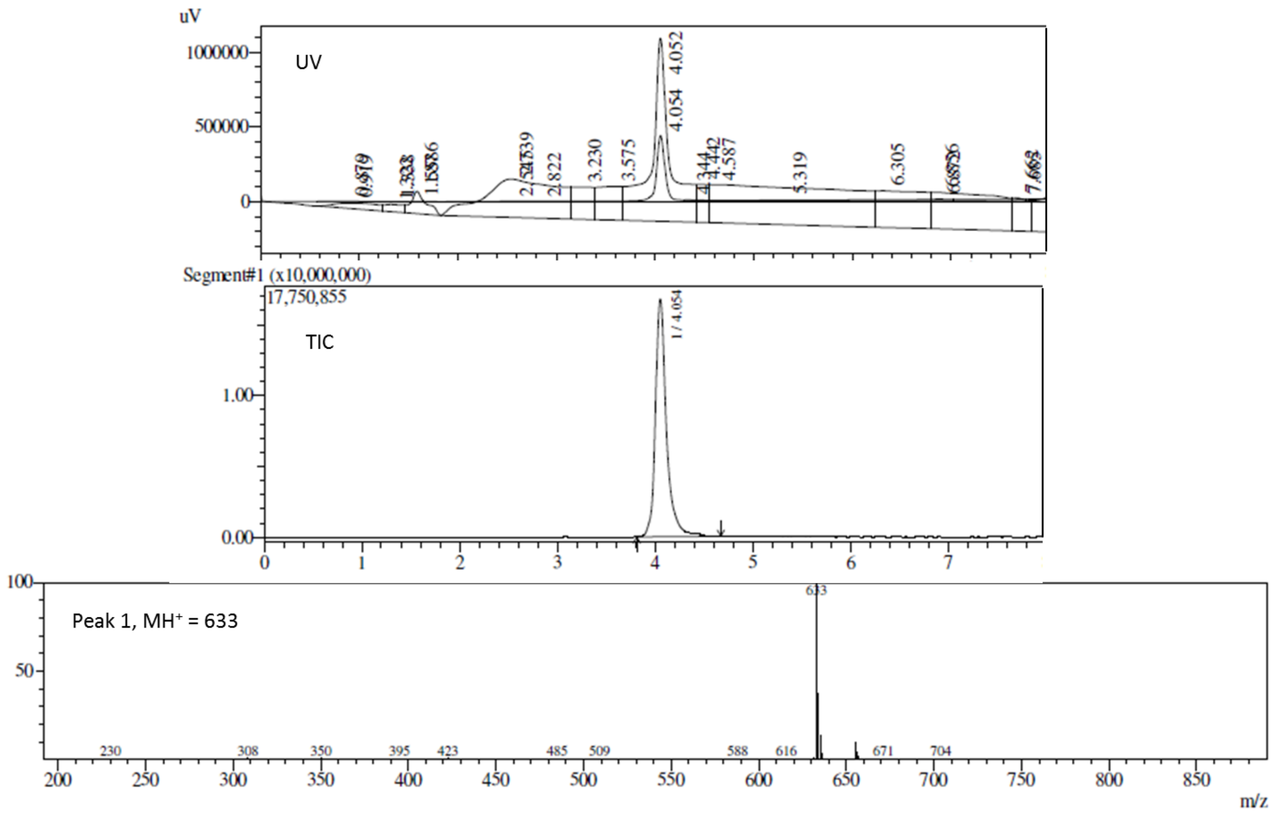
UV
TIC
Peak 1, MH+ = 633
Segment#1 (x10,000,000)
17,750,855
m/z

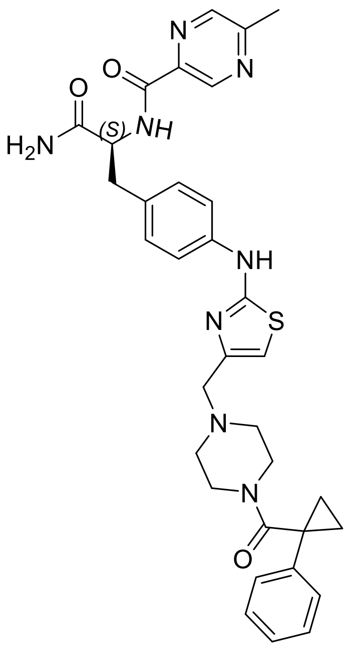

5i

$C_{33}H_{36}N_8O_3S$

Exact Mass: 624.26

Mol. Wt.: 624.76

5i (^{1}H NMR, 400 MHz, $CDCl_3$, 300 K) δ 9.24 (s, 1H), 8.41 (s, 1H), 8.30 (d, J = 12.1 Hz, 1H), 8.17 (s, 1H), 7.31 – 7.27 (m, 4H), 7.24 (d, J = 7.1 Hz, 2H), 7.20 (t, J = 7.1 Hz, 1H), 7.16 (d, J = 10.9 Hz, 2H), 6.46 (s, 1H), 6.02 ($s_{(broad)}$, 1H), 5.69 ($s_{(broad)}$, 1H), 4.88 (q, J = 8.4 Hz, 1H), 3.76 ($s_{(broad)}$, 2H), 3.57 ($s_{(broad),}$ 2H), 3.52 (s, 2H), 3.22 (d, J = 9.1 Hz, 2H), 2.67 (s, 3H), 2.59 ($s_{(broad)}$, 2H), 2.30 ($s_{(broad)}$, 2H), 1.44 – 1.40 (m, 2H), 1.22 – 1.16 (m, 2H)

5i (^{13}C NMR, 100 MHz, $CDCl_3$, 300 K) δ 172.5, 172.2, 171.0, 165.5, 163.4, 157.6, 150.2, 143.3, 142.6, 141.1, 140.5, 139.0, 131.3, 130.4, 128.8, 126.4, 125.3, 118.5, 57.2, 54.2, 52.2, 37.5, 29.3, 21.8, 15.1

LCMS: $C_{33}H_{36}N_8O_3S$ (M calculated) 624.76, $C_{33}H_{37}N_8O_3S$ (MH^+ found) 625

2317 - 11 PURE, 300K, CDCL3

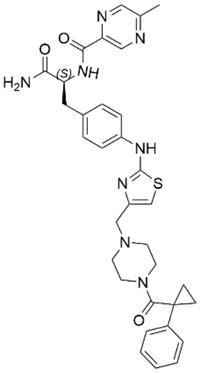

5i

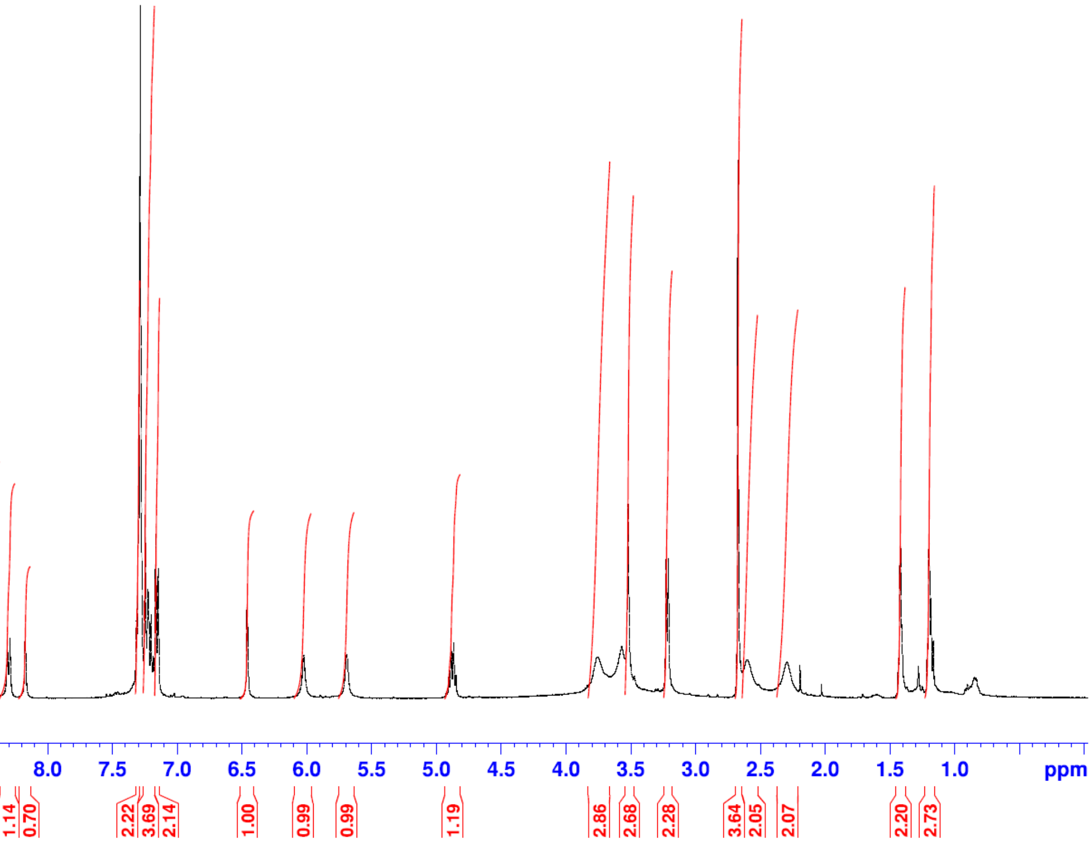

2317 - 11 PURE, 300K, CDCL3

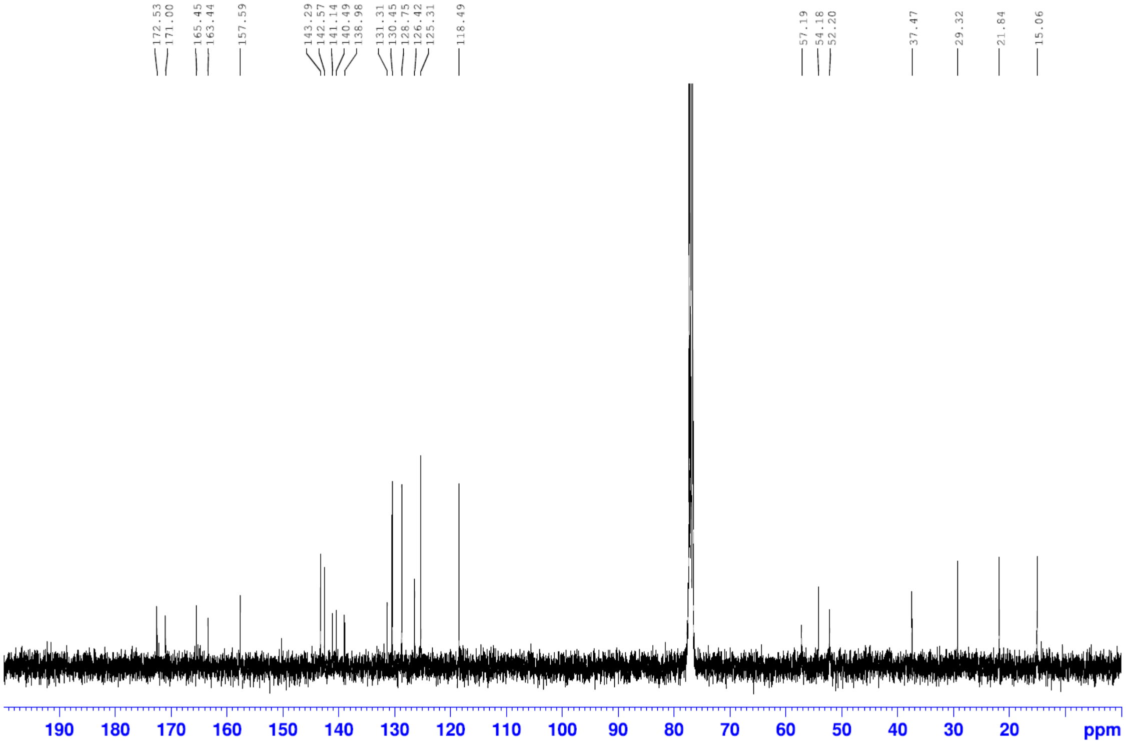

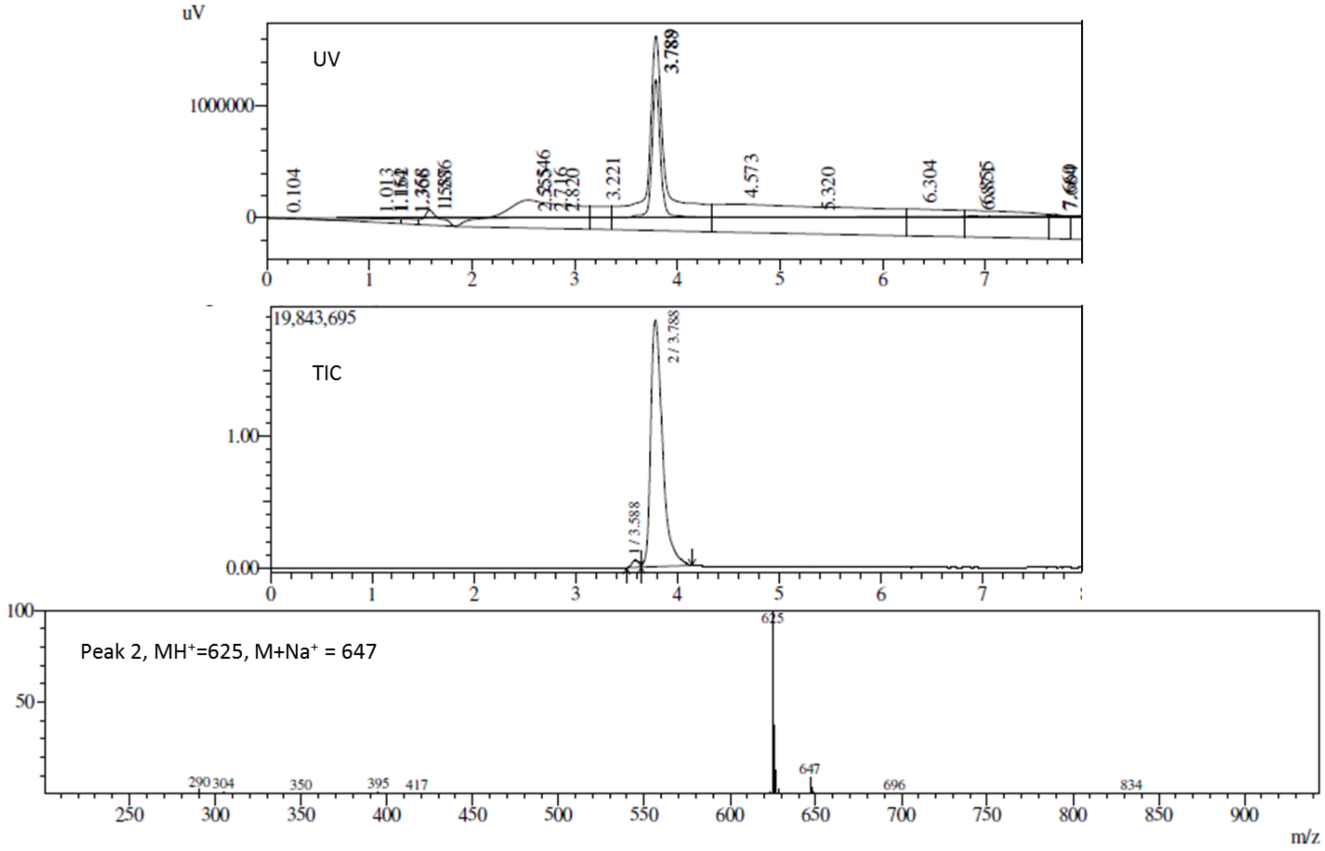
UV
uV
1000000
0
3.789
TIC
19,843,695
1.00
0.00
2 / 3.788
1 / 3.588
Peak 2, MH⁺=625, M+Na⁺ = 647
625
647
696
834
m/z

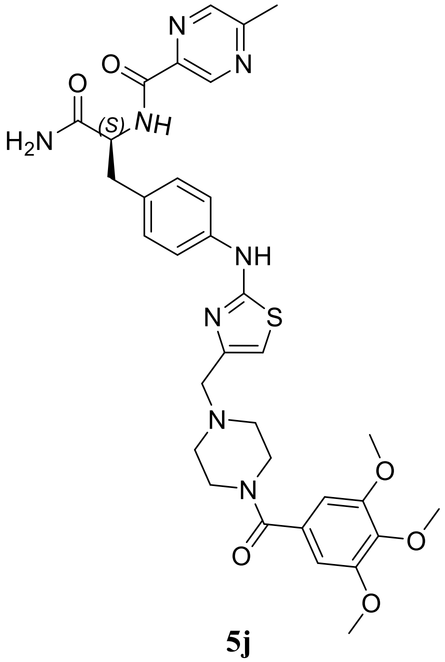

5j
$C_{33}H_{38}N_8O_6S$
Exact Mass: 674.26
Mol. Wt.: 674.77

5j (^{1}H NMR, 400 MHz, CDCl$_3$, 300 K) δ 9.15 (s, 1H), 8.32 (s, 1H), 8.22 (d, J = 8.6 Hz, 1H), 7.17 (d, J = 5.9 Hz, 4H), 6.55 (s, 2H), 6.38 (s_{broad}, 1H), 5.90 (s_{broad} , 1H), 5.53 (s_{broad} , 1H), 5.23 (s, 2H), 4.77 (q, J = 7.6 Hz, 1H), 3.79 (s, 9H), 3.78 (s, 2H), 3.46 (s, 2H), 3.12 (d, J = 6.5 HZ, 2H), 2.58 (s, 3H), 2.48 ($s_{v.\ broad}$, 4H), 1.80 ($s_{v.broad}$, 4H)

5j (^{13}C NMR, 100 MHz, CDCl$_3$, 300 K) δ 172.4, 170.1, 164.8, 163.4, 157.6, 153.3, 143.3, 142.6, 141.2, 139.4, 139.2, 131.1, 130.4, 118.3, 104.6, 60.9, 58.2, 56.3, 54.2, 53.4, 37.5, 21.8

LCMS: $C_{33}H_{38}N_8O_6S$ (M calculated) 674.77, $C_{33}H_{39}N_8O_6S$ (MH^+ found) 675

2317 - 13 PURE, 300K, CDCL3

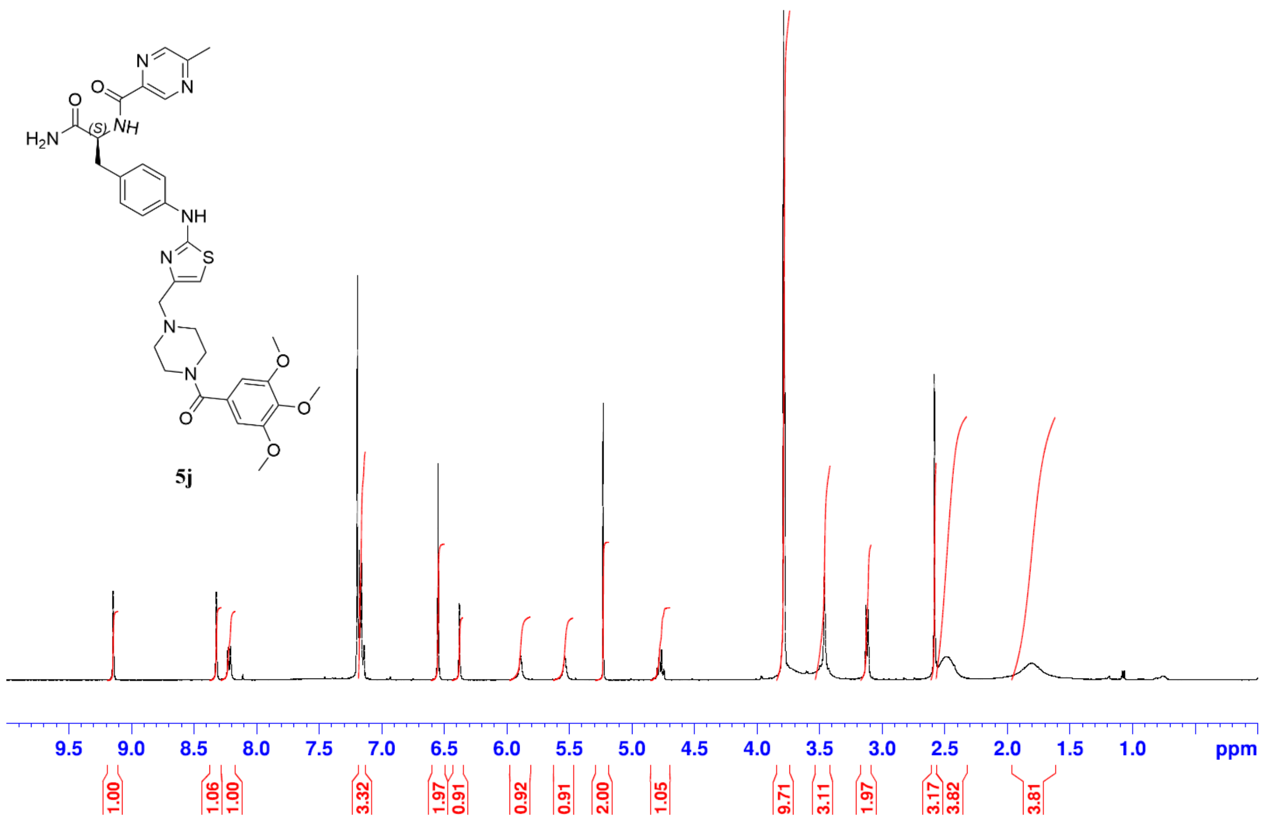

317 - 13 PURE, 300K, CDCL3

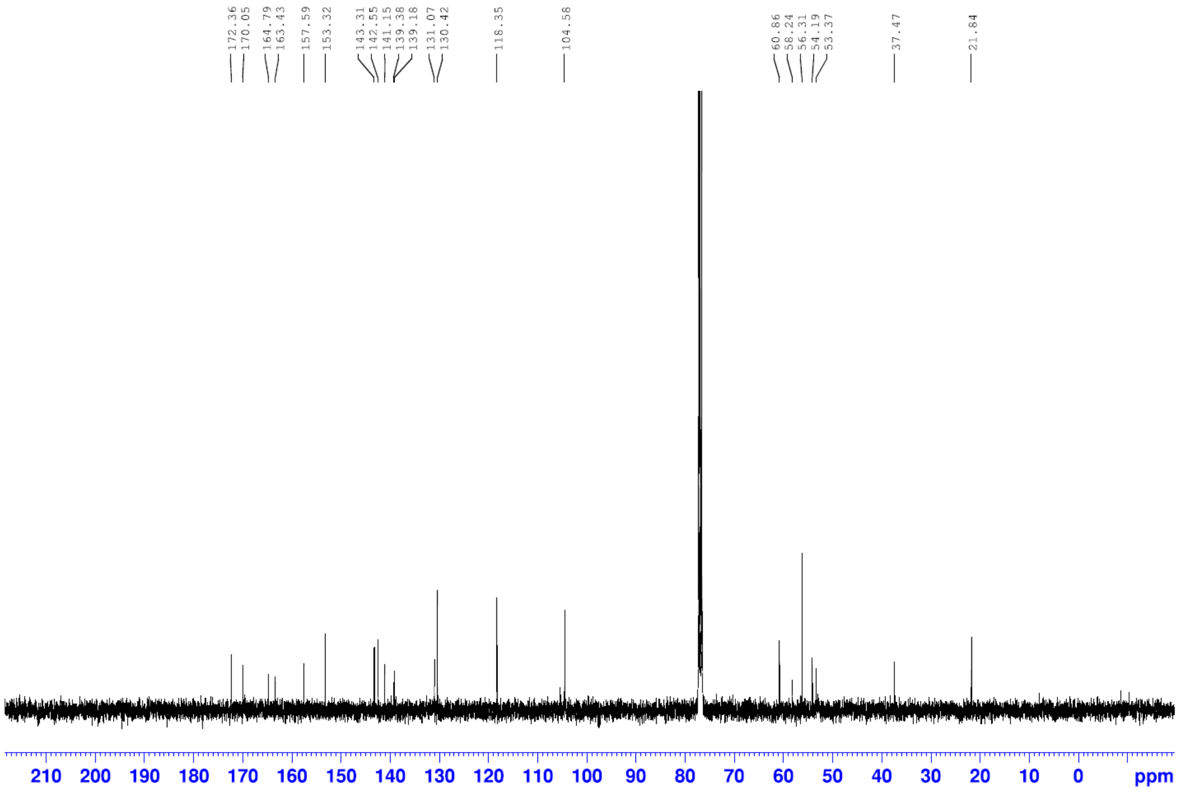

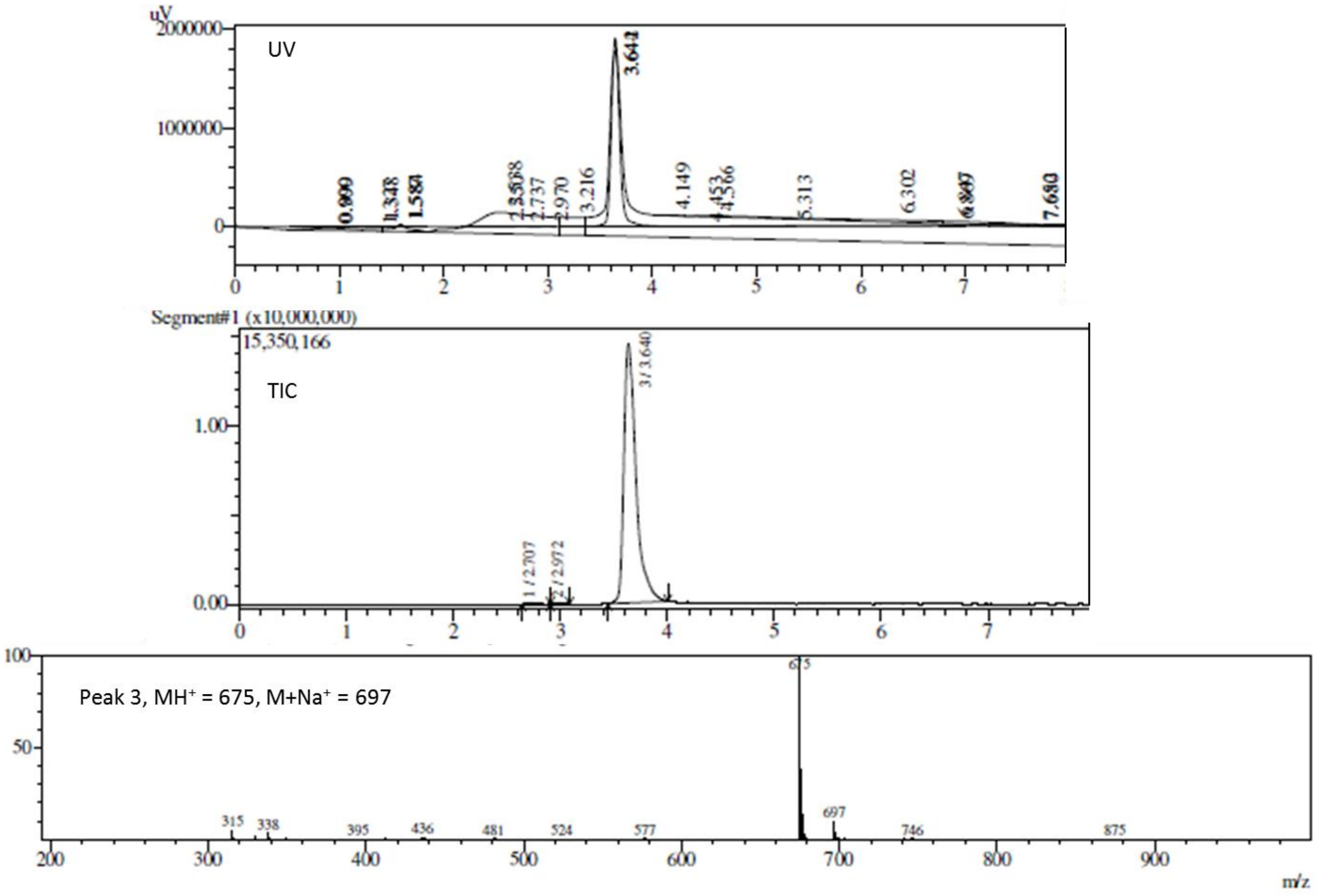

UV
TIC
Segment#1 (x10,000,000)
15,350,166
Peak 3, MH⁺ = 675, M+Na⁺ = 697
m/z

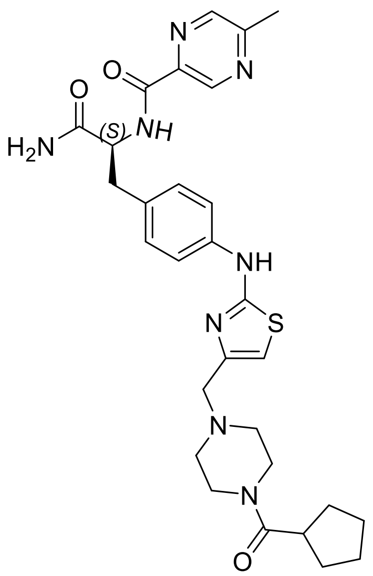

5k
$C_{29}H_{36}N_8O_3S$
Exact Mass: 576.26
Mol. Wt.: 576.71

5k (^{1}H NMR, 400 MHz, CDCl$_3$, 300 K) δ 9.24 (s, 1H), 8.42 (s, 1H), 8.32 (d, J = 8.3 Hz, 1H), 7.26 (d, J = 6.0 Hz, 4H), 6.47 (s, 1H), 5.97 (s_{broad}, 1H), 5.64 (s_{broad} , 1H), 5. 32 (s, 2H), 4.87 (q, J = 6.8 Hz, 1H), 3.73-3.66 (m, 2H), 3.62-3.57 (m, 2H), 3.53 (s, 2H), 3.24-3.19 (m, 2H), 2.89 (pent., J = 7.6 Hz, 1H), 2.67 (s, 3H), 2.57-2.49 (m, 4H), 1.97 ($s_{v. broad}$, 2H), 1.86-1.70 (m, 6H), 1.63 – 1.53 (m, 2H)

5k (^{13}C NMR, 100 MHz, CDCl$_3$, 300 K) δ 174.5, 172.3, 164.8, 163.4, 157.6, 143.3, 142.6, 141.2, 139.2, 131.0, 130.4, 118.3, 58.2, 54.2, 53.4, 53.4, 52.9, 41.0, 37.5, 30.1, 26.0, 21.8

LCMS: $C_{29}H_{36}N_8O_3S$ (M calculated) 576.71, $C_{29}H_{37}N_8O_3S$ (MH^+ found) 577

2317 - 14 PURE, 300K, CDCL3

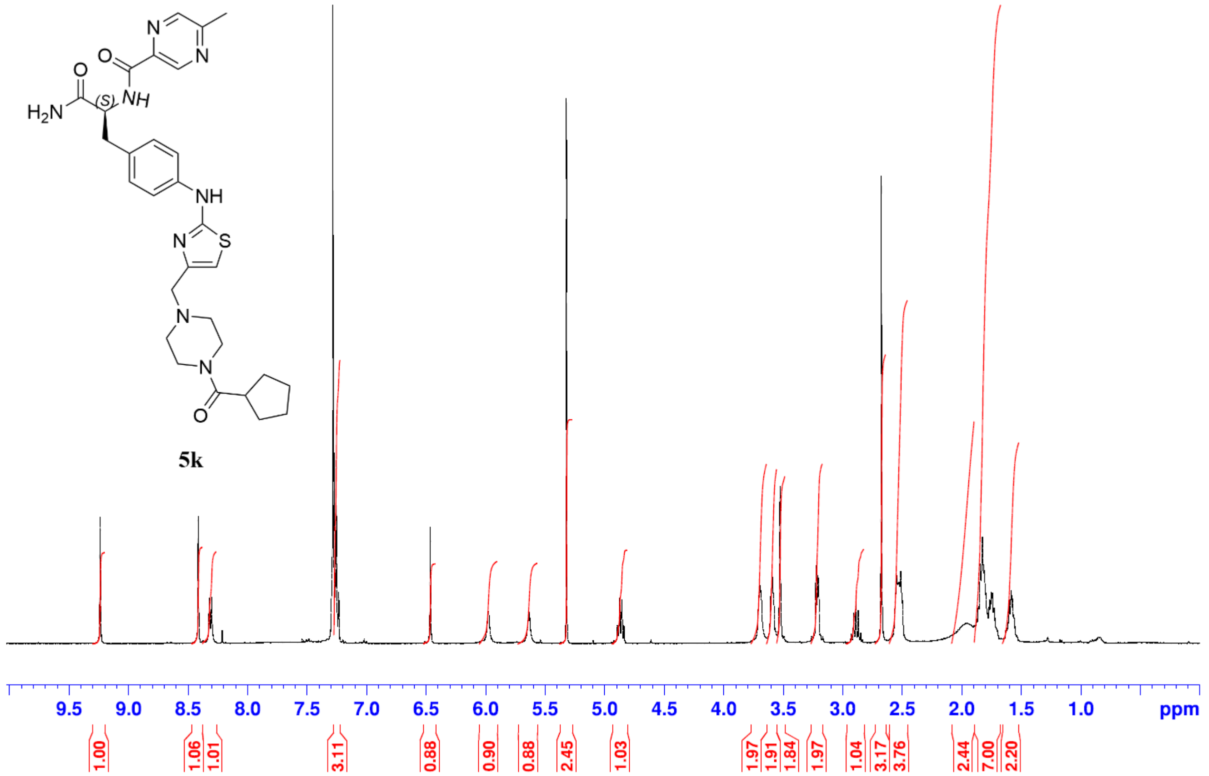

317 - 14 PURE, 300K, CDCL3

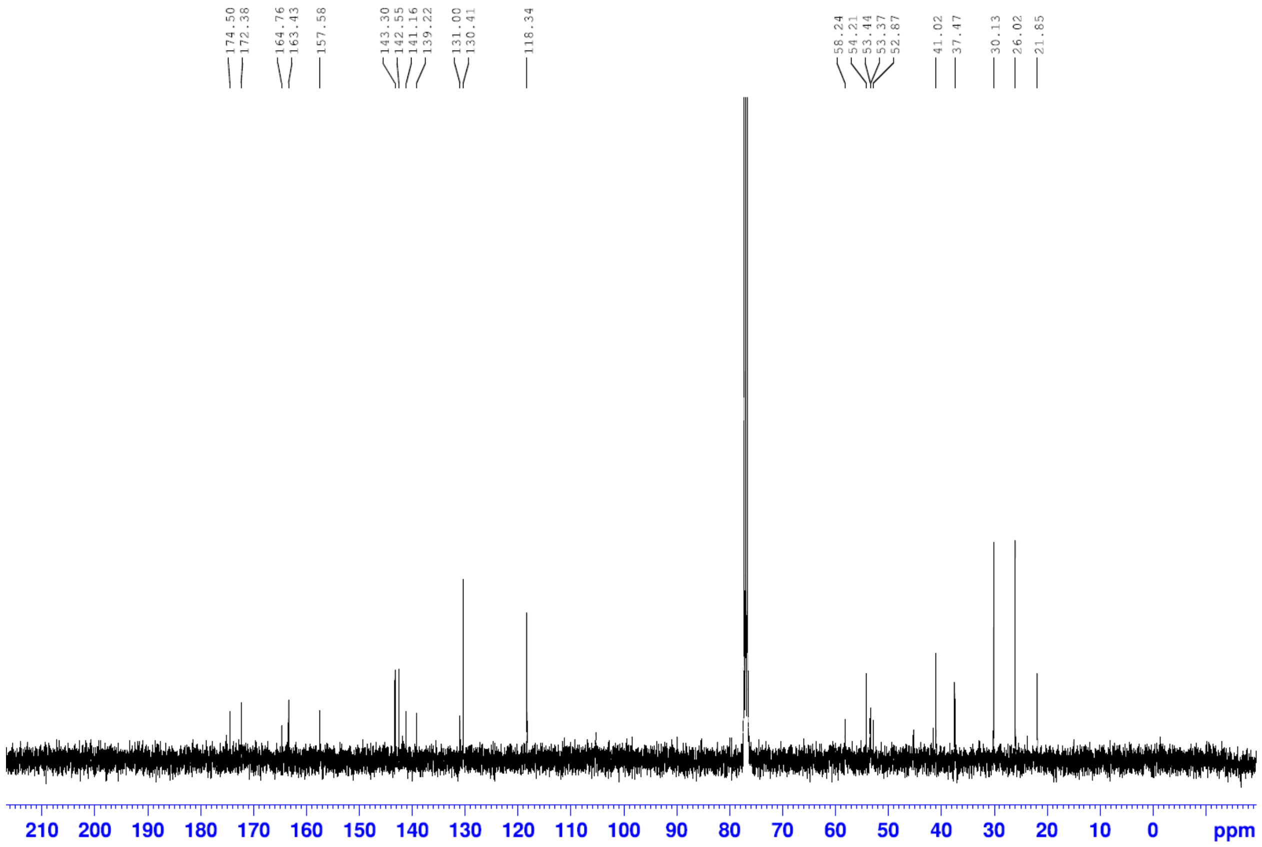

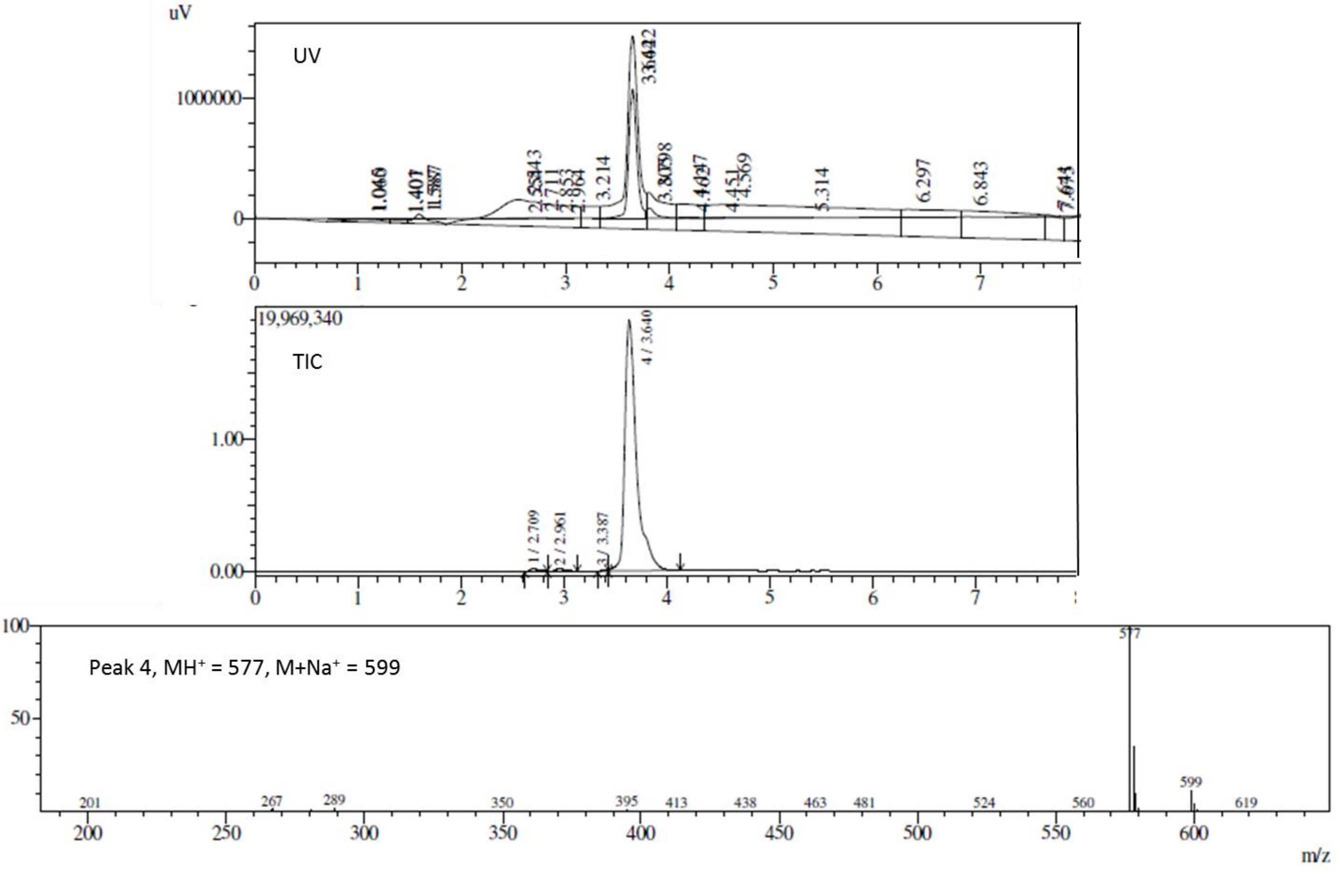
uV
UV
1000000
0
TIC
19,969,340
1.00
0.00
4 / 3.640
Peak 4, MH+ = 577, M+Na+ = 599
m/z

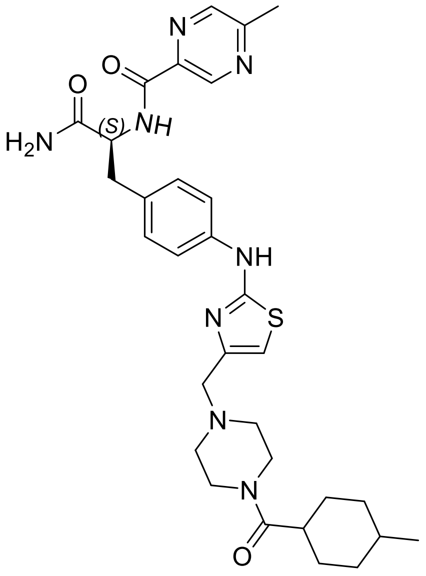

5l

$C_{31}H_{40}N_8O_3S$
Exact Mass: 604.29
Mol. Wt.: 604.77

5l (^{1}H NMR, 400 MHz, CDCl$_3$, 300 K) δ 9.14 (s, 1H), 8.32 (s, 1H), 8.23 (d, J = 8.4 Hz, 1H), 7.17 (d, J = 4.9 Hz, 4H), 6.37 (s, 2H), 5.91 (s_{broad}, 1H), 5.59 (s_{broad} , 1H), 5.53 (s_{broad} , 1H), 5.23 (s, 2H), 4.78 (q, J = 8.3 Hz, 1H), 3.59 (s_{broad}, 2H), 3.47 (s_{broad} , 2H), 3.43 (s, 2H), 3.14-3.10 (m, 2H), 2.58 (s, 3H), 2.49-2.38 (m, 2H), 1.86 ($s_{v.\ broad}$, 4H), 1.76 – 1.70 (m, 4H), 1.51-1.37 (m, 4H), 0.90 (d, J = 6.8 Hz, 2H), 0.81 (d, J = 6.6 Hz, 2H)

5l (^{13}C NMR, 100 MHz, CDCl$_3$, 300 K) δ 174.5, 172.4, 164.7, 163.4, 157.6, 143.3, 142.6, 141.2, 139.2, 131.0, 130.4, 118.3, 105.4, 58.3, 54.2, 53.6, 41.4, 40.2, 38.8, 37.5, 34.6, 32.1, 31.1, 29.3, 28.4, 24.9, 22.6, 21.8, 18.9

LCMS: $C_{31}H_{40}N_8O_3S$ (M calculated) 604.29, $C_{31}H_{41}N_8O_3S$ (MH^+ found) 605

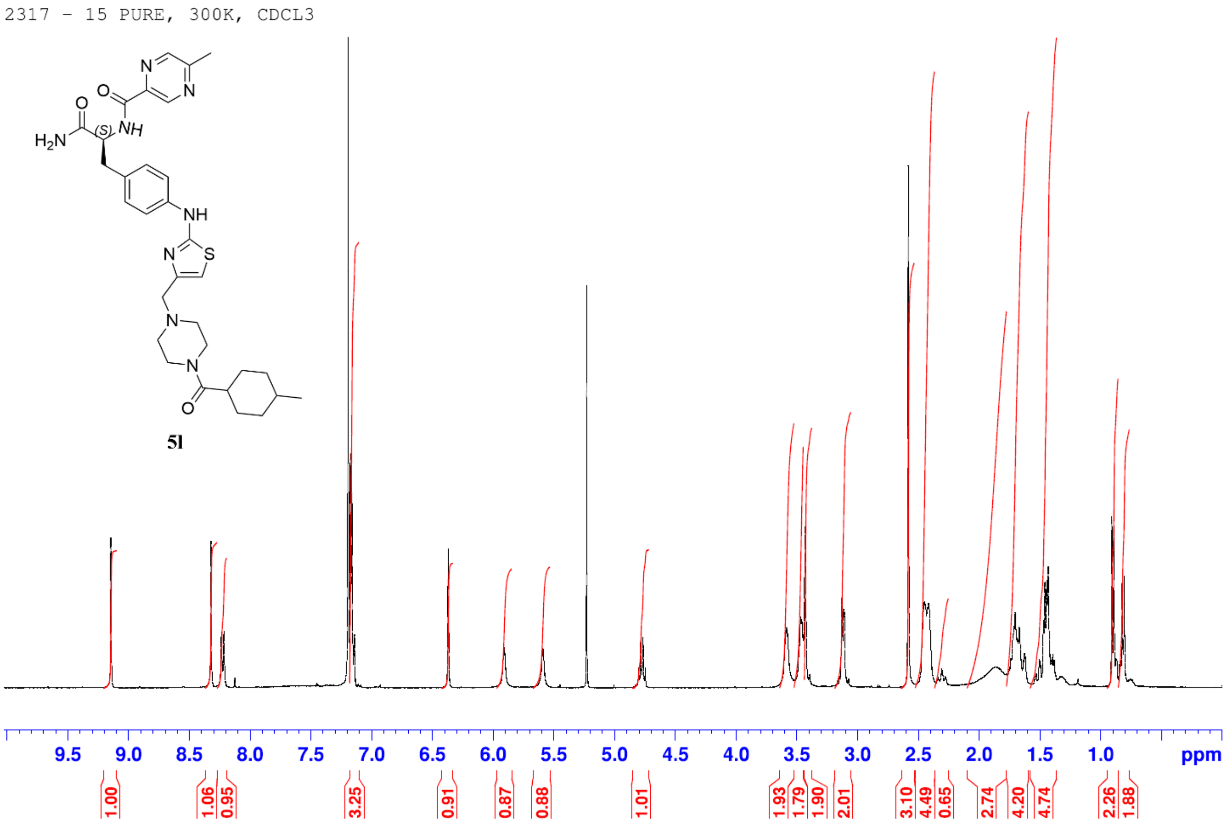
2317 - 15 PURE, 300K, CDCL3
5l
9.5 9.0 8.5 8.0 7.5 7.0 6.5 6.0 5.5 5.0 4.5 4.0 3.5 3.0 2.5 2.0 1.5 1.0 ppm
1.00 1.06 0.95 3.25 0.91 0.87 0.88 1.01 1.93 1.79 1.90 2.01 3.10 4.49 0.65 2.74 4.20 4.74 2.26 1.88

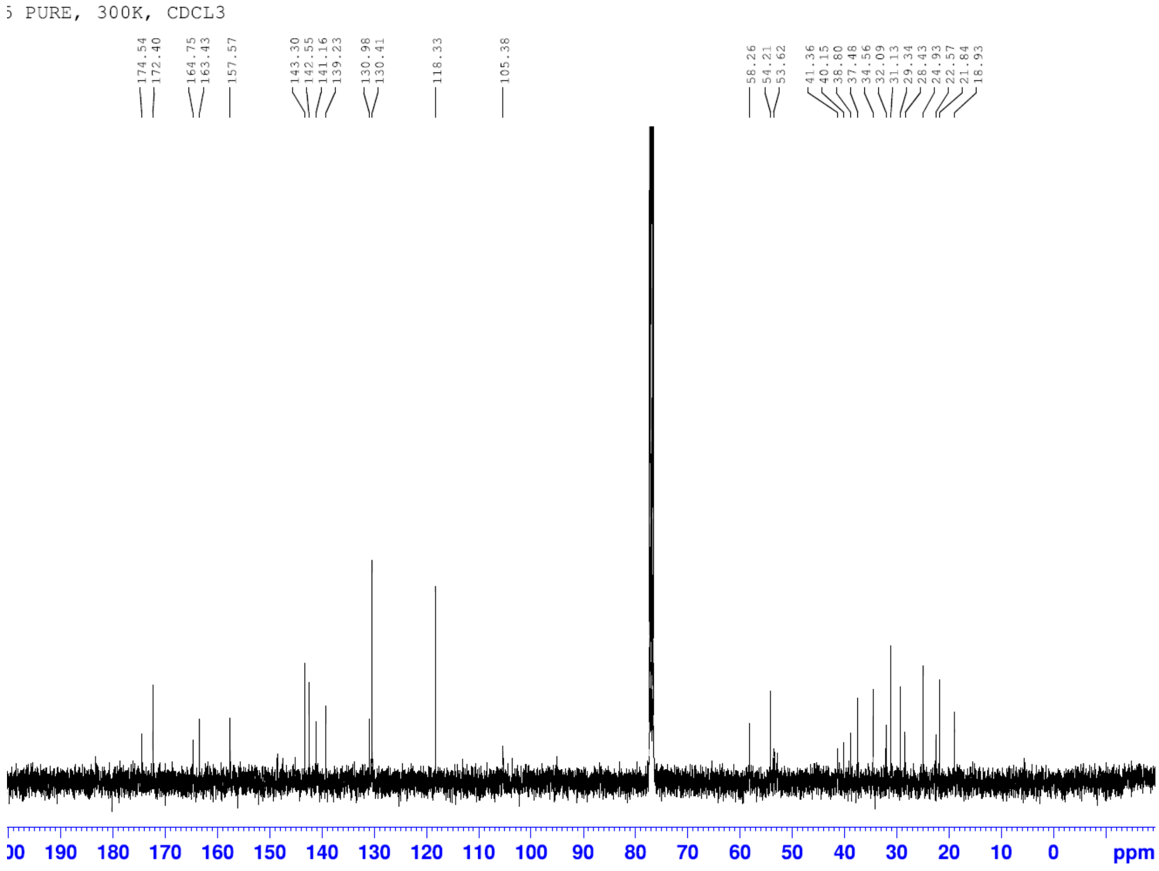
5 PURE, 300K, CDCL3
174.54 172.40 164.75 163.43 157.57 143.30 142.55 141.16 139.23 130.98 130.41 118.33 105.38
58.26 54.21 53.62 41.36 40.15 38.80 37.48 34.56 32.09 31.13 29.34 28.43 24.93 22.57 21.84 18.93
00 190 180 170 160 150 140 130 120 110 100 90 80 70 60 50 40 30 20 10 0 ppm

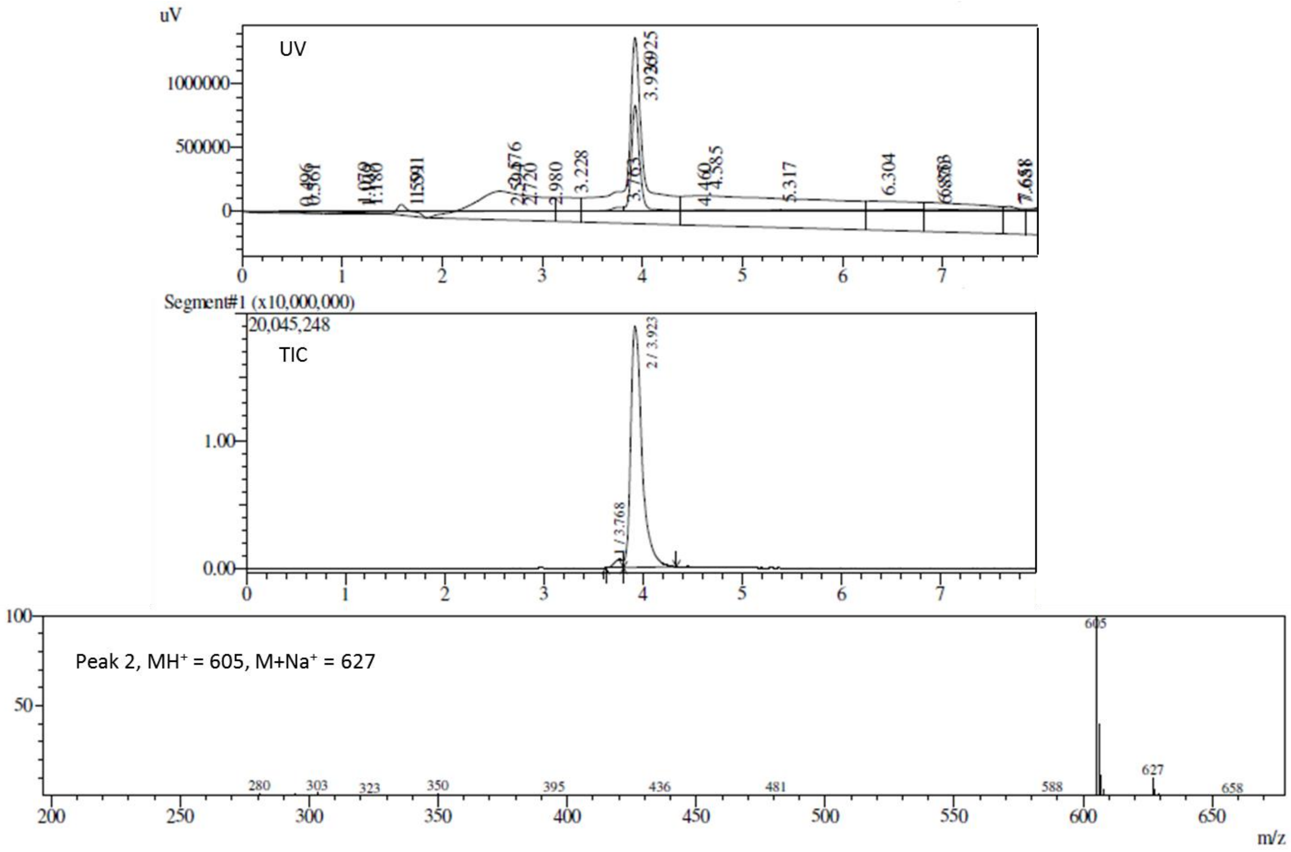
UV
TIC
Segment#1 (x10,000,000)
20,045,248
2 / 3.923
1 / 3.768
Peak 2, MH+ = 605, M+Na+ = 627
m/z

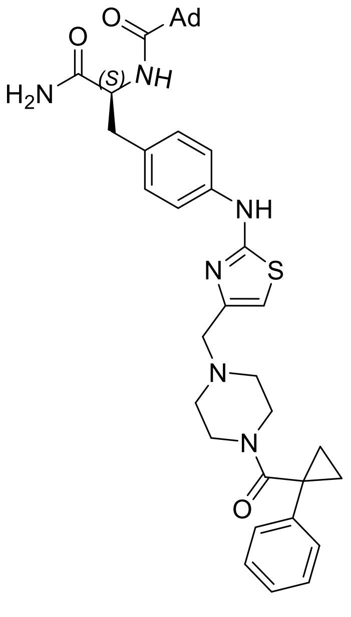

5m
$C_{38}H_{46}N_6O_3S$
Exact Mass: 666.34
Mol. Wt.: 666.88

5m (^{1}H NMR, 400 MHz, $CDCl_3$, 300 K) δ 7.24 – 7.05 (m, 10H), 6.32 (s, 1H), 6.17 (d, J = 9.1 Hz, 1H), 5.87 (S_{broad}, 1H), 5.46 (s_{broad} , 1H), 5.23 (s, 2H), 4.56 (q, J = 7.5 Hz, 1H), 3.62 (s_{broad}, 2H), 3.43 (s_{broad}, 2H), 3.34 (s, 2H), 2.97 (s, J = 7.1 Hz, 2H), 2.40 ($s_{v.\ broad}$, 2H), 2.13 ($s_{v.\ broad}$, 2H), 1.95 (s_{broad}, 2H), 1.72 (m, 6H), 1.62 (q, J = 15.5 Hz, 8H), 1.35-1.32 (m, 2H), 1.12-1.08 (m, 2H)

5m (^{13}C NMR, 100 MHz, $CDCl_3$, 300 K) δ 178.1, 173.1, 170.9, 164.9, 140.7, 139.2, 131.3, 130.4, 128.7, 126.3, 125.3, 118.4, 105.1

LCMS: $C_{38}H_{46}N_6O_3S$ (M calculated) 666.88, $C_{38}H_{47}N_6O_3S$ (MH^+ found) 667

2317 - 16 PURE, 300K, CDCL3

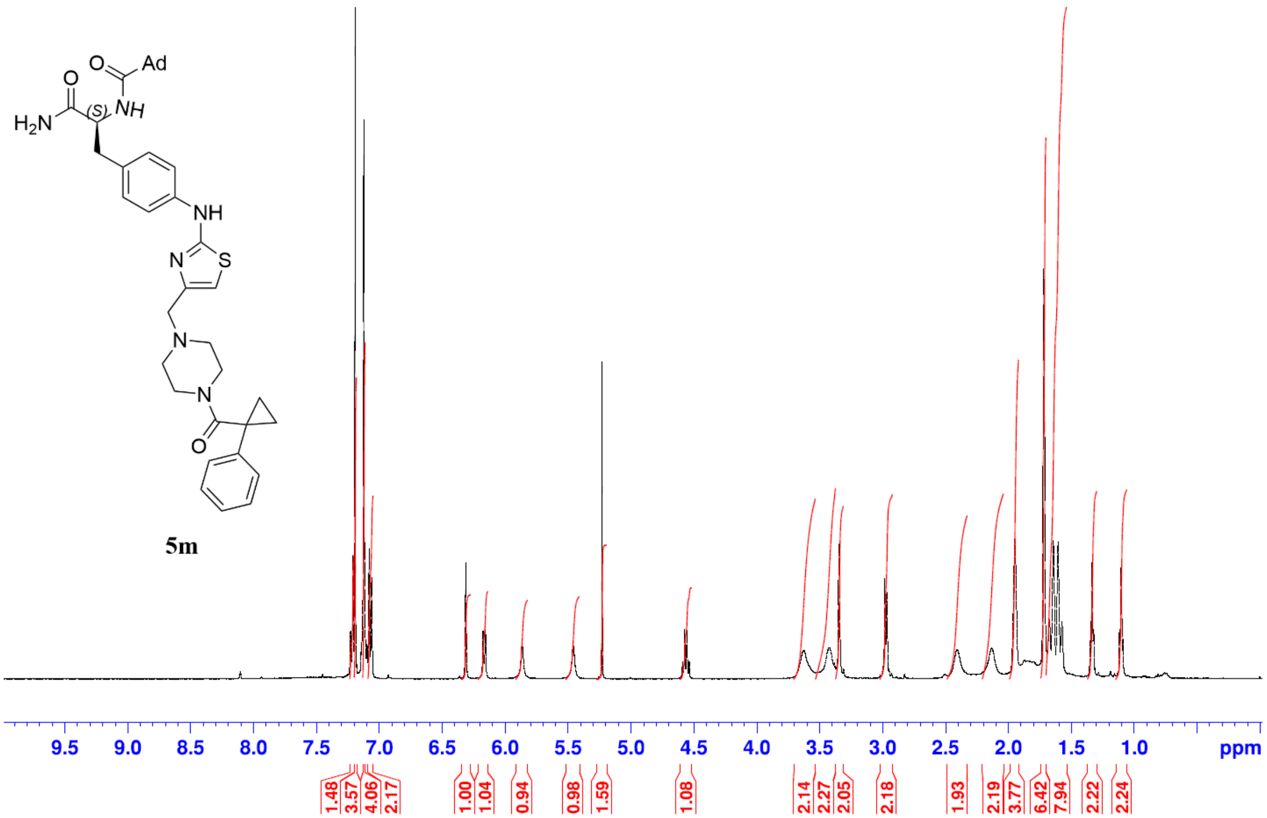

2317 - 16 PURE, 300K, CDCL3

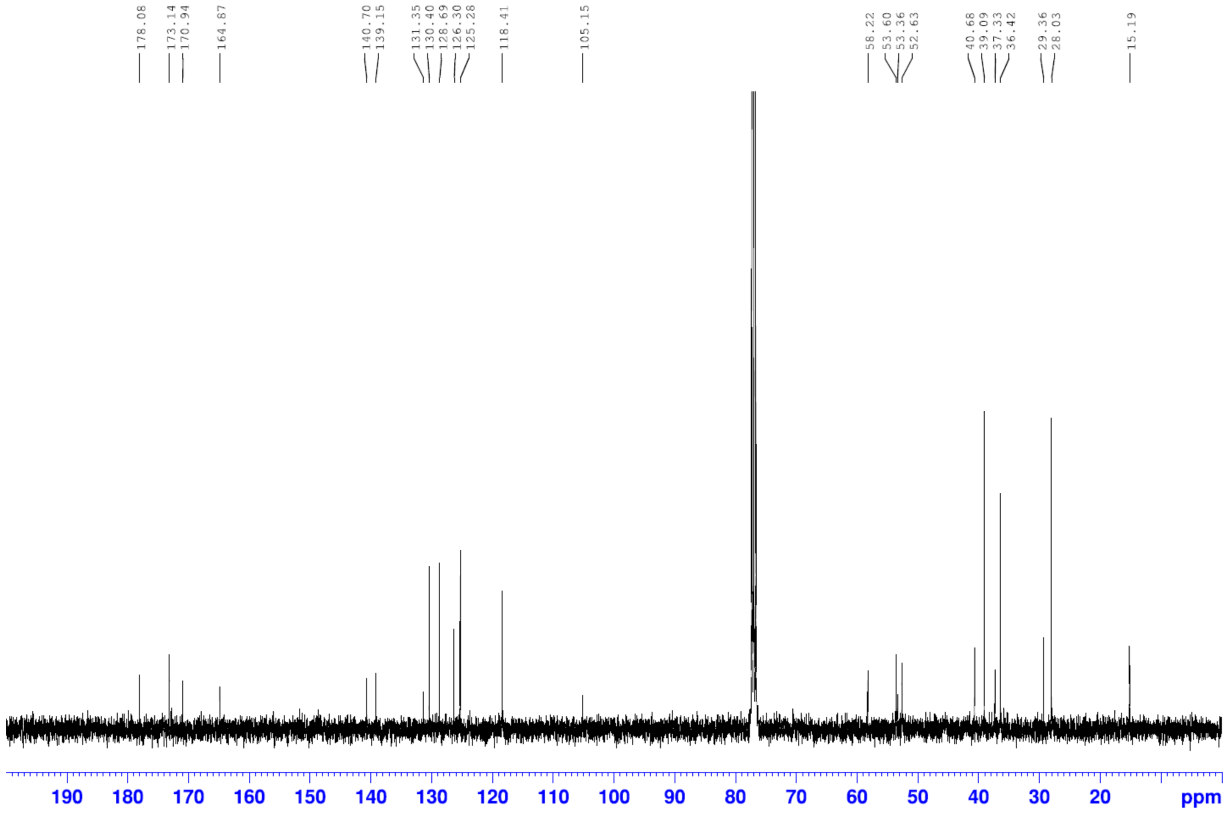

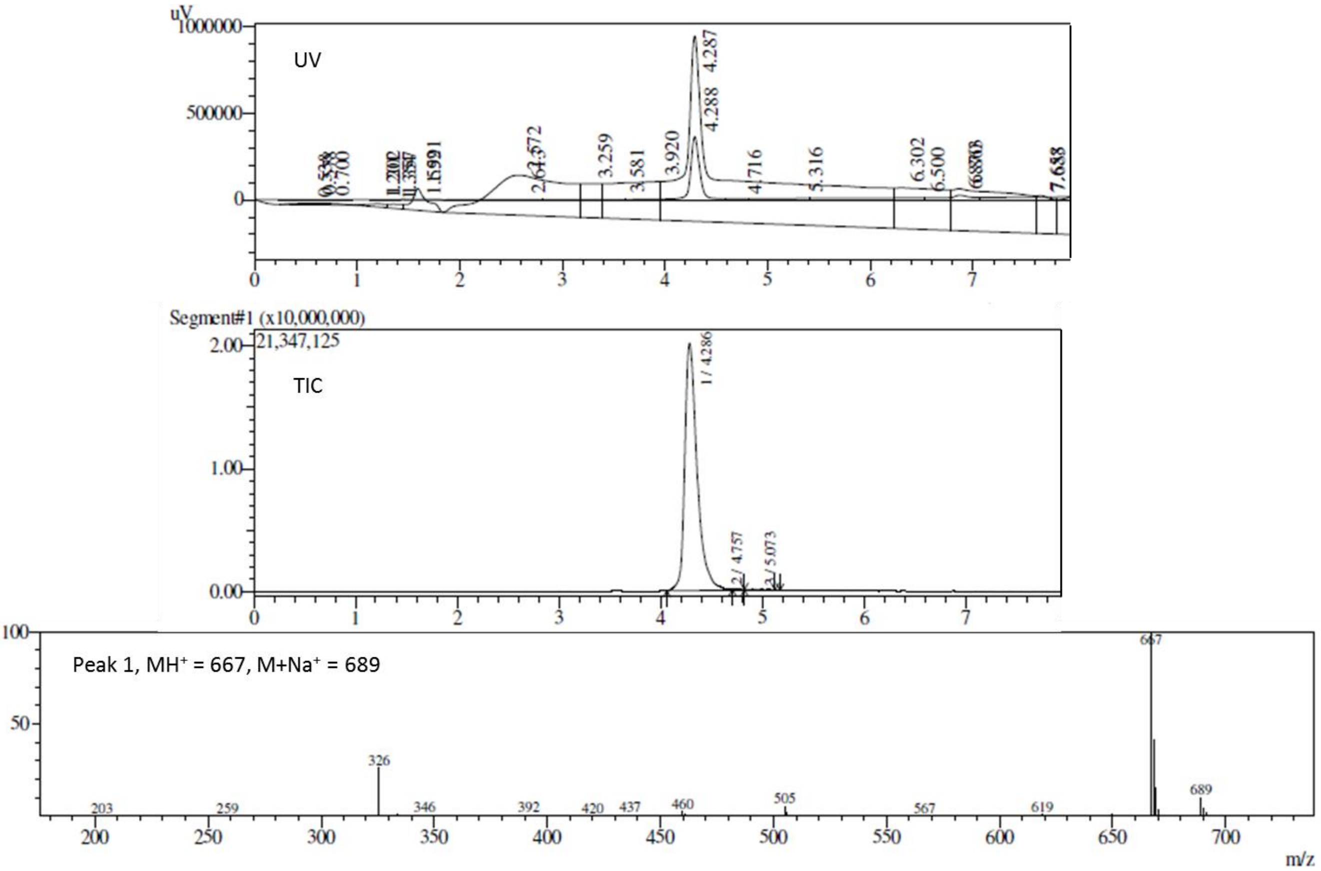
uV
UV
Segment#1 (x10,000,000)
21,347,125
TIC
1 / 4.286
2 / 4.757
3 / 5.073
Peak 1, MH+ = 667, M+Na+ = 689
326
505
667
689
m/z

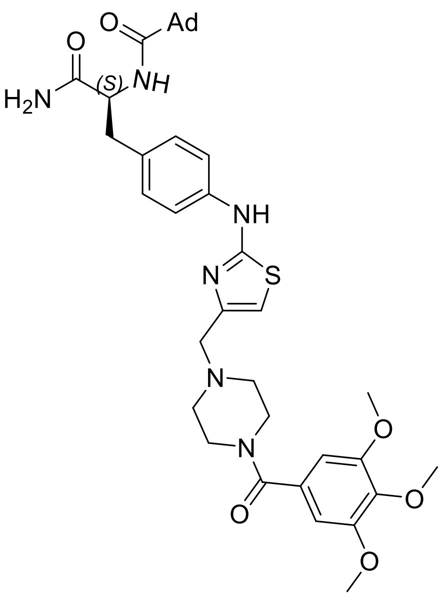

5n

$C_{38}H_{48}N_6O_6S$

Exact Mass: 716.34

Mol. Wt.: 716.89

5n (^{1}H NMR, 400 MHz, $CDCl_3$, 300 K) δ 7.14 (d, J = 5.5 Hz, 4H), 6.55 (s, 2H), 6.38 (s, 1H), 6.18 (d, J = 7.8 Hz, 1H), 5.87 (s_{broad}, 1H), 5.48 (s_{broad}, 1H), 5.23 (s, 2H), 4.56 (q, J = 7.7 Hz, 1H), 3.79 (s, 9H), 3.78 (s, 2H), 3.46 (d, J = 2.8 Hz, 2H), 2.98 (d, J = 6.7 Hz, 2H), 2.48 ($s_{v.broad}$, 2H), 1.95 (s_{broad}, 2H), 1.72 (s_{broad}, 7H), 1.63 (q, J = 15.8 Hz, 7H)

5n (^{13}C NMR, 100 MHz, $CDCl_3$, 300 K) δ 178.1, 173.1, 170.0, 165.0, 153.3, 139.4, 139.1, 131.4, 131.1, 130.4, 118.4, 105.4, 104.6, 60.9, 58.3, 56.3, 53.6, 40.7, 39.1, 37.3, 36.4, 28.0

LCMS: $C_{38}H_{48}N_6O_6S$ (M calculated) 716.89, $C_{38}H_{48}N_6O_6S$ (MH^+ found) 717

2317 - 18 PURE, 300K, CDCL3

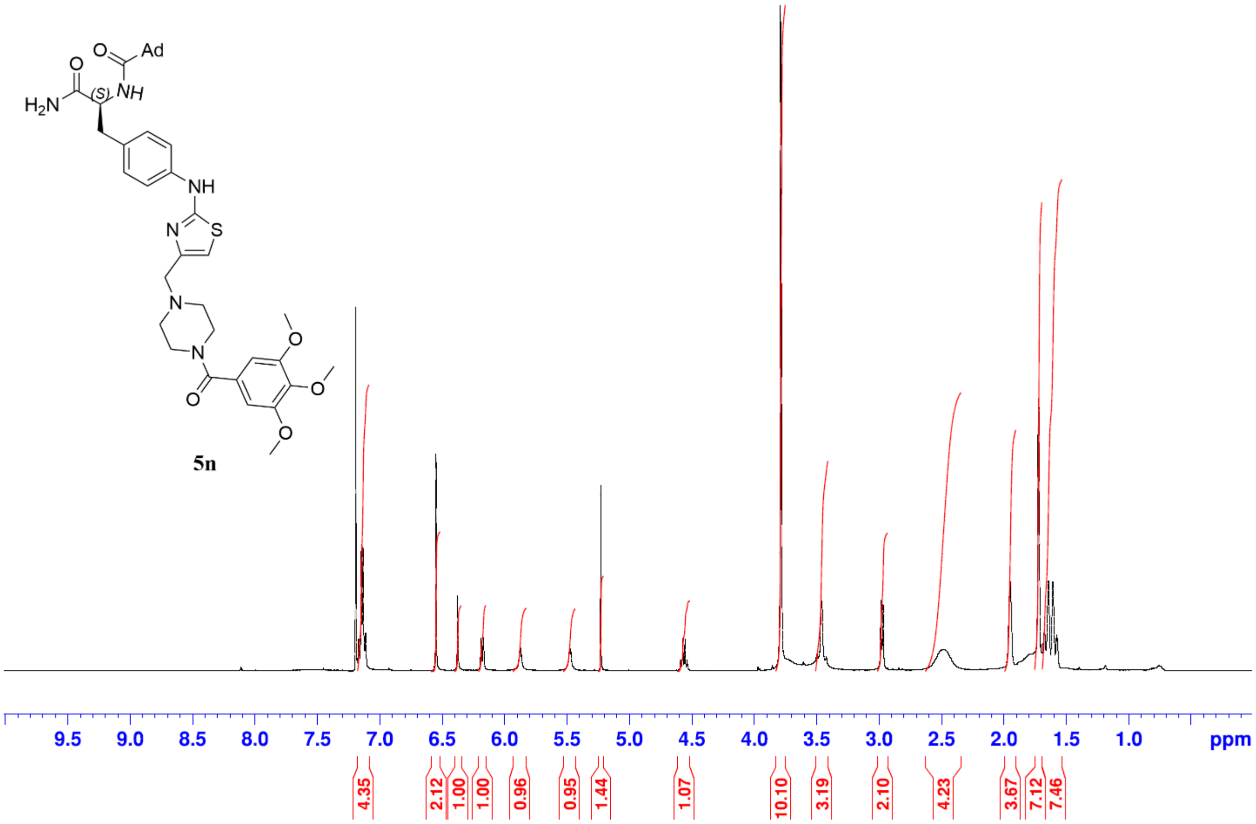

2317 - 18 PURE, 300K, CDCL3

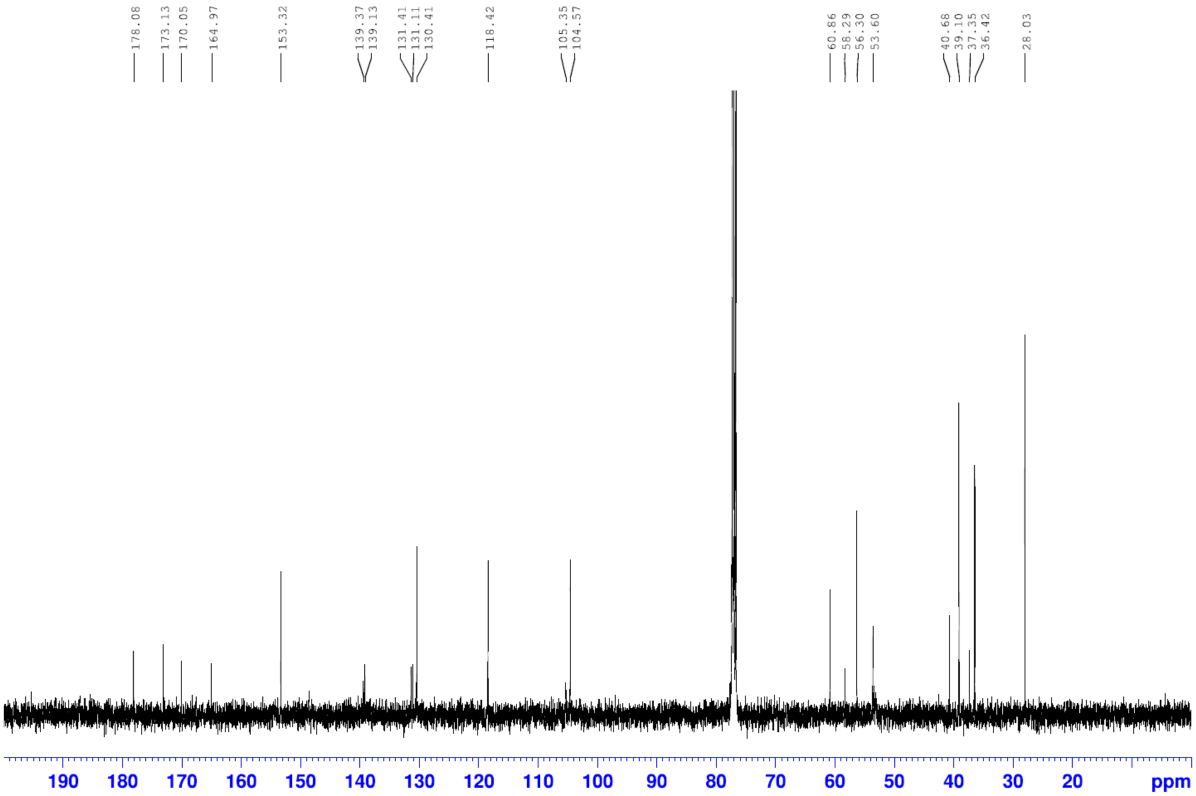

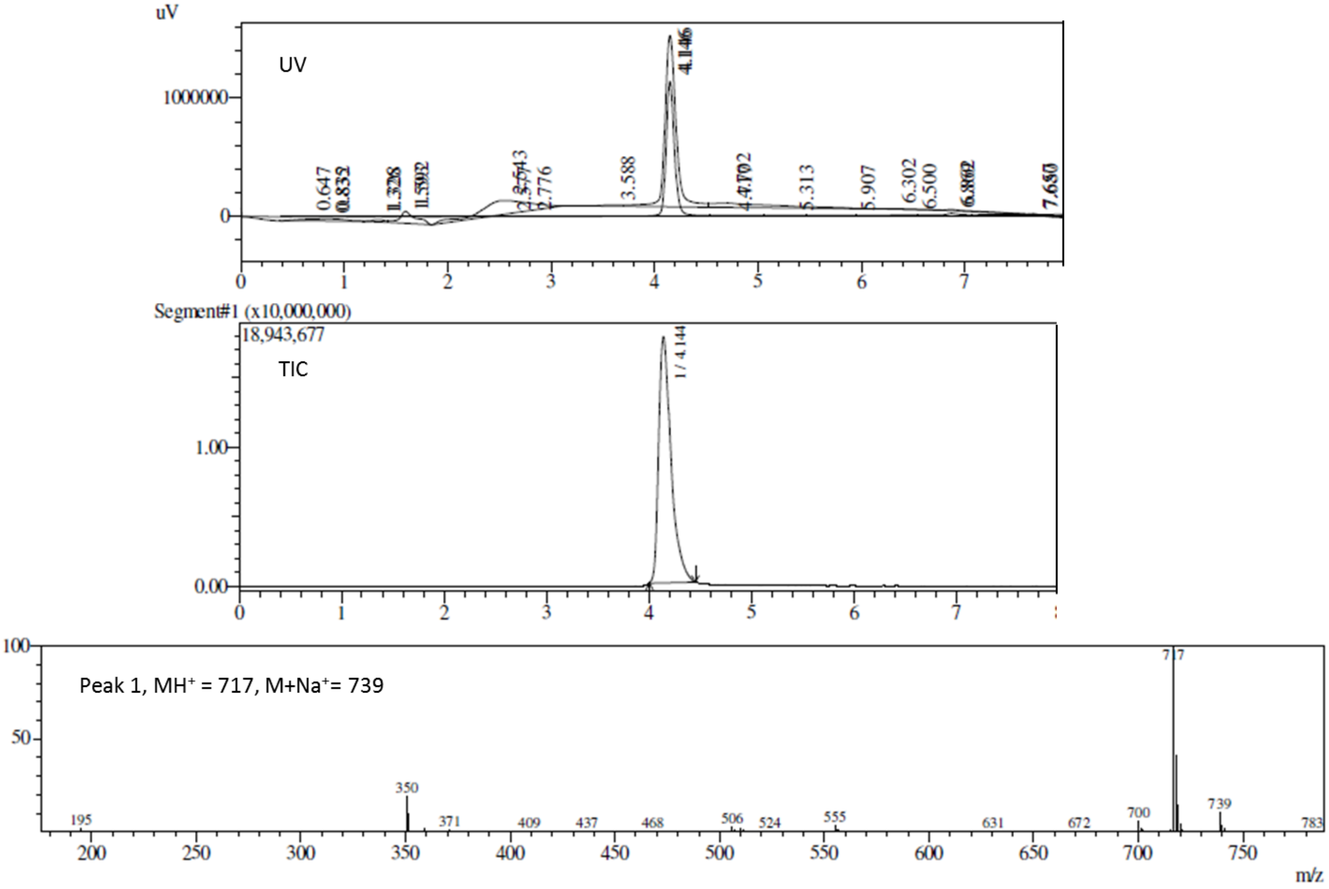

uV
UV
Segment#1 (x10,000,000)
18,943,677
TIC
1/ 4.144
Peak 1, MH+ = 717, M+Na+= 739
717
739
350
m/z

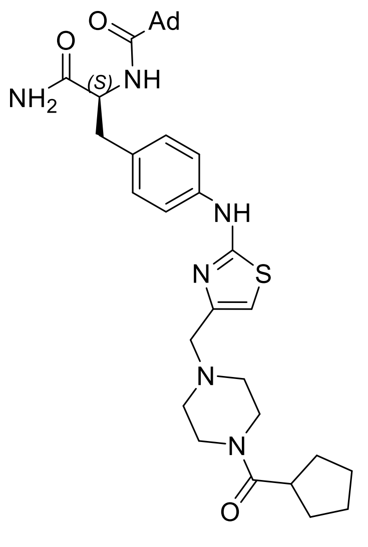

5o
$C_{34}H_{46}N_6O_3S$
Exact Mass: 618.34
Mol. Wt.: 618.83

5o (^{1}H NMR, 400 MHz, $CDCl_3$, 300 K) δ 7.23 (q, J = 10.6 Hz, 4H), 6.46 (s, 1H), 6.31 (d, J = 8.7 Hz, 1H), 6.05 (s_{broad}, 1H), 5.69 (s_{broad}, 1H), 5.32 (s, 1H), 4.67 (q, J = 6.7 Hz, 1H), 3.76 – 3.65 (m, 2H), 3.62-3.56 (m, 2H), 3.53 (d, J = 6.7 Hz, 2H), 3.01 (d, J = 6.3 Hz, 2H), 2.89 (pent. J = 7.9 Hz, 1H), 2.55 (s_{broad}, 2H), 2.51 (s_{broad}, 2H), 2.04 (s_{broad}, 4H), 1.82 (s, 9H), 1.72 (q, J = 14.1 Hz, 9H), 1.60 – 1.50 (m, 2H)

5o (^{13}C NMR, 100 MHz, $CDCl_3$, 300 K) δ 178.1, 174.5, 173.2, 165.0, 139.2, 131.3, 130.4, 118.4, 105.3, 58.3, 53.6, 53.5, 52.9, 45.3, 41.6, 41.0, 40.7, 39.1, 37.4, 36.4, 30.1, 28.0, 26.0

LCMS: $C_{34}H_{46}N_6O_3S$ (M calculated) 618.83, $C_{34}H_{47}N_6O_3S$ (MH^+ found) 619

2317 - 19 PURE, 300K, CDCL3

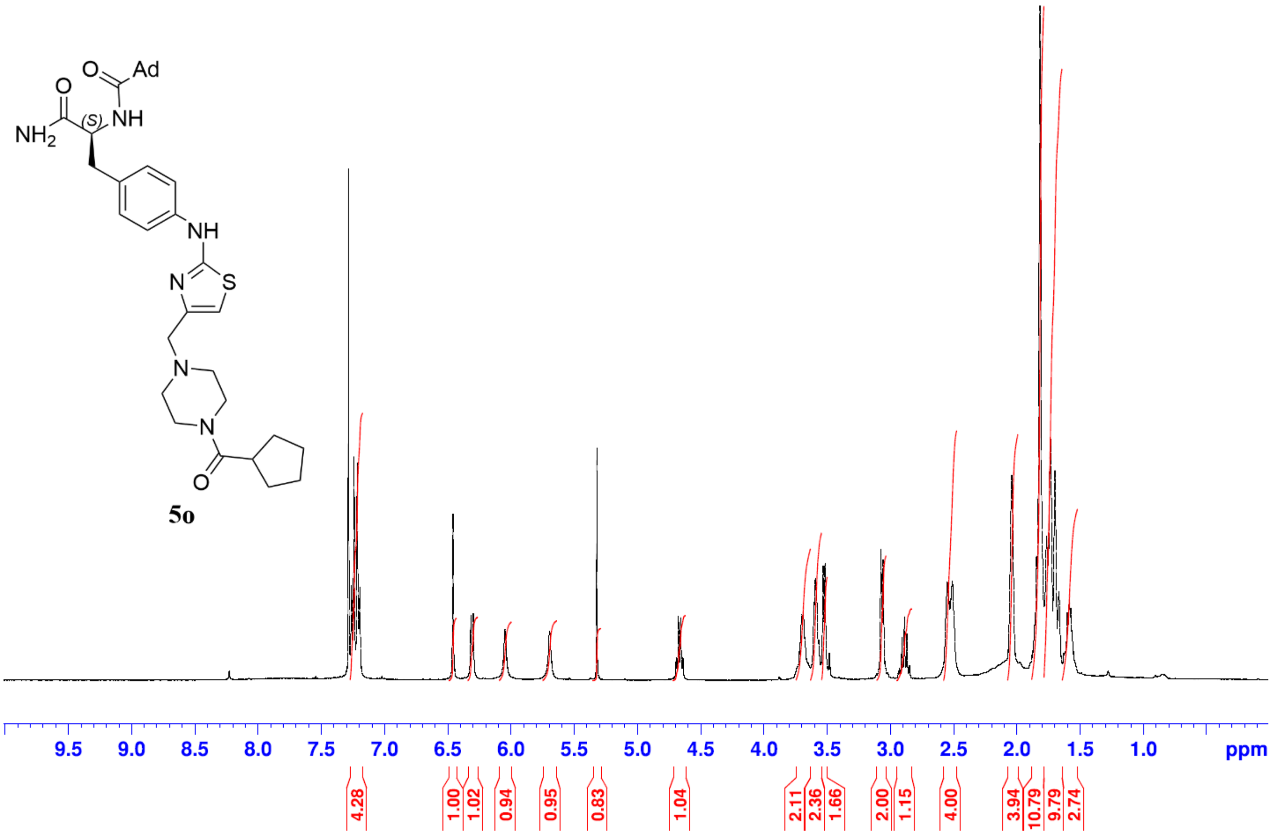

5o

2317 - 19 PURE, 300K, CDCL3

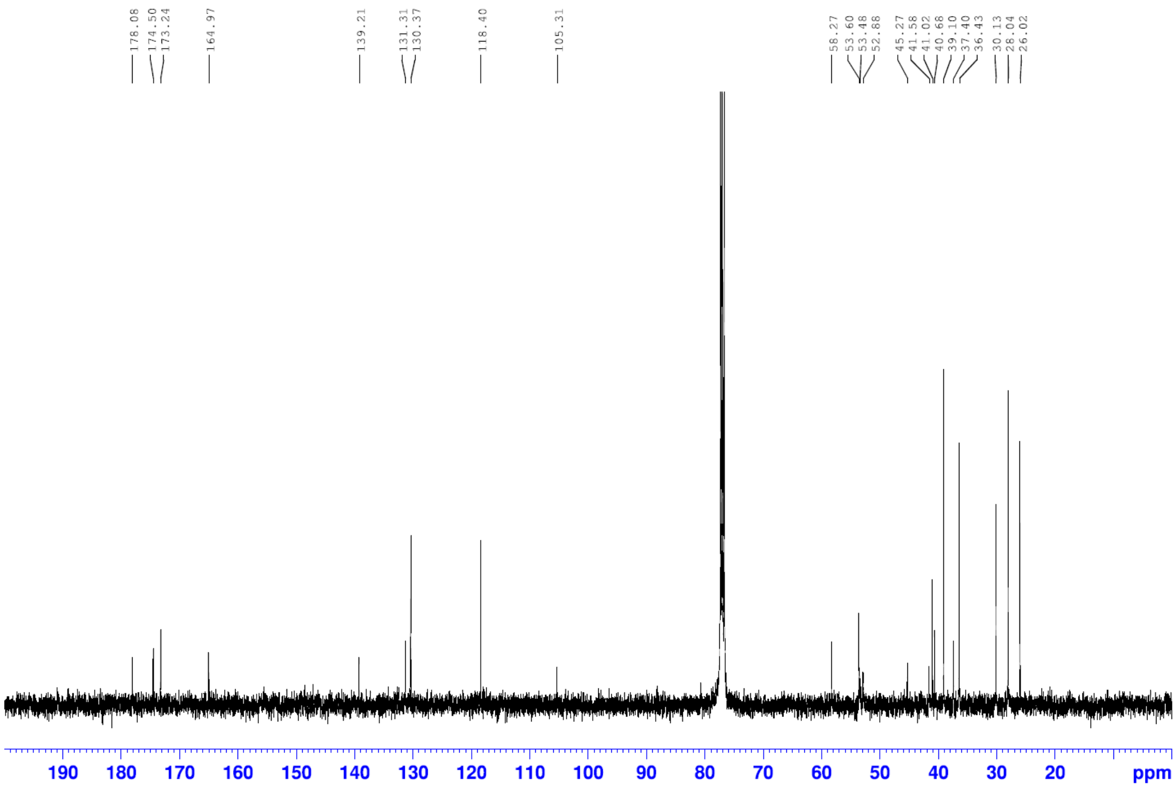

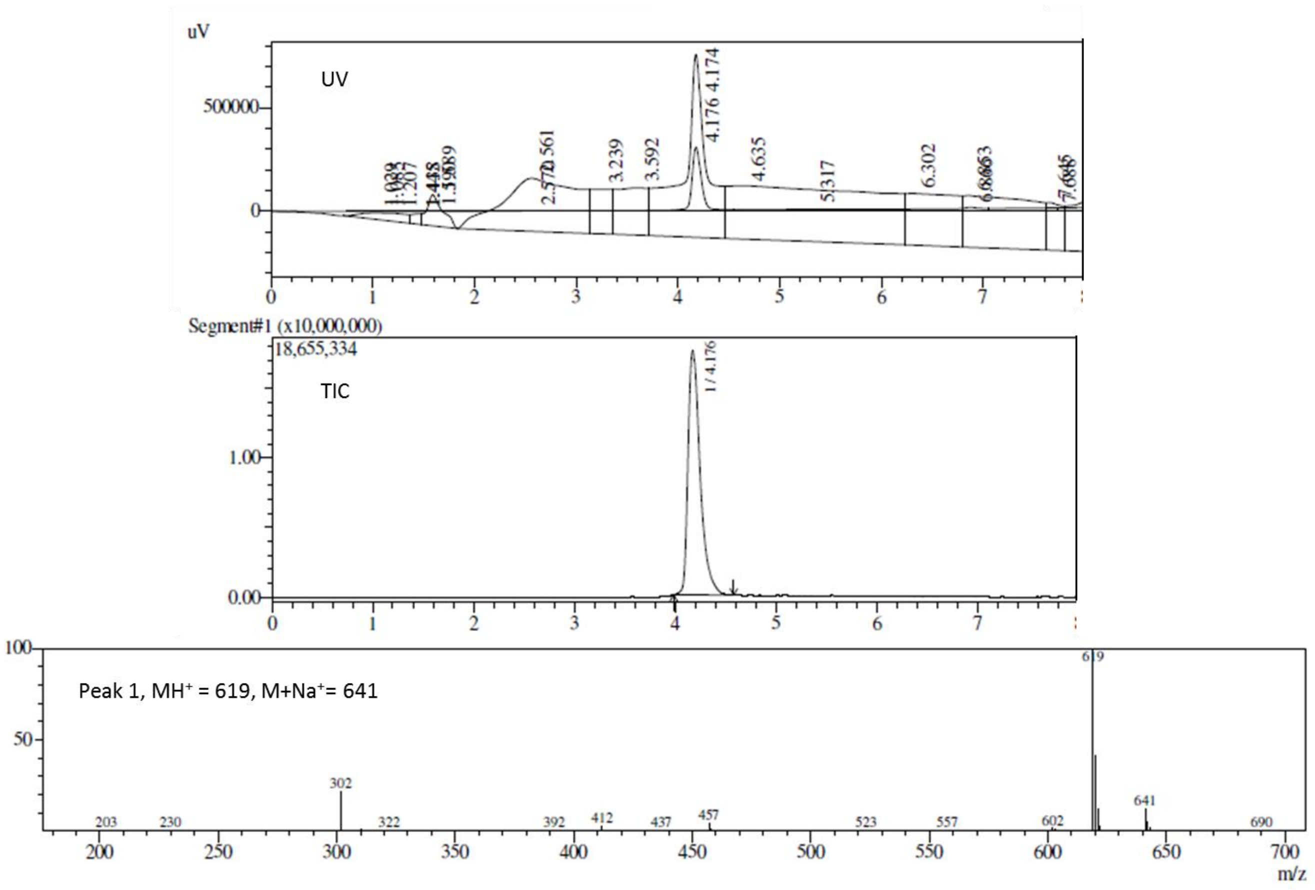
UV
TIC
Segment#1 (x10,000,000)
18,655,334
Peak 1, MH⁺ = 619, M+Na⁺= 641
m/z

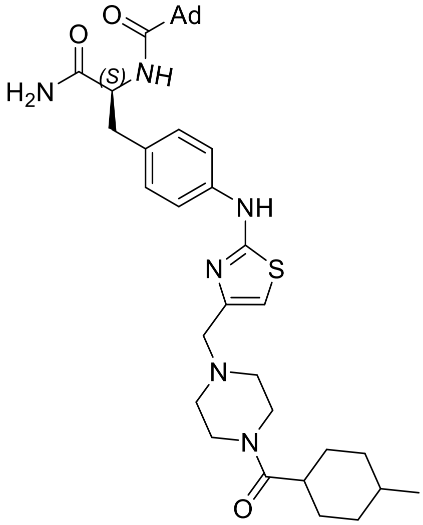

5p
$C_{36}H_{50}N_6O_3S$
Exact Mass: 646.37
Mol. Wt.: 646.89

5p (^{1}H NMR, 400 MHz, $CDCl_3$, 300 K) δ 7.27 (d, J = 15.5 Hz, 4H), 6.46 (s, 1H), 6.23 (d, J = 8.4 Hz, 1H), 5.87 (s_{broad}, 1H), 5.42 (s_{broad}, 1H), 4.65 (q, J = 8.4 Hz, 1H), 3.68 (s_{broad}, 2H), 3.56 (s_{broad}, 2H), 3.52 (pent., J = 6.9 Hz, 1H), 3.13-3.1 (m, 2H), 2.54 (s_{broad}, 4H), 2.05 (s, 4H), 1.82 (s, 9H), 1.73 (q, J = 14.7 Hz, 6H), 1.58 – 1.47 (m, 6H), 1.30 (s, 2H), 1.23 (t, J = 7.3 Hz, 2H), 1.0 (d, J = 5.5 Hz, 3H), 0.94-0.86 (m, 3H)

5p (^{13}C NMR, 100 MHz, $CDCl_3$, 300 K) δ 178.1, 173.1, 139.1, 131.4, 130.4, 118.5, 105.3, 65.8, 58.3, 53.6, 40.7, 39.1, 38.8, 37.3, 36.4, 31.6, 31.1, 28.4, 28.0, 24.9, 22.6, 18.9, 15.2, 14.1

LCMS: $C_{36}H_{50}N_6O_3S$ (M calculated) 646.89, $C_{36}H_{51}N_6O_3S$ (MH^+ found) 647

2317 - 20 PURE, 300K, CDCL3

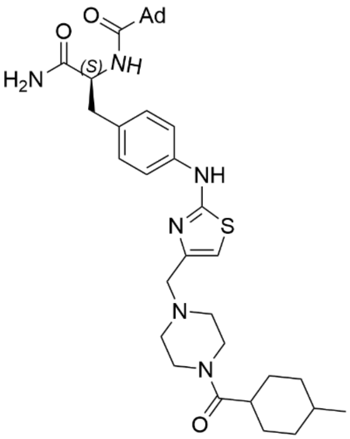

5p

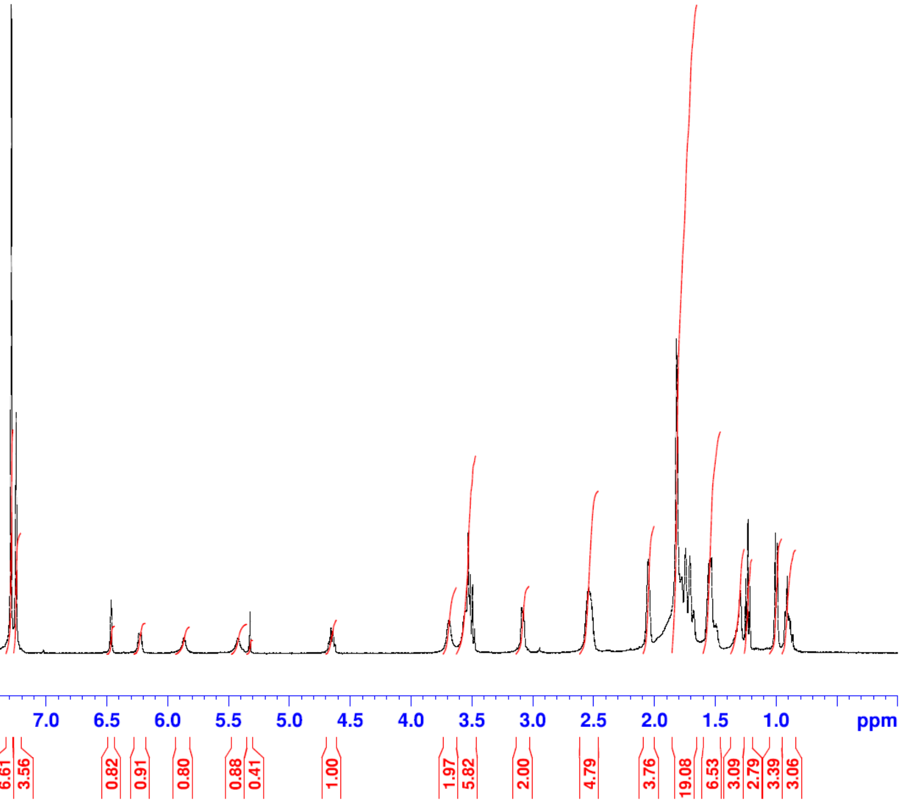

2317 - 20 PURE, 300K, CDCL3

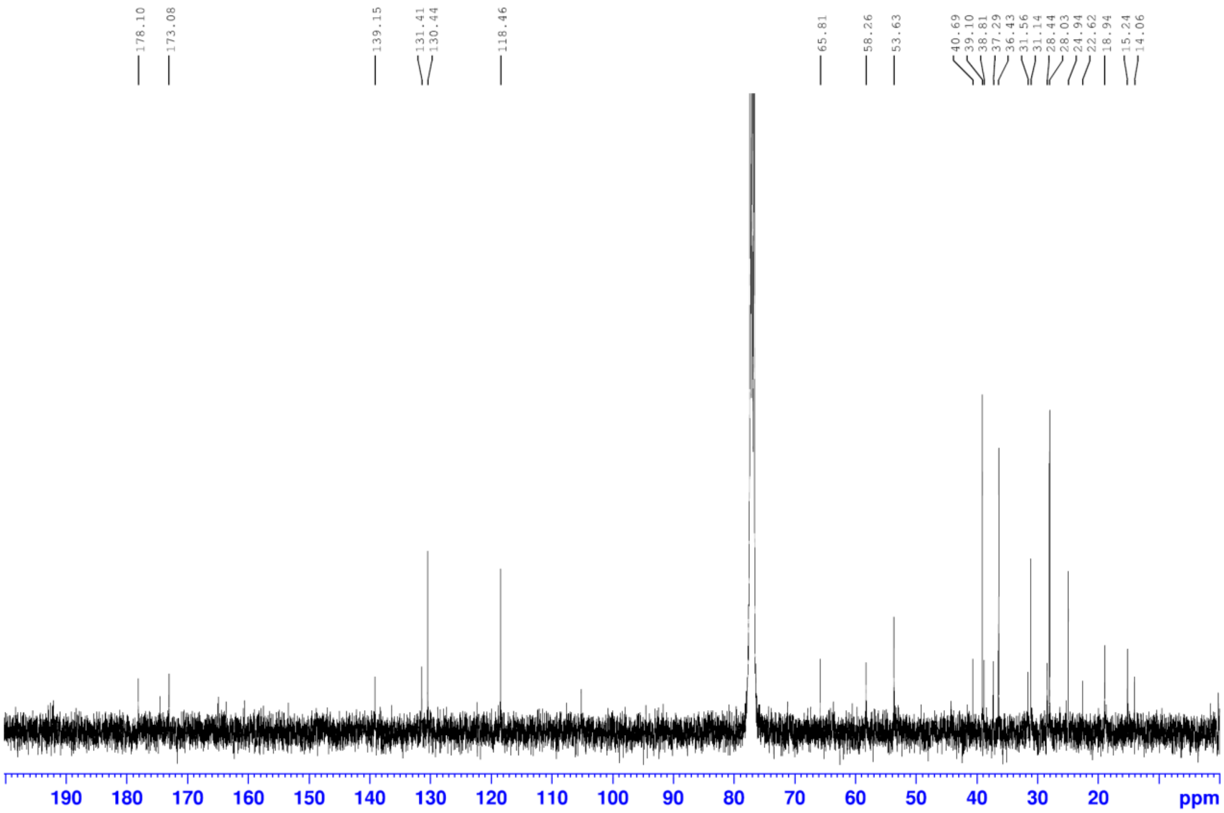

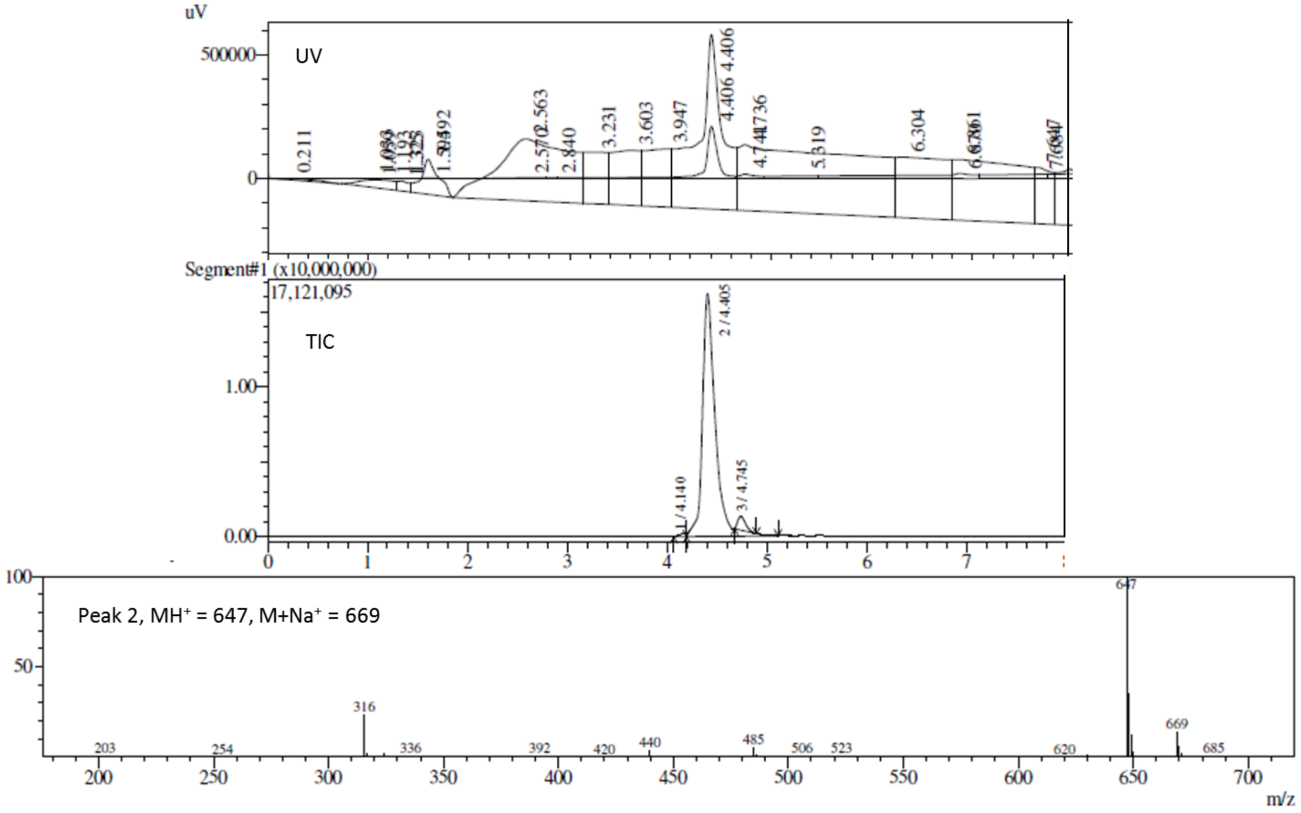

UV
TIC
Peak 2, MH⁺ = 647, M+Na⁺ = 669

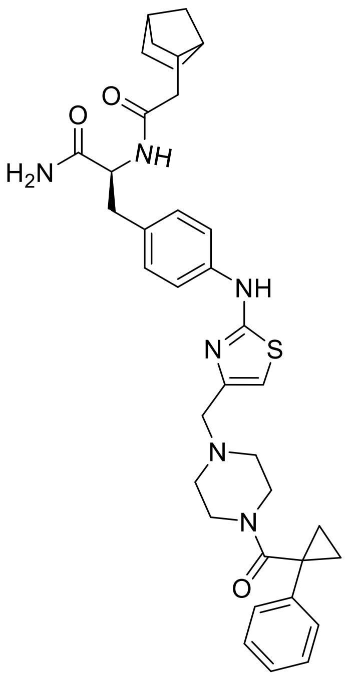

5q

$C_{36}H_{44}N_6O_3S$

Exact Mass: 640.32

Mol. Wt.: 640.84

5q (^{1}H NMR, 400 MHz, $CDCl_3$, 300 K) δ 7.31 (d, J = 7.5 Hz, 2H), 7.24 – 7.20 (m, 5H), 7.16 (d, J = 7.3 Hz, 2H), 6.41 (s, 1H), 6.17 (d, J = 7.2 Hz, 1H), 5.96 (s_{broad}, 1H), 5.60 (s_{broad}, 1H), 4.69 (q, J = 7.2 Hz, 1H), 3.72 (s_{broad}, 2H), 3.50 (q, J = 7.7 Hz, 4H), 3.44 (s, 2H), 3.07 (d, J = 8.3 Hz, 2H), 2.50 (s_{broad}, 2H), 2.51 (s_{broad}, 2H), 2.16 (dd, J_{AB} = 7.0 Hz, J_{BC} = 7.6 Hz, 1H), 2.03 (dd, J_{AB} = 8.7 Hz, J_{BC} = 6.5 Hz, 1H), 1.89 – 1.80 (m, 2H), 1.49-1.40 (m, 5H), 1.33 – 1.26 (m, 3H), 1.23 (t, J = 7.2 Hz, 4H), 1.21 – 1.16 (m, 3H), 1.15 – 1.06 (m, 3H), 1.06 – 0.98 (m, 2H), 0.93 – 0.86 (m 3H)

5q (^{13}C NMR, 100 MHz, $CDCl_3$, 300 K) δ 173.0, 172.6, 171.0, 164.8, 140.7, 139.1, 131.2, 130.3, 128.7, 126.3, 125.3, 118.3, 65.8, 58.1, 53.8, 52.6, 43.5, 41.0, 38.9, 37.7, 37.4, 36.7, 35.2, 31.6, 29.8, 29.4, 28.5, 22.6, 15.2, 15.2, 14.1

LCMS: $C_{36}H_{44}N_6O_3S$ (M calculated) 640.84, $C_{36}H_{45}N_6O_3S$ (MH^+ found) 641

2317 - 21 PURE, 300K, CDCL3

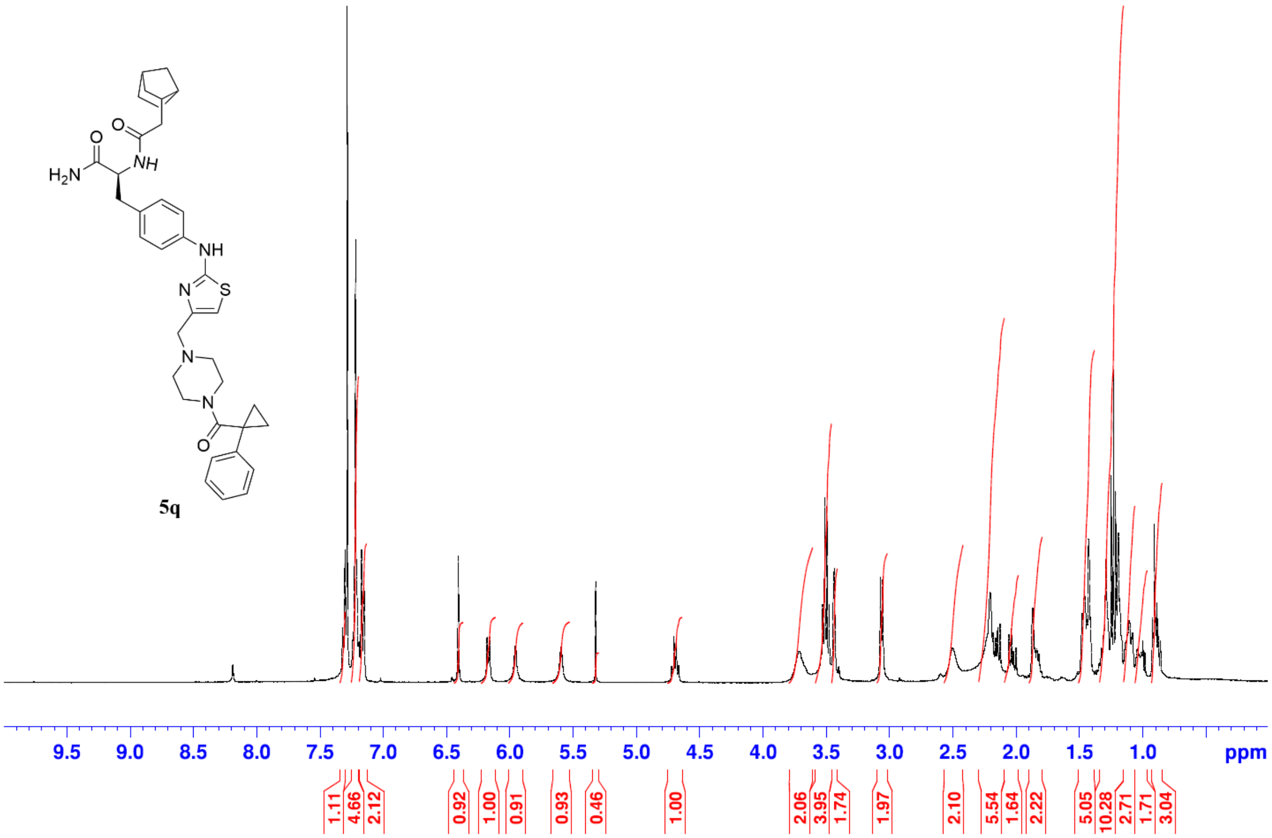

2317 - 21 PURE, 300K, CDCL3

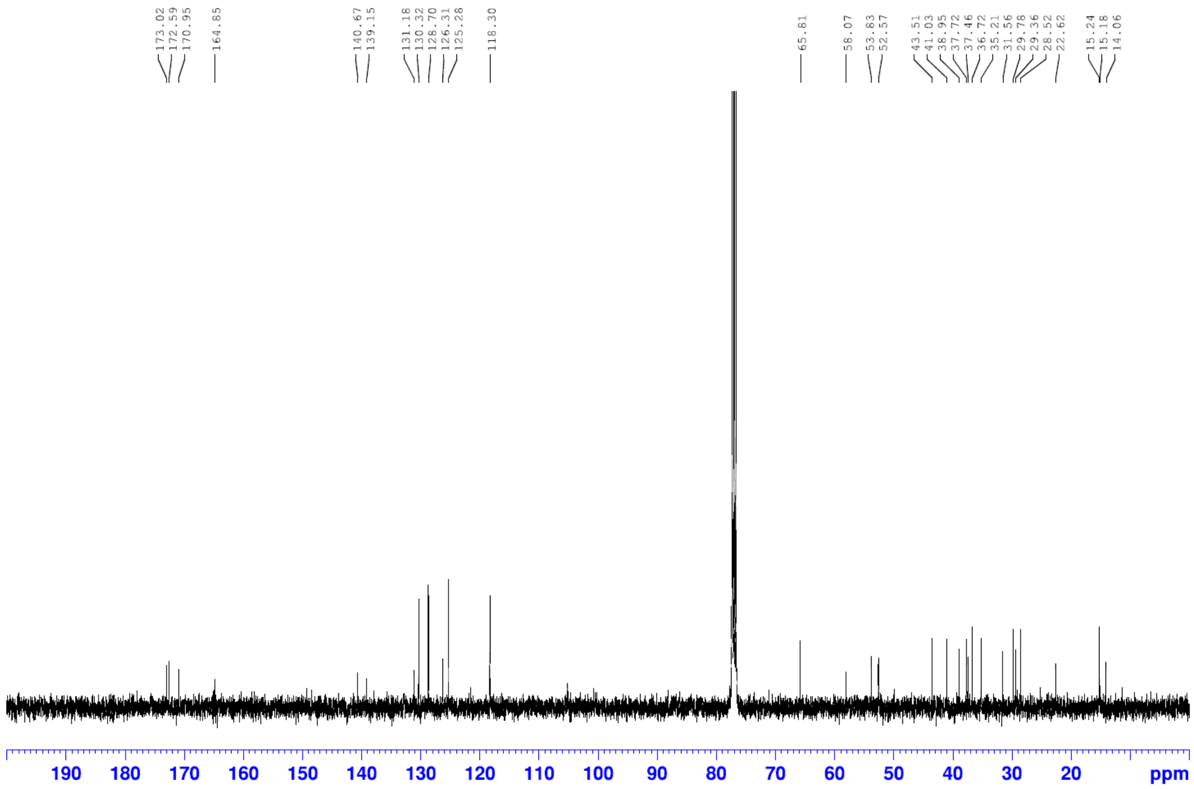

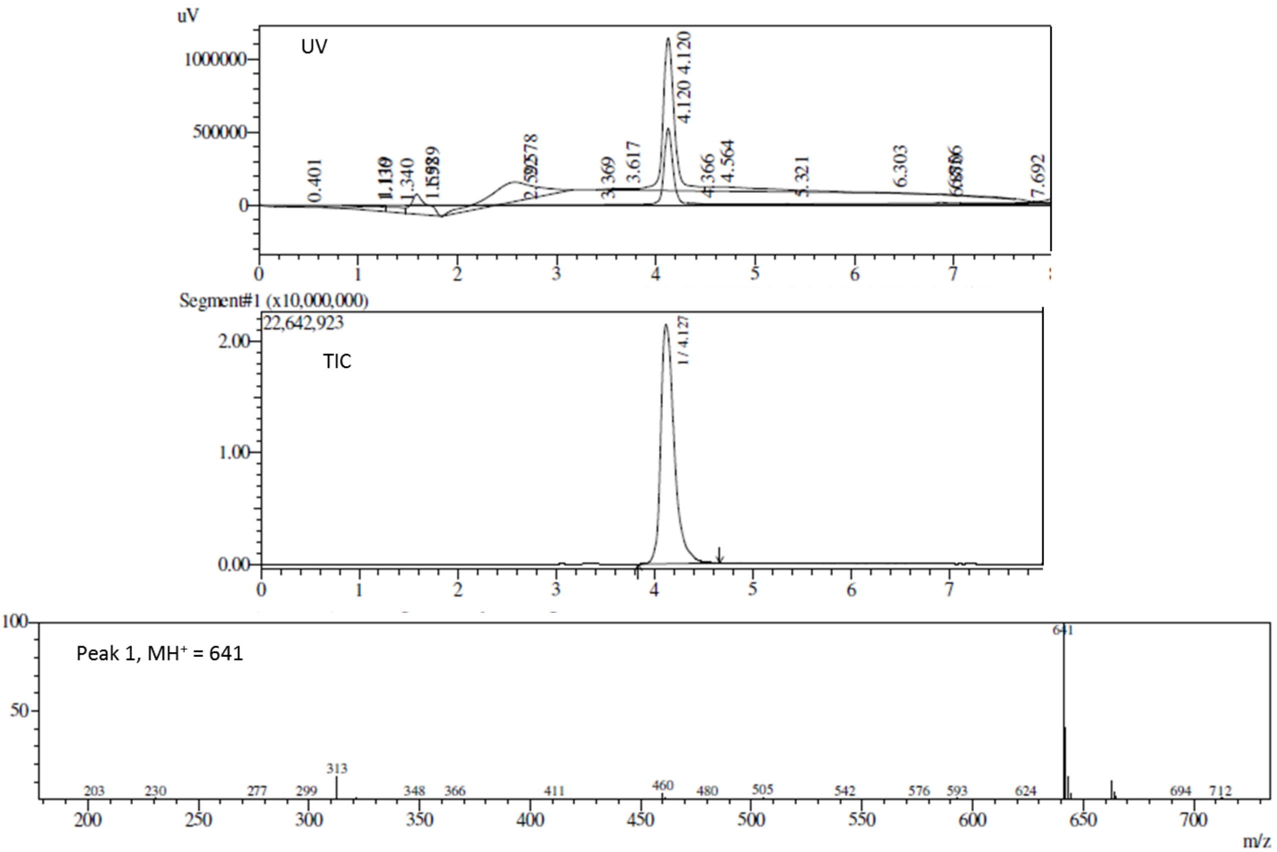
uV
UV
TIC
Segment#1 (x10,000,000)
22,642,923
Peak 1, MH+ = 641
m/z

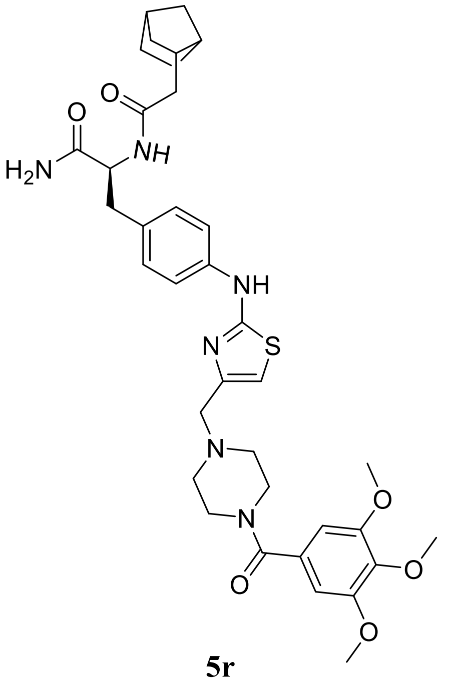

5r

$C_{36}H_{46}N_6O_6S$
Exact Mass: 690.32
Mol. Wt.: 690.85

5r (^{1}H NMR, 400 MHz, $CDCl_3$, 300 K) δ 7.23 (d, J = 9.2 Hz, 4H), 6.64 (s, 2H), 6.47 (s, 1H), 6.20 (t, J = 7.4 Hz, 1H), 5.97 (s_{broad}, 1H), 5.63 (s_{broad}, 1H), 4.69 (q, J = 8.0 Hz, 1H), 3.88 (s, 9H), 3.87 (s, 2H), 3.55 (q, J = 7.5 Hz, 3H), 3.50 (Q, J = 7.0 Hz, 1H), 3.06 (d, J = 8.0 Hz, 2H), 2.57 ($s_{v.broad}$, 4H), 2.24 – 2.18 (m, 1H), 2.16 (dd, J_{AB} = 4.2 Hz, J_{BC} = 3.8 Hz, 1H), 2.13 (s, 1H), 2.04 (dd, J_{AB} = 3.4 Hz, J_{BC} = 4.2 Hz, 1H), 1.95 (s,1H), 1.90 – 1.80 (m, 4H), 1.51 – 1.42 (m, 3H), 1.33 – 1.26 (m, 2H), 1.23 (t, J = 7.2 Hz, 3H), 1.20 – 0.97 (m, 6H), 0.93 – 0.86 (m, 1H)

5r (^{13}C NMR, 100 MHz, $CDCl_3$, 300 K) δ 173.0, 172.6, 170.1, 153.3, 139.4, 139.2, 131.2, 131.1, 130.3, 118.3, 118.2, 105.4, 104.6, 60.9, 58.3, 56.3, 53.8, 43.5, 41.1, 41.0, 38.9, 37.7, 37.5, 36.7, 35.2, 35.2, 29.8, 28.5

LCMS: $C_{36}H_{46}N_6O_6S$ (M calculated) 690.85, $C_{36}H_{47}N_6O_6S$ (MH^+ found) 691

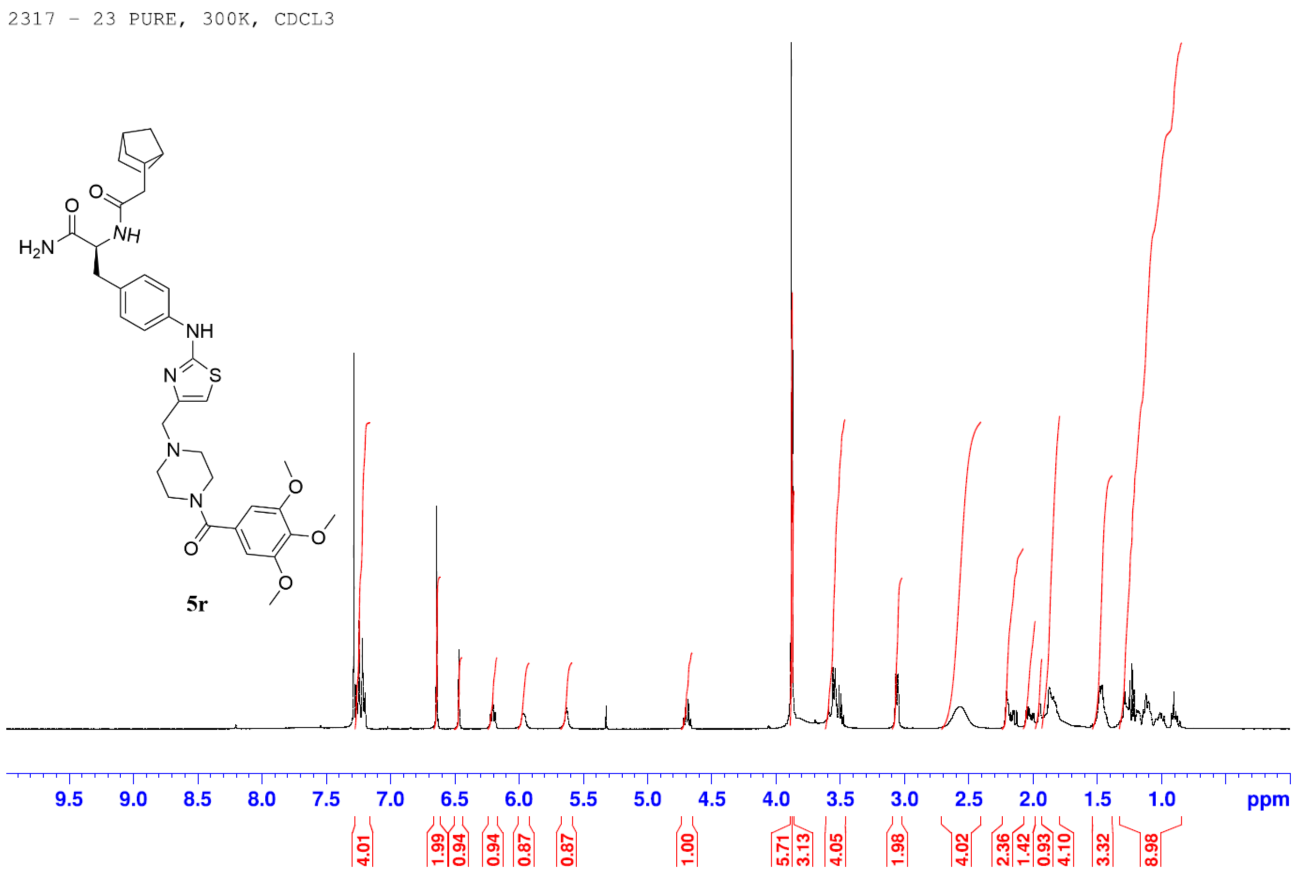

2317 - 23 PURE, 300K, CDCL3
5r
H2N
O
NH
NH
N
S
N
N
O
O
O
O
9.5 9.0 8.5 8.0 7.5 7.0 6.5 6.0 5.5 5.0 4.5 4.0 3.5 3.0 2.5 2.0 1.5 1.0 ppm
4.01 1.99 0.94 0.94 0.87 0.87 1.00 5.71 3.13 4.05 1.98 4.02 2.36 1.42 0.93 4.10 3.32 8.98

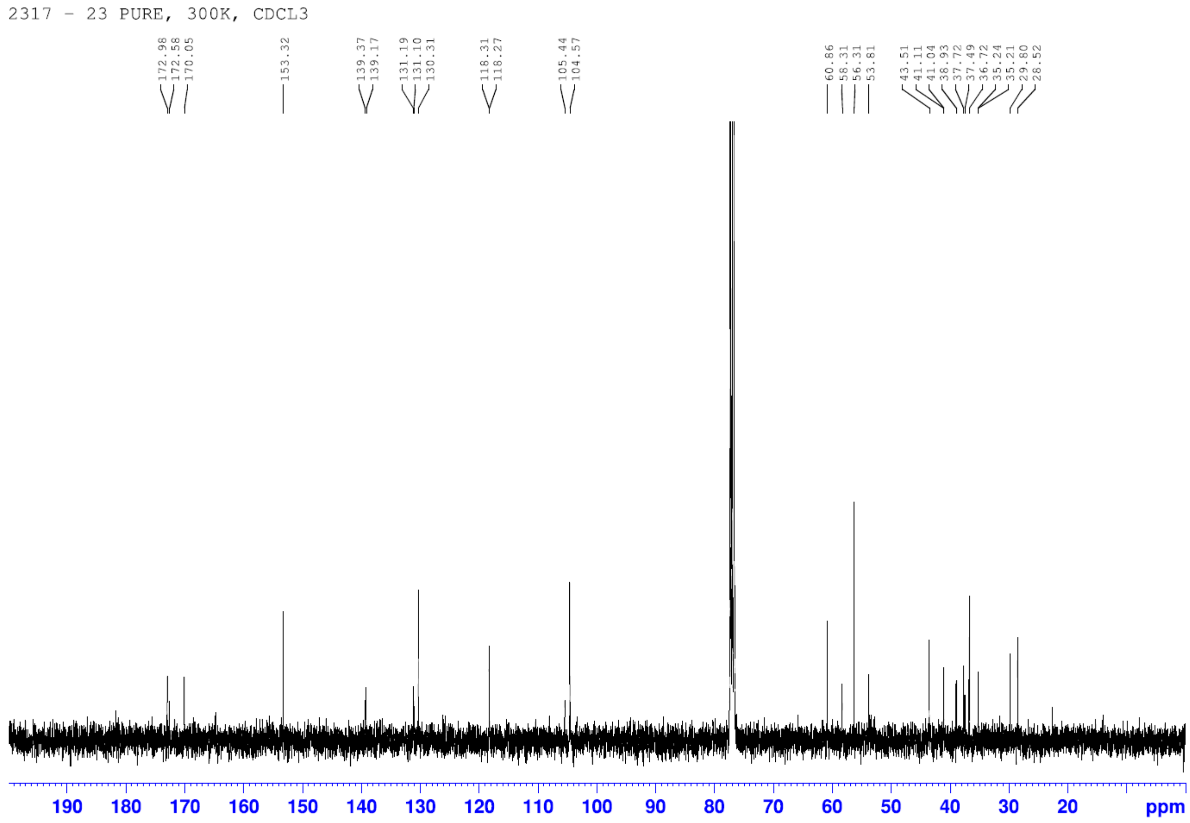

2317 - 23 PURE, 300K, CDCL3
172.98
172.58
170.05
153.32
139.37
139.17
131.19
131.10
130.31
118.31
118.27
105.44
104.57
60.86
58.31
56.31
53.81
43.51
41.11
41.04
38.93
37.72
37.49
36.72
35.24
35.21
29.80
28.52
190 180 170 160 150 140 130 120 110 100 90 80 70 60 50 40 30 20 ppm

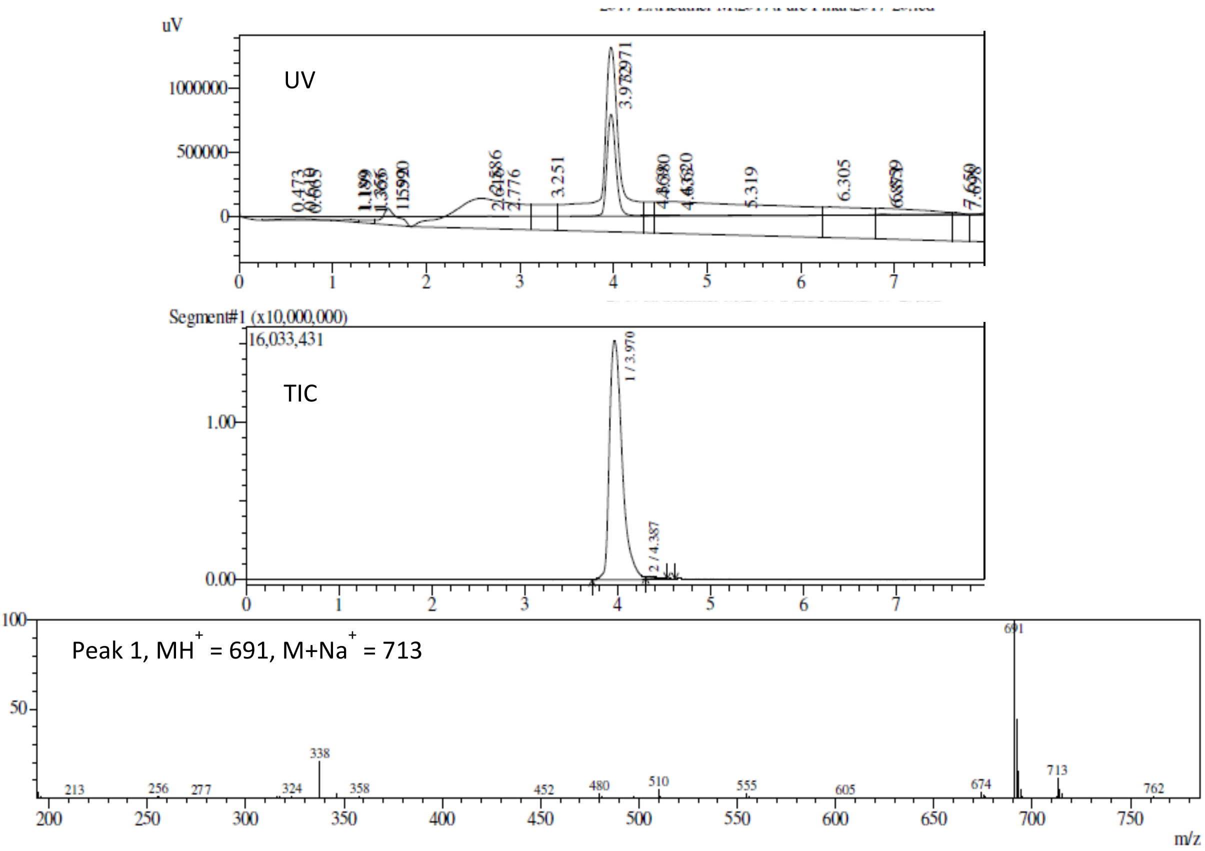

uV
UV
1000000
500000
0
Segment#1 (x10,000,000)
16,033,431
TIC
1.00
0.00
1 / 3.970
2 / 4.387
100
50
Peak 1, MH+ = 691, M+Na+ = 713
691
338
713
m/z

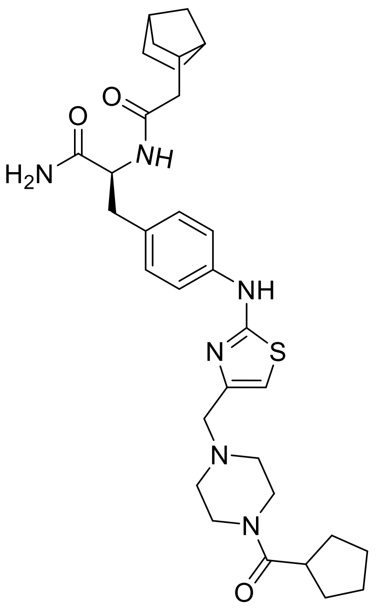

5s
$C_{32}H_{44}N_6O_3S$
Exact Mass: 592.32
Mol. Wt.: 592.80

5s (^{1}H NMR, 400 MHz, $CDCl_3$, 300 K) δ 7.24 (q, J = 7.2 Hz, 4H), 6.46 (s, 1H), 6.17 (t, J = 7.7 Hz, 1H), 5.91 (s_{broad}, 1H), 5.56 (s_{broad}, 1H), 4.69 (q, J = 7.3 Hz, 1H), 3.73 – 3.66 (m, 2H), 3.62 – 3.55 (m, 2H), 3.54 – 3.78 (m, 3H), 3.10 – 3.05 (m, 2H), 2.90 (pent J = 7.9 Hz, 1H), 2.58 – 2.47 (m, 4H), 2.24 – 2.13 (m, 2H), 2.07 – 1.99 (m, 1H), 1.95 (s_{broad}, 1H), 1.90 – 1.70 (m, 13H), 1.65 – 1.54 (m, 2H), 1.52 – 1.42 (m, 4H), 1.34 – 1.07 (m, 8H), 0.94 – 0.86 (m, 1H)

5s (^{13}C NMR, 100 MHz, $CDCl_3$, 300 K) δ 174.5, 173.0, 139.2, 131.1, 130.3, 118.3, 105.2, 75.0, 65.8, 58.4, 53.8, 53.5, 52.9, 45.4, 43.5, 41.7, 41.1, 41.0, 38.9, 37.7, 37.5, 36.7, 35.2, 31.5, 30.8, 30.1, 29.8, 28.5, 26.0, 15.2, 14.0

LCMS: $C_{32}H_{44}N_6O_3S$ (M calculated) 592.80, $C_{32}H_{45}N_6O_3S$ (MH^+ found) 593

2317 - 24 PK 1 PURE, 300K, CDCL3

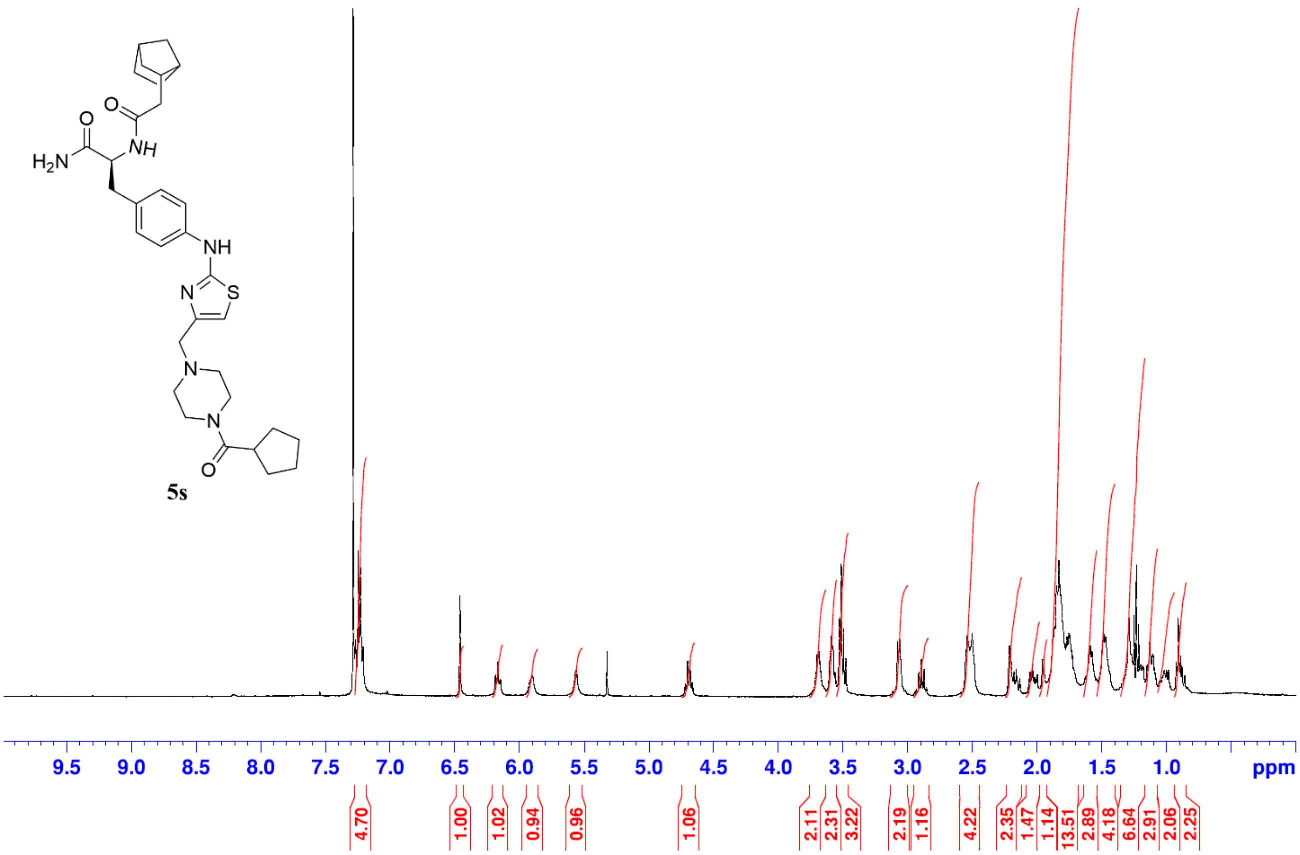

2317 - 24 PK 1 PURE, 300K, CDCL3

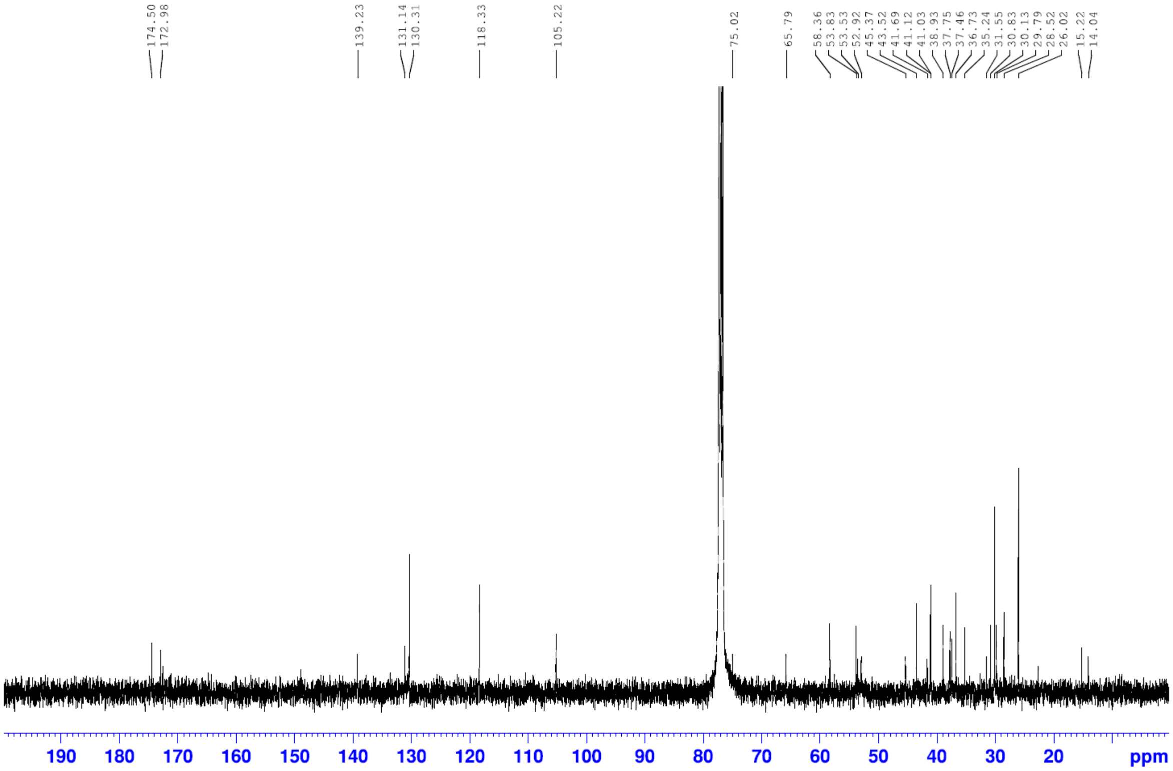

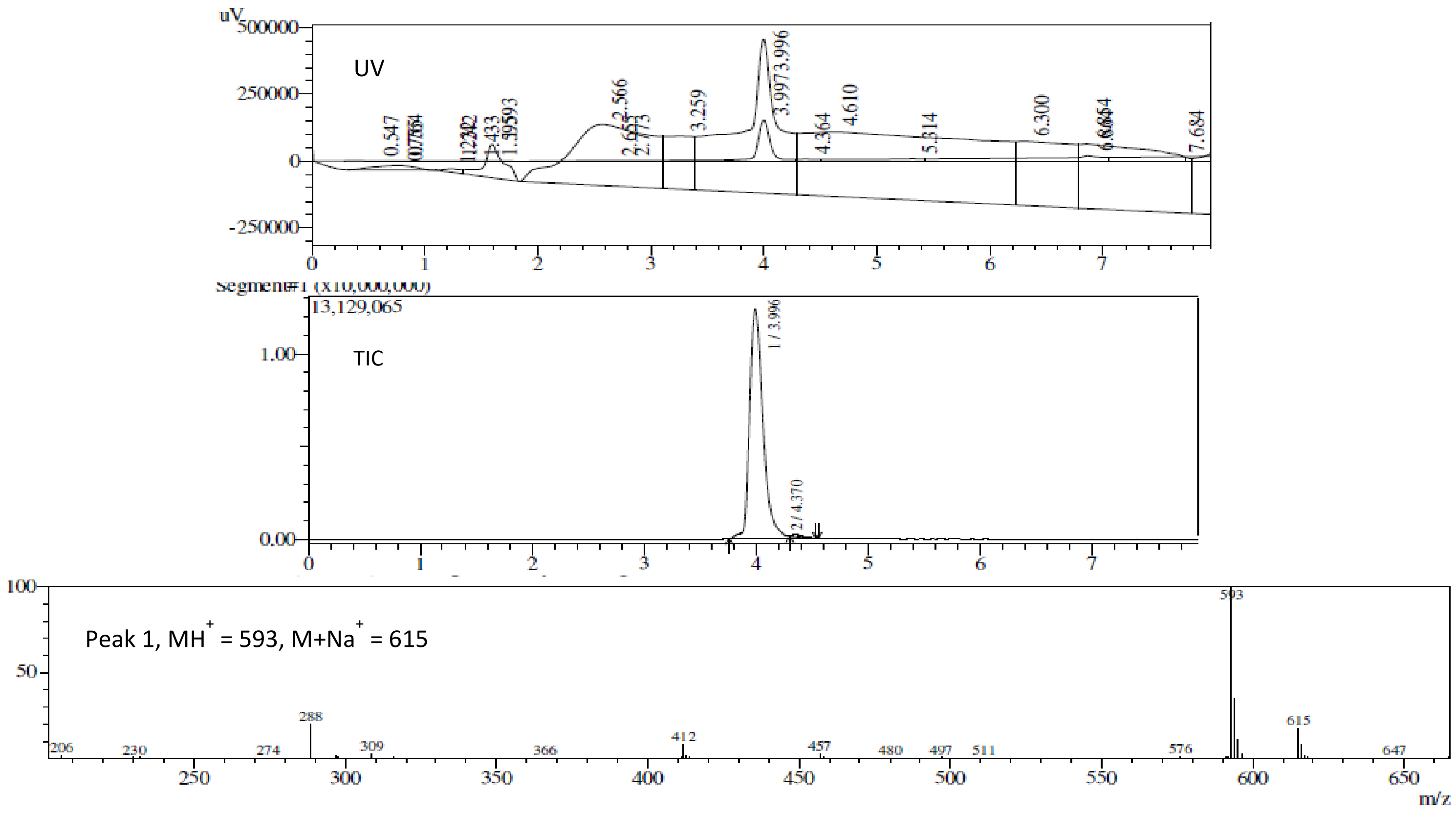
UV
TIC
Peak 1, MH+ = 593, M+Na+ = 615
m/z

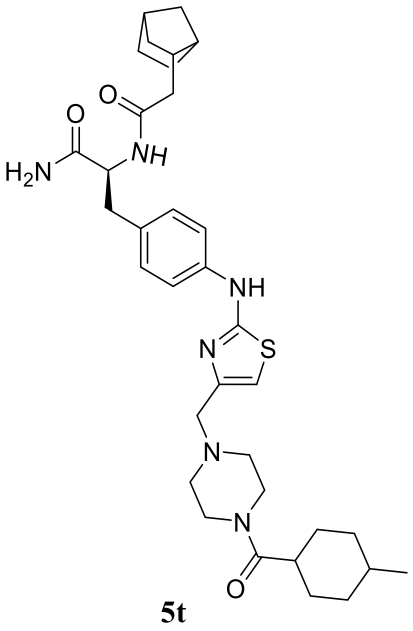

5t
$C_{34}H_{48}N_6O_3S$
Exact Mass: 620.35
Mol. Wt.: 620.85

5t (¹H NMR, 400 MHz, CDCl$_3$, 300 K) δ 7.23 (q, J = 12.7 Hz, 4H), 6.46 (s, 1H), 6.24 (t, J = 9.5 Hz, 1H), 6.0 (d, J = 7.4 Hz, 1H), 6.68 (s_{broad}, 1H), 4.70 (q, J = 7.4 Hz, 1H), 3.67 ($s_{v.\ broad}$, 2H), 3.59-3.48 (m, 4H), 3.06 (d, J = 7.4 Hz, 2H), 2.54 (s_{broad}, 2H), 2.50 (s_{broad}, 2H), 2.23 (s_{broad}, 2H), 2.19 (s, 2H), 2.17-2.13 (m, 1H), 2.07-1.99 (m, 1H), 1.95 (s_{borad}, 2H), 1.87 (s_{broad}, 2H), 1.85 – 1.70 (m, 6H), 1.57-1.42 (m, 8H), 1.32 – 1.06 (m, 7H), 0.99 (d, J = 7.5 Hz, 3H), 094-0.88 (m, 3H)

5t (¹³C NMR, 100 MHz, CDCl$_3$, 300 K) δ 174.5, 173.0, 172.6, 139.2, 131.1, 130.3, 118.3, 118.2, 105.3, 58.3, 53.8, 45.2, 43.5, 41.1, 41.0, 40.2, 39.0, 38.8, 37.7, 37.5, 36.7, 35.2, 31.1, 29.8, 29.3, 28.5, 28.4, 24.9, 22.6, 18.9

LCMS: $C_{34}H_{48}N_6O_3S$ (M calculated) 620.85, $C_{34}H_{49}N_6O_3S$ (MH^+ found) 621

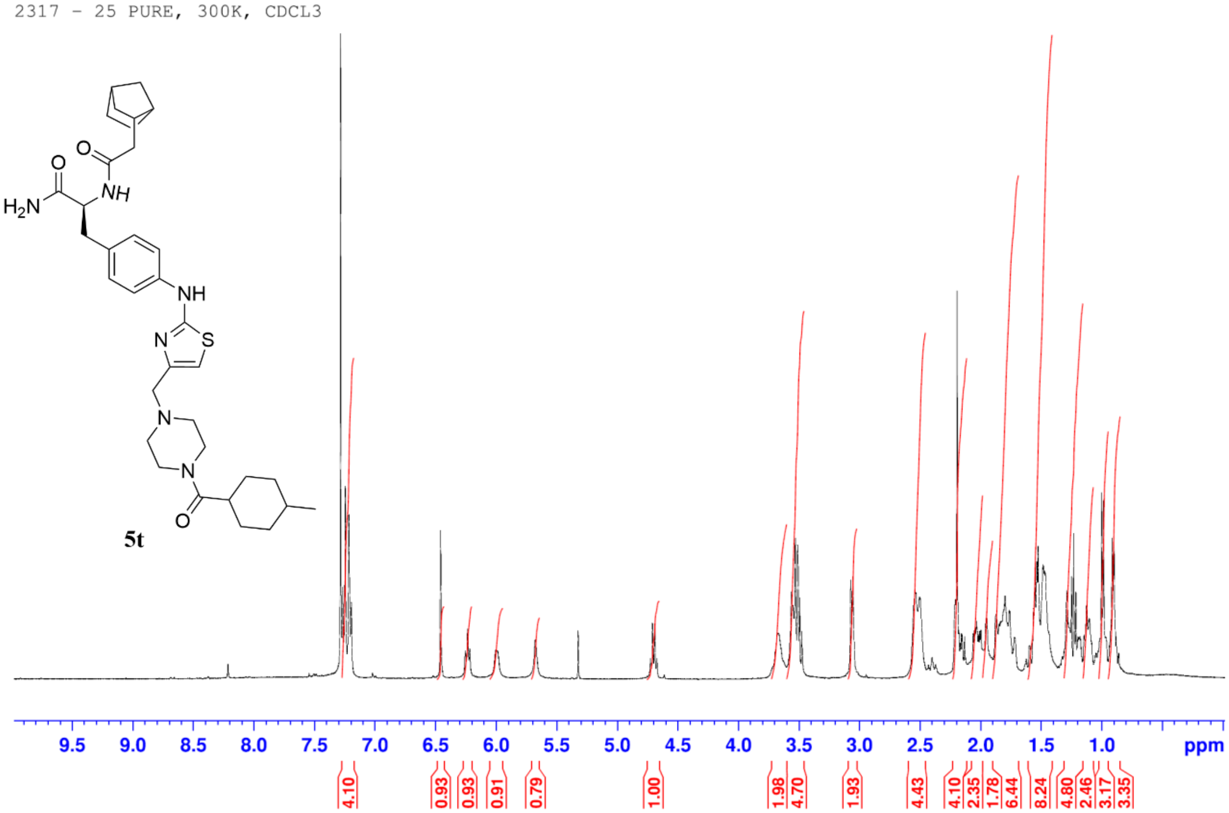
2317 - 25 PURE, 300K, CDCL3
O
H2N
NH
NH
N
S
N
N
O
5t
9.5 9.0 8.5 8.0 7.5 7.0 6.5 6.0 5.5 5.0 4.5 4.0 3.5 3.0 2.5 2.0 1.5 1.0 ppm
4.10 0.93 0.93 0.91 0.79 1.00 1.98 4.70 1.93 4.43 4.10 2.35 1.78 6.44 8.24 4.80 2.46 3.17 3.35

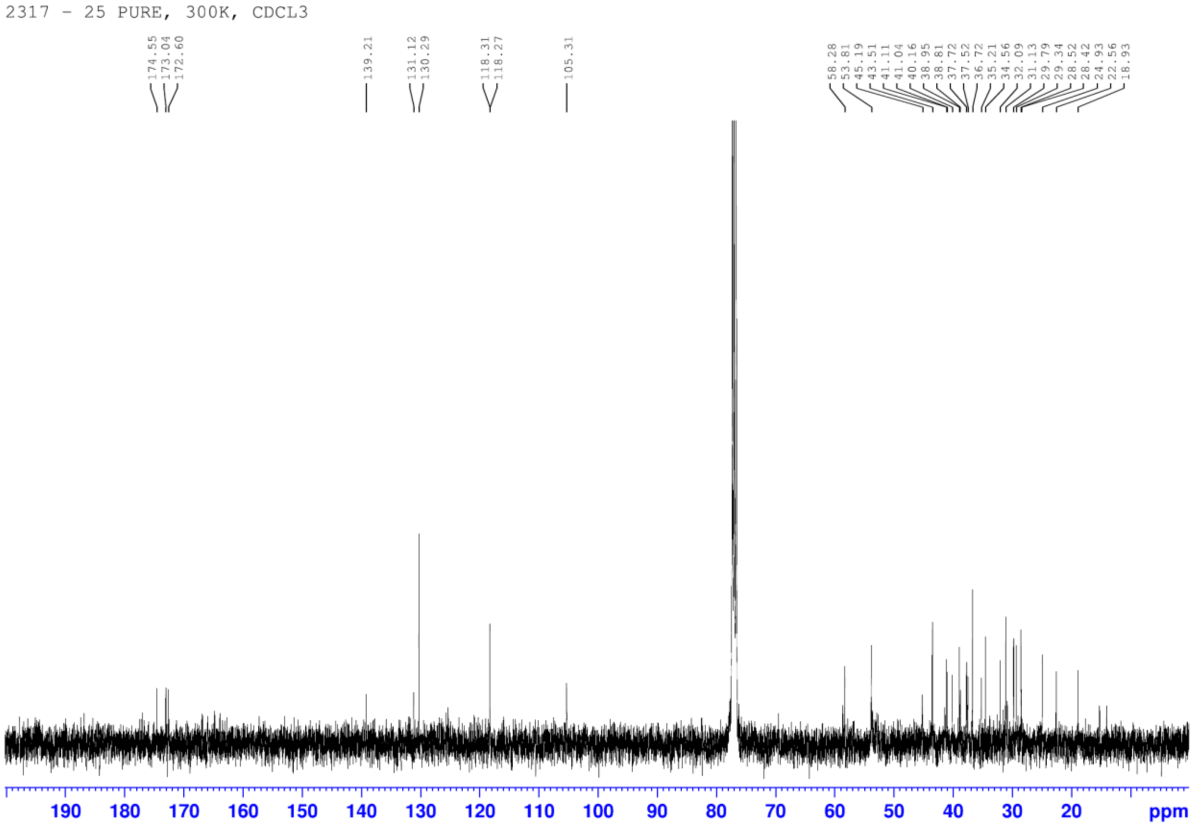
2317 - 25 PURE, 300K, CDCL3
174.55 173.04 172.60 139.21 131.12 130.29 118.31 118.27 105.31
58.28 53.81 45.19 43.51 41.11 41.04 40.16 38.95 38.81 37.72 37.52 36.72 35.21 34.56 32.09 31.13 29.79 29.34 28.52 28.42 24.93 22.56 18.93
190 180 170 160 150 140 130 120 110 100 90 80 70 60 50 40 30 20 ppm

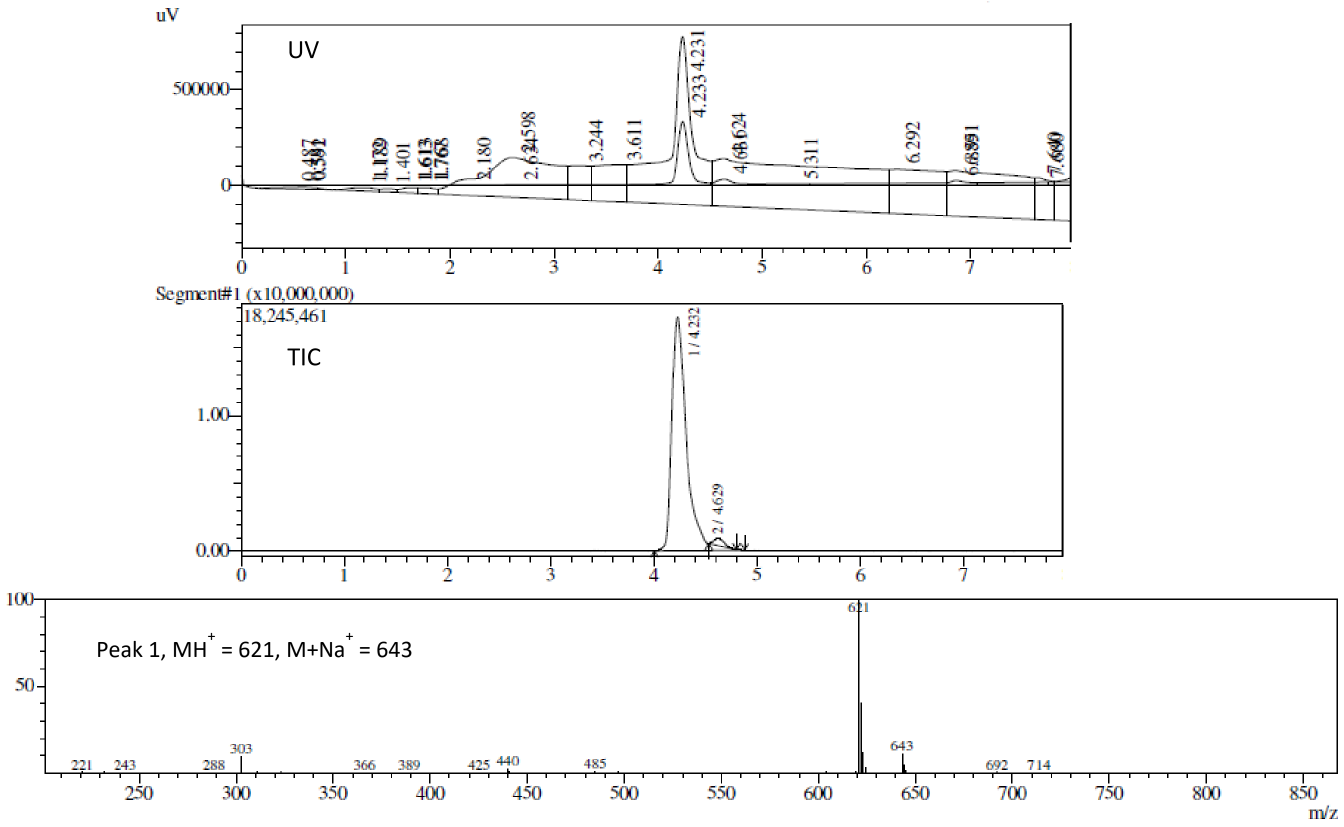
UV
TIC
Peak 1, MH+ = 621, M+Na+ = 643

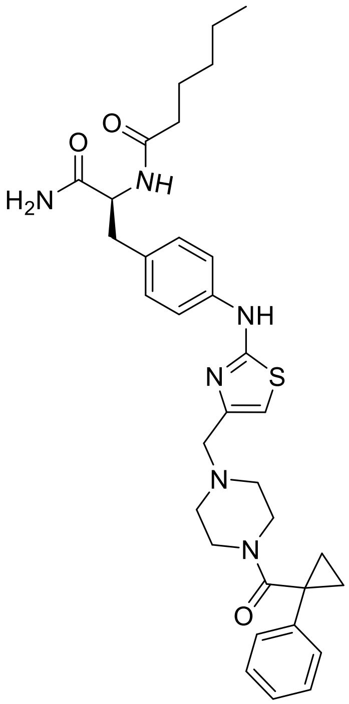

5u
$C_{33}H_{42}N_6O_3S$
Exact Mass: 602.30
Mol. Wt.: 602.79

5u (^{1}H NMR, 400 MHz, $CDCl_3$, 300 K) δ 7.29 (d, J = 6.40 Hz, 2H), 7.22 (d, J = 3.4 Hz, 4H), 7.17 (d, J = 7.4 Hz, 2H), 6.41 (s, 1H), 6.22 (d, J = 8.9 Hz, 1H), 5.94 (s_{broad}, 1H), 5.62 (s_{broad}, 1H), 4.69 (q, J = 8.9 Hz, 1H), 3.71 (s_{broad}, 2H), 3.51 (pent, J = 7.8 Hz, 2H), 3.44 (d, J = 4.3 Hz, 2H), 3.06 (t, J = 5.8 Hz, 2H), 2.50 (s_{broad}, 2H), 2.20 (t, J = 5.3 Hz, 5H), 1.61 (pent, J = 7.3 Hz, 2H), 1.44 – 1.41 (m, 2H), 1.35 – 1.22 (m, 6H), 1.22-1.17 (m, 2H), 0.89 (t, J = 6.6 Hz, 4H)

5u (^{13}C NMR, 100 MHz, $CDCl_3$, 300 K) δ 173.2, 173.0, 170.9, 164.7, 140.7, 139.2, 131.1, 130.3, 128.7, 126.3, 125.3, 118.3, 105.3, 58.2, 53.9, 52.6, 37.6, 36.5, 31.3, 29.4, 25.2, 22.3, 15.2, 13.9

LCMS: $C_{33}H_{42}N_6O_3S$ (M calculated) 602.79, $C_{33}H_{43}N_6O_3S$ (MH^+ found) 603

2317 - 26 PURE, 300K, CDCL3

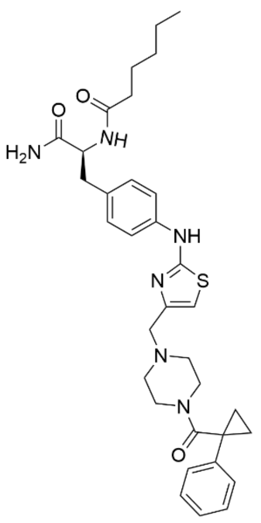

5u

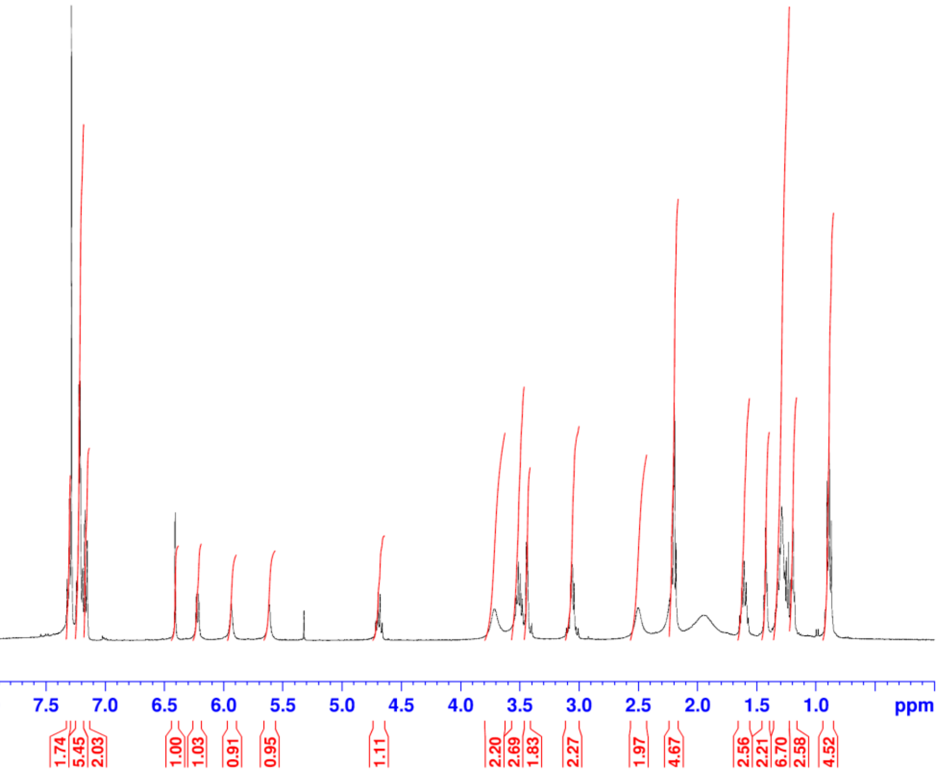

2317 - 26 PURE, 300K, CDCL3

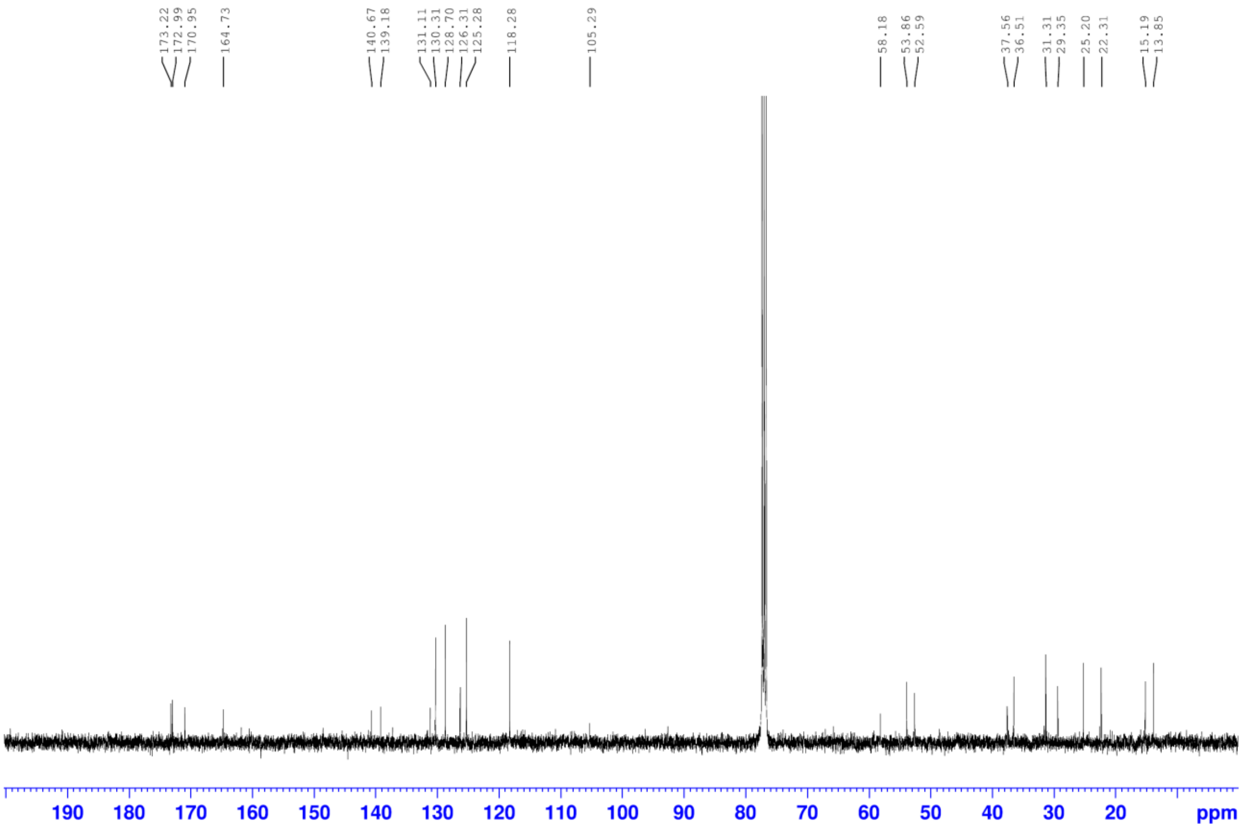

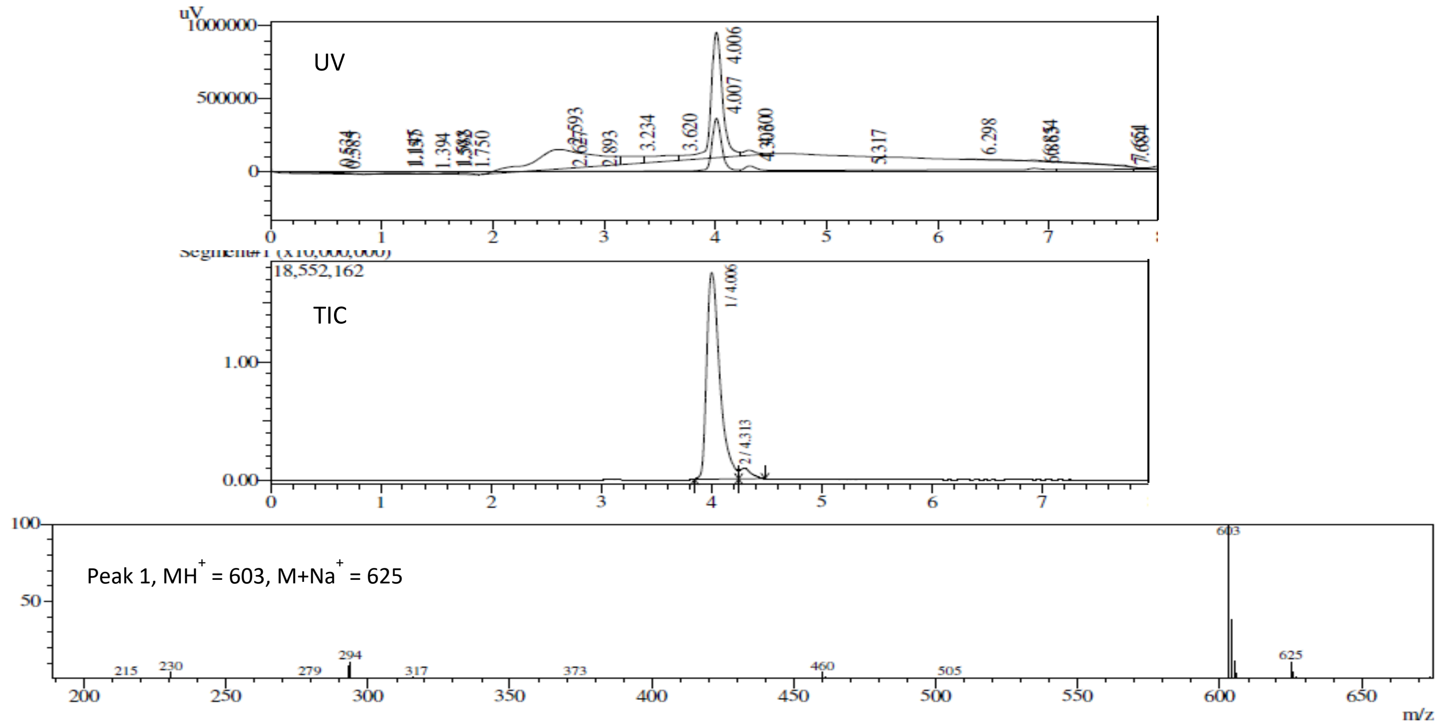

UV
TIC
Peak 1, MH+ = 603, M+Na+ = 625
m/z

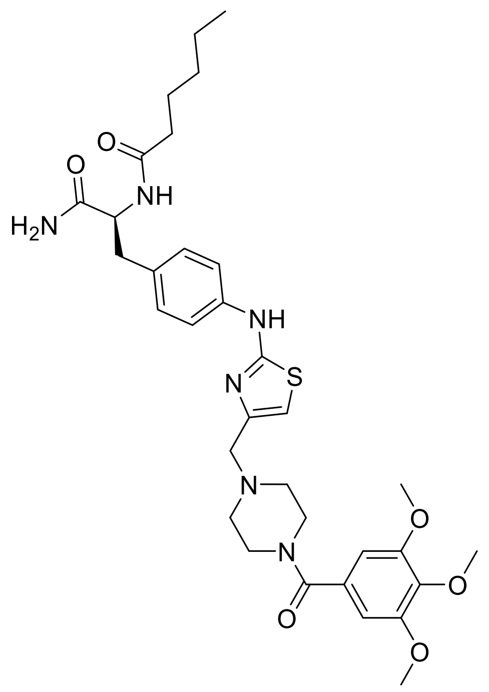

5v
$C_{33}H_{44}N_6O_6S$
Exact Mass: 652.30
Mol. Wt.: 652.80

5v (^{1}H NMR, 400 MHz, $CDCl_3$, 300 K) δ 7.23 (q, J = 7.6 Hz, 4H), 6.64 (s, 2H), 6.46 (s, 1H), 6.22 (d, J = 7.6 Hz, 1H), 5.93 (s_{broad}, 1H), 5.63 (s_{broad}, 1H), 4.69 (q, J = 8.1 Hz, 1H), 3.88 (s, 6H), 3.87 (s, 3H), 3.55 (d, J = 4.5 Hz, 2H), 3.53 – 3.48 (m, 1H), 3.09-3.04 (m, 2H), 2.56 ($s_{v.broad}$, 4H), 2.20 (t, J = 6.7 Hz, 3H), 1.61 (pent, J = 8.9 Hz, 1H), 1.36 – 1.21 (m, 6H), 0.89 (t, J = 6.7 Hz, 4H)

5v (^{13}C NMR, 100 MHz, $CDCl_3$, 300 K) δ 173.2, 173.0, 170.1, 153.3, 139.4, 139.1, 131.2, 131.1, 130.3, 118.3, 104.6, 60.9, 58.2, 56.3, 53.9, 37.5, 36.5, 31.3, 25.2, 22.3, 13.9

LCMS: $C_{33}H_{44}N_6O_6S$ (M calculated) 652.80, $C_{33}H_{45}N_6O_6S$ (MH^+ found) 653

2317 - 28 PURE, 300K, CDCL3

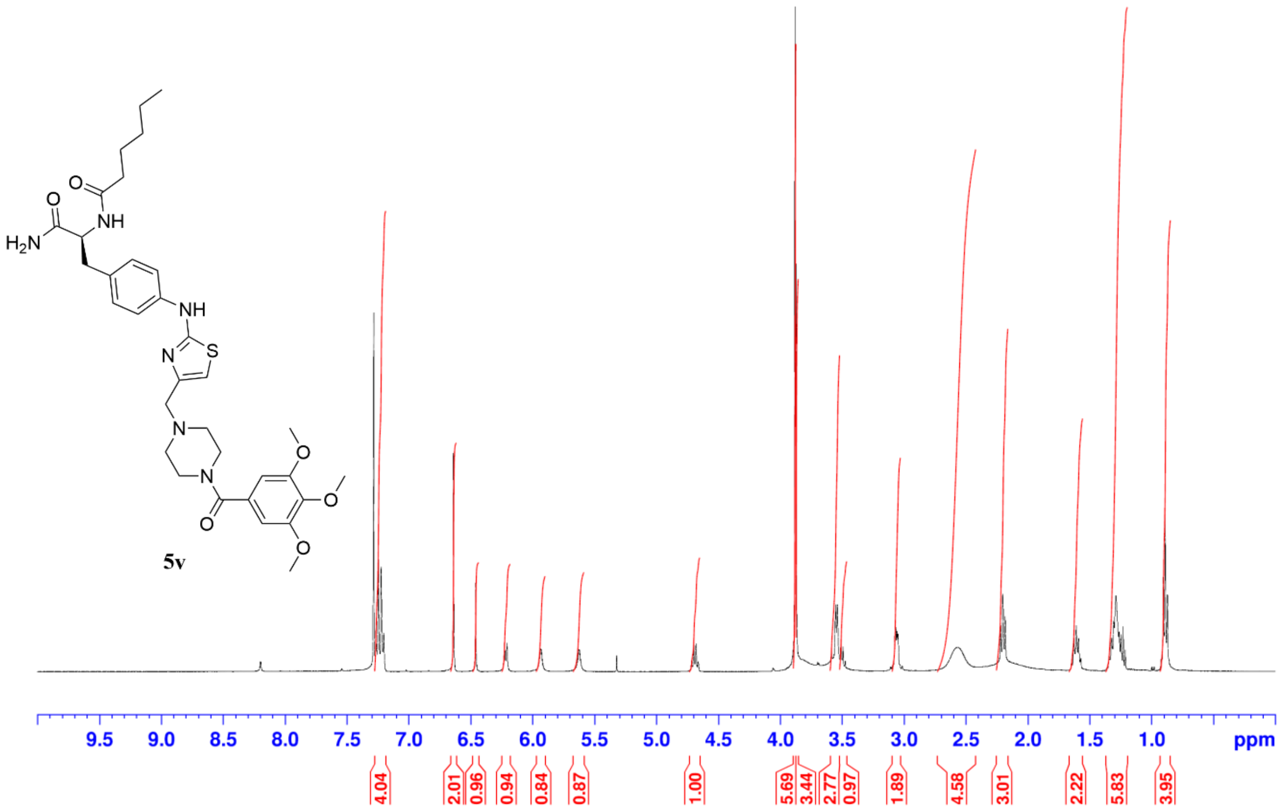

5v

2317 - 28 PURE, 300K, CDCL3

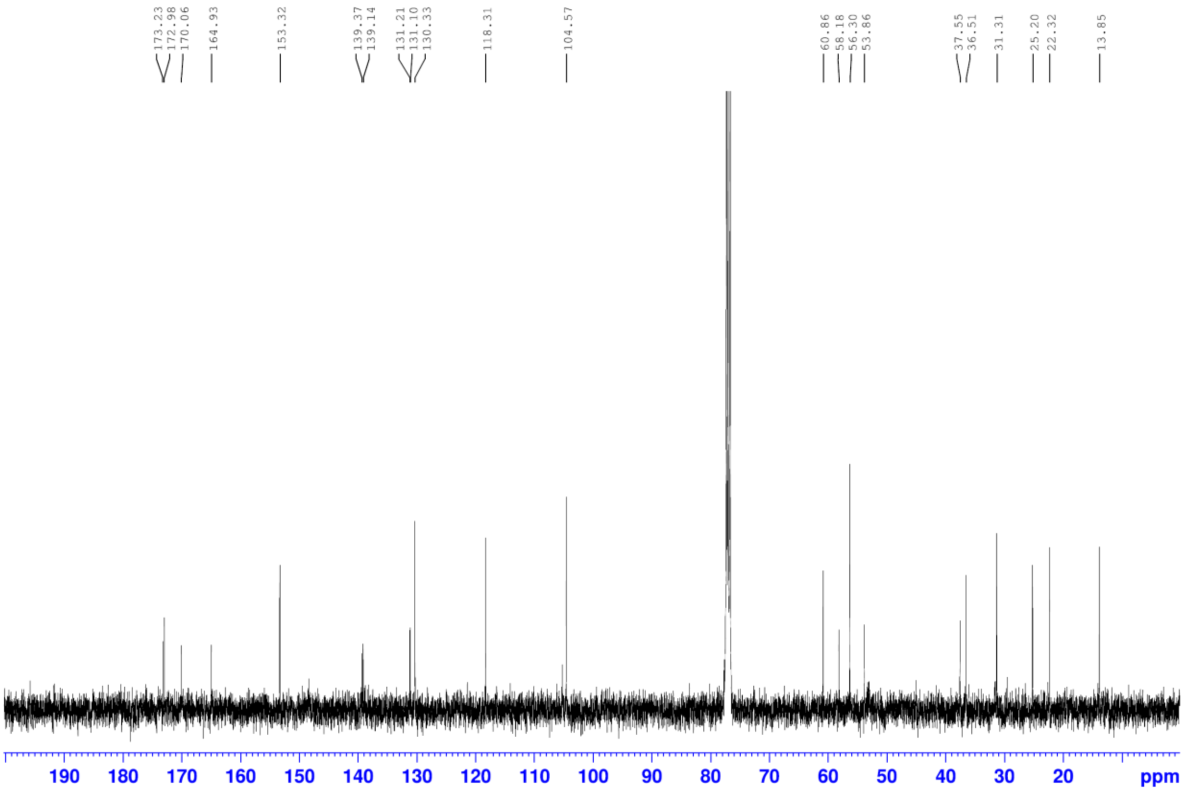

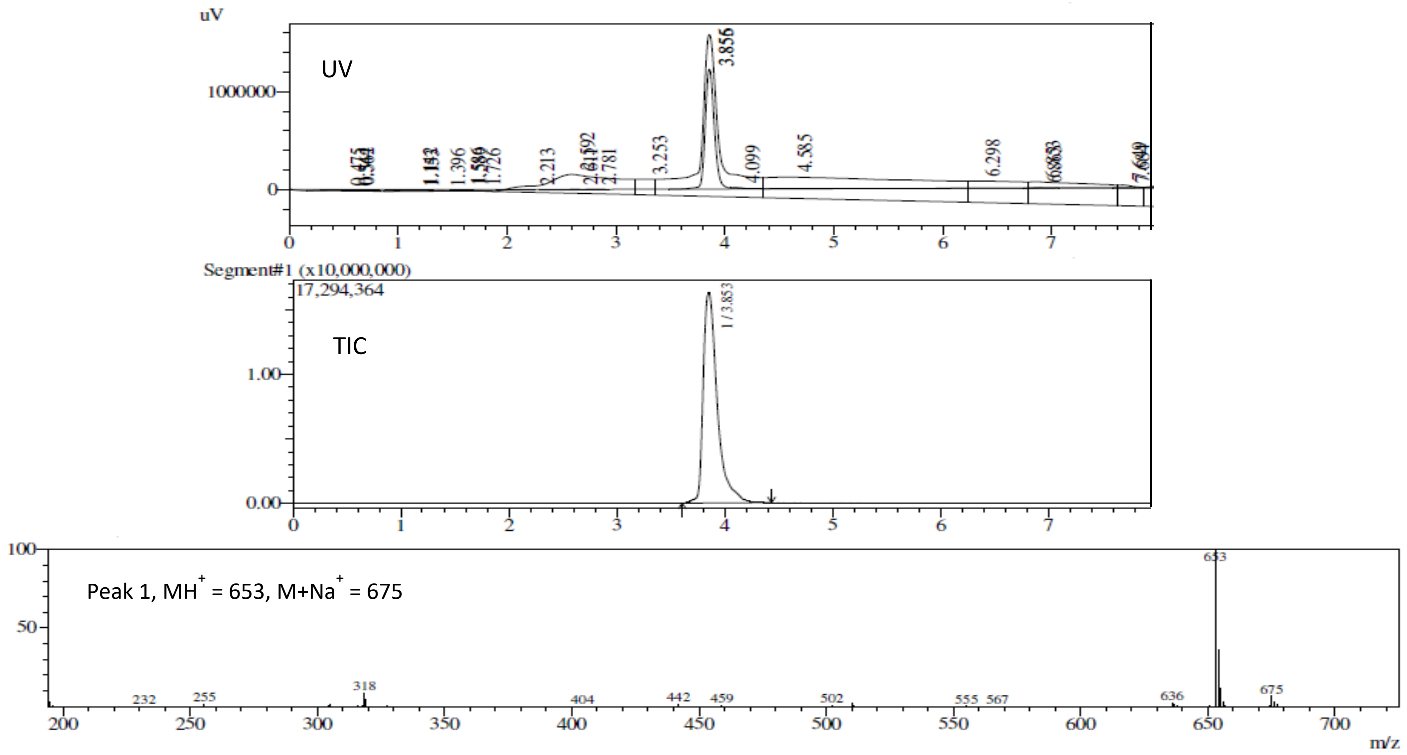
uV
UV
TIC
Segment#1 (x10,000,000)
17,294,364
Peak 1, MH$^+$ = 653, M+Na$^+$ = 675
m/z

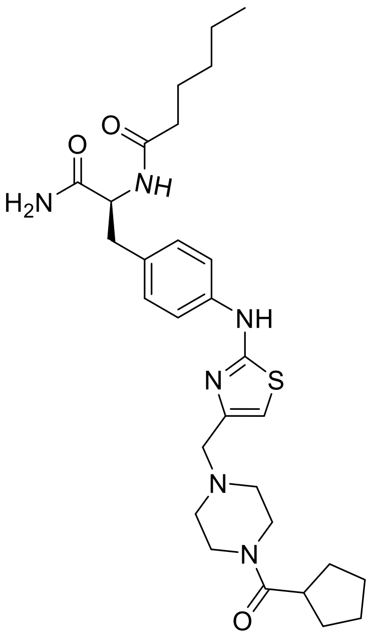

5w

$C_{29}H_{42}N_6O_3S$

Exact Mass: 554.30

Mol. Wt.: 554.75

5w (^{1}**H NMR, 400 MHz, $CDCl_3$, 300 K)** δ 7.27 (d, J = 7.0 Hz, 2H), 7.20 (d, J = 7.7 Hz, 2H), 6.45 (s, 2H), 6.30 (d, J = 9.8 Hz, 1H), 6.02 (s_{broad}, 1H), 5.76 (s_{broad}, 1H), 4.70 (q, J = 7.7 Hz, 1H), 3.76-3.65 (m, 2H), 3.61-3.55 (m, 2H), 3.52 (d, J = 11.5 Hz, 2H), 3.09-3.03 (m, 2H), 2.89 (pent, J = 8.5 Hz, 1H), 2.54 (s_{broad}, 2H), 2.50 ($s_{broad, 2H),}$ 2.21 (t, J = 7.7 Hz, 2H), 2.19 (s, 2H), 1.88-1.72 (7H), 1.66-1.55 (m, 5H), 1.36-1.21 (m, 5H), 0.89 (t, J = 6.6 Hz, 3H)

5w (^{13}**C NMR, 100 MHz, $CDCl_3$, 300 K)** δ 174.5, 173.2, 173.1, 164.7, 148.7, 139.3, 131.0, 130.3, 118.2, 105.3, 58.3, 53.9, 53.5, 52.9, 45.3, 41.6, 41.0, 37.6, 36.5, 31.3, 30.8, 30.1, 26.0, 25.2, 22.3, 13.9

LCMS: $C_{29}H_{42}N_6O_3S$ (M calculated) 554.75, $C_{29}H_{43}N_6O_3S$ (MH^+ found) 555

2317 - 29 PURE, 300K, CDCL3

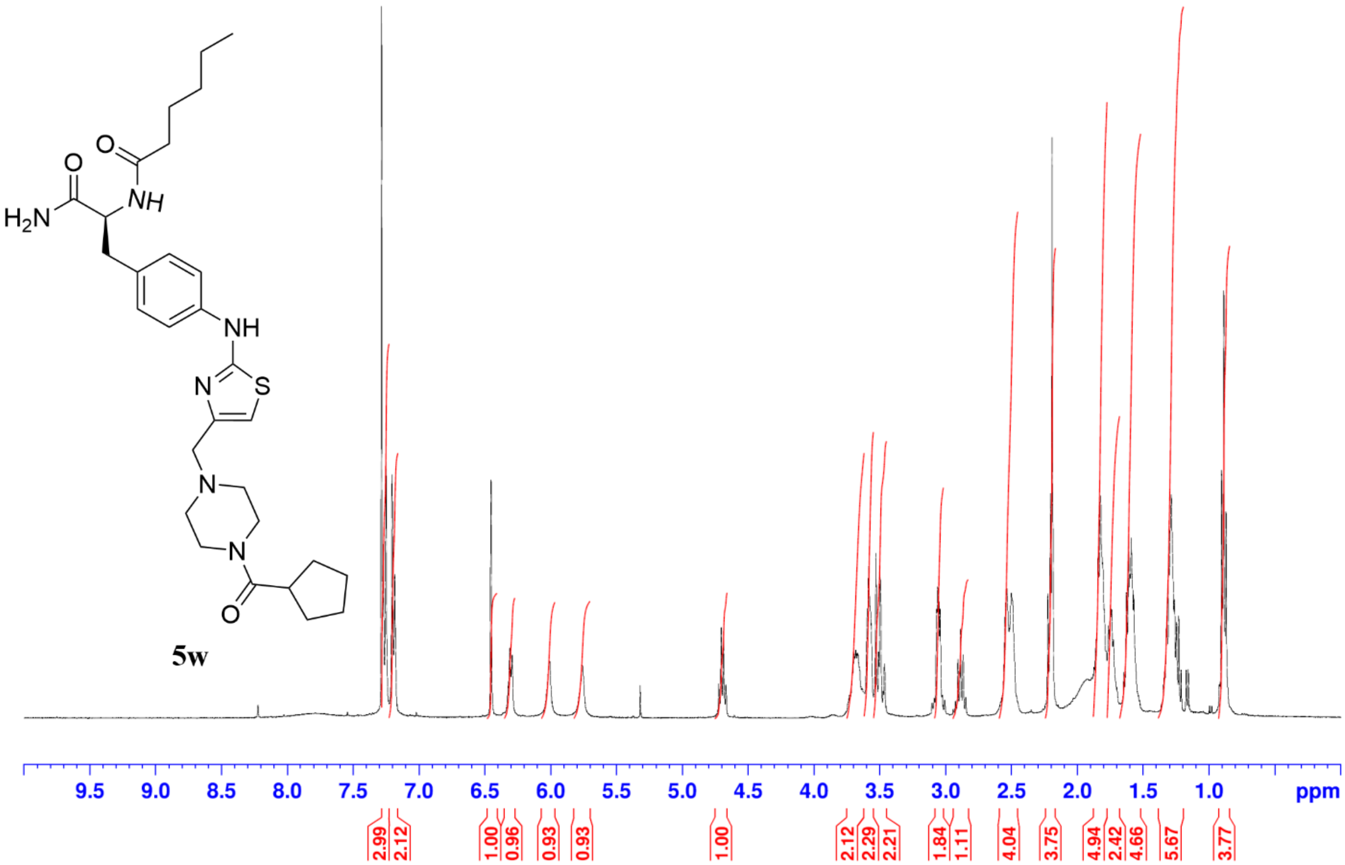

5w

2317 - 29 PURE, 300K, CDCL3

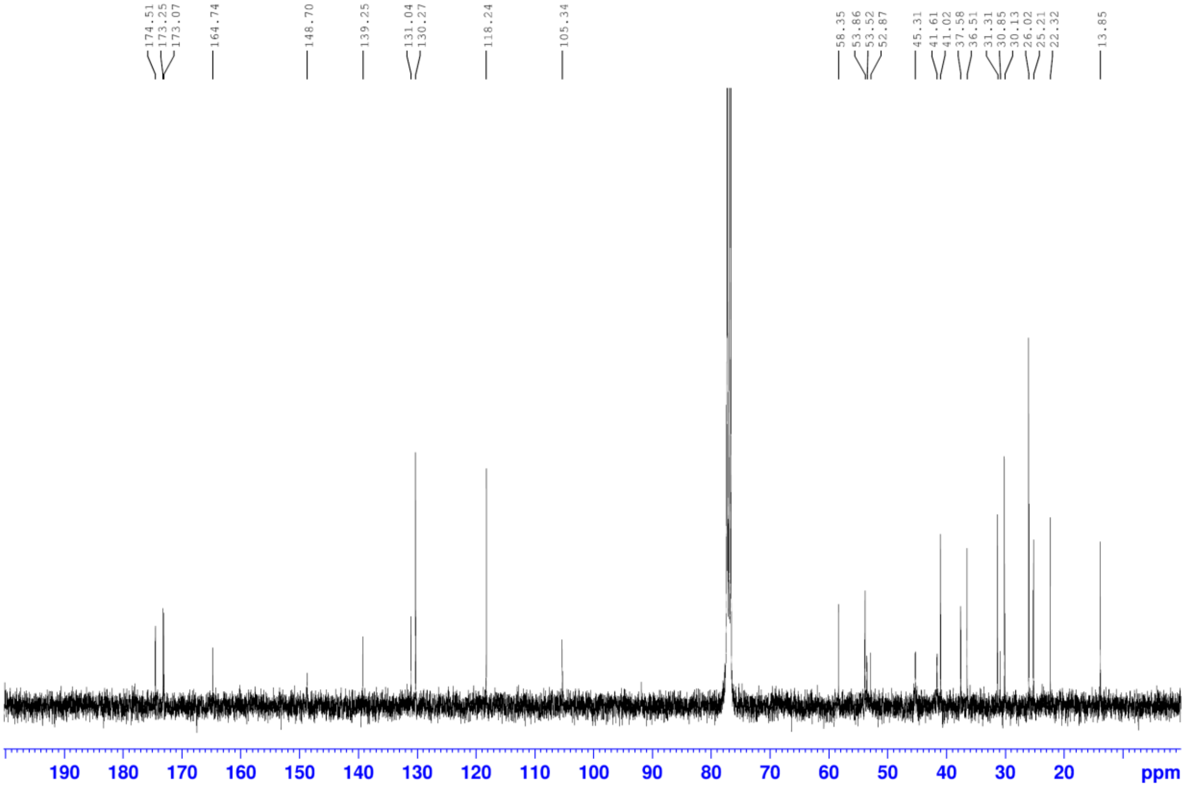

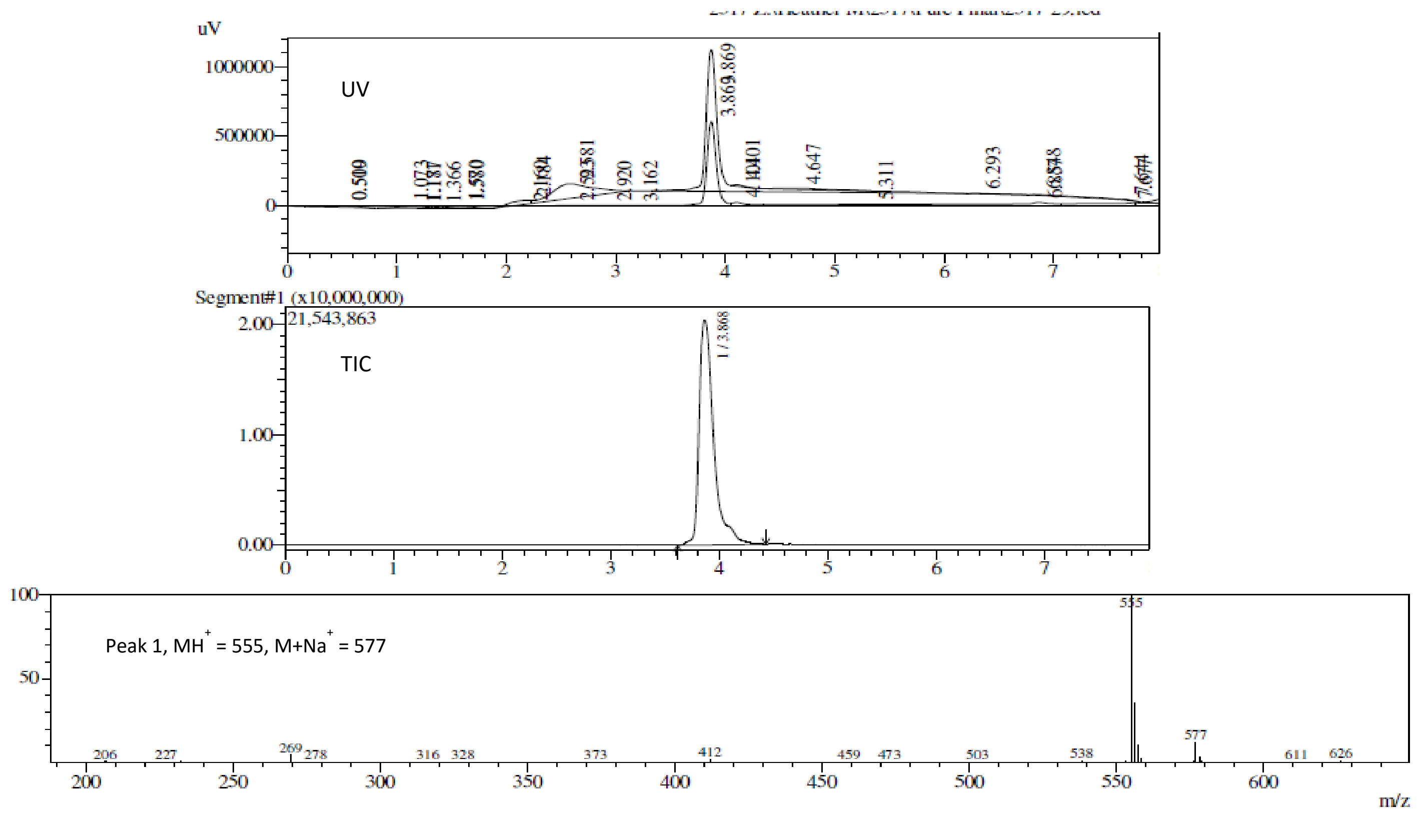
uV
UV
3.869
Segment#1 (x10,000,000)
21,543,863
TIC
1 / 3.868
Peak 1, MH+ = 555, M+Na+ = 577
555
577
m/z

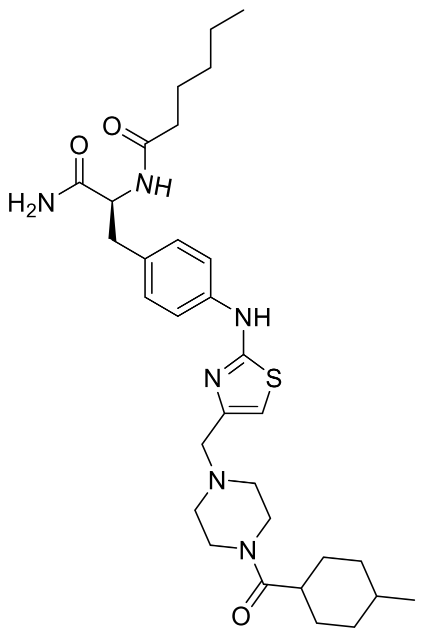

5x
$C_{31}H_{46}N_6O_3S$
Exact Mass: 582.34
Mol. Wt.: 582.80

5x (^{1}H NMR, 400 MHz, $CDCl_3$, 300 K) δ 7.26 (d, J = 8.0 Hz, 2H), 7.20 (d, J = 8.7 Hz, 2H), 6.45 (s, 1H), 6.27 (d, J = 8.0 Hz, 1H), 5.97 (s_{broad}, 1H), 5.71 (s_{broad}, 1H), 4.69 (q, J = 9.8 Hz, 1H), 3.67 (s_{vbroad}, 2H), 3.59 – 3.46 (m, 4H), 3.06 (t, J = 5.4 Hz, 2H), 2.55 (s_{broad}, 2H), 2.50 (s_{broad}, 2H), 2.21 (d, J = 7.3 Hz, 1H), 2.19 (s, 3H), 1.90 ($S_{v.broad}$, 2H), 1.85-1.72 (m, 5H), 1.65-1.47 (m, 7H), 1.34-1.23 (m, 5H), 0.99 (d, J = 7.9 Hz, 3H), 0.89 (t, J = 7.1 Hz, 3H)

5x (^{13}C NMR, 100 MHz, $CDCl_3$, 300 K) δ 174.5, 173.2, 173.0, 164.7, 148.8, 139.2, 131.1, 130.3, 118.2, 105.3, 58.4, 53.9, 40.2, 38.8, 37.6, 36.5, 34.6, 32.1, 30.9, 29.3, 28.4, 25.2, 24.9, 22.6, 22.3, 18.9, 13.9

LCMS: $C_{31}H_{46}N_6O_3S$ (M calculated) 582.80, $C_{31}H_{47}N_6O_3S$ (MH^+ found) 583

2317 - 30 PURE, 300K, CDCL3

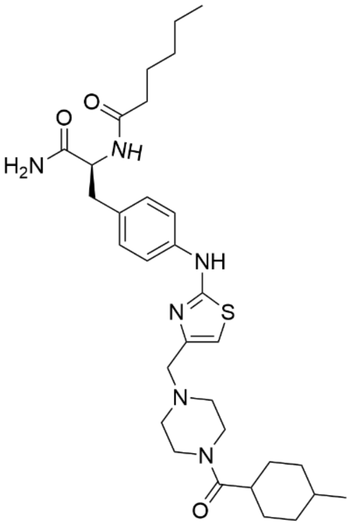

5x

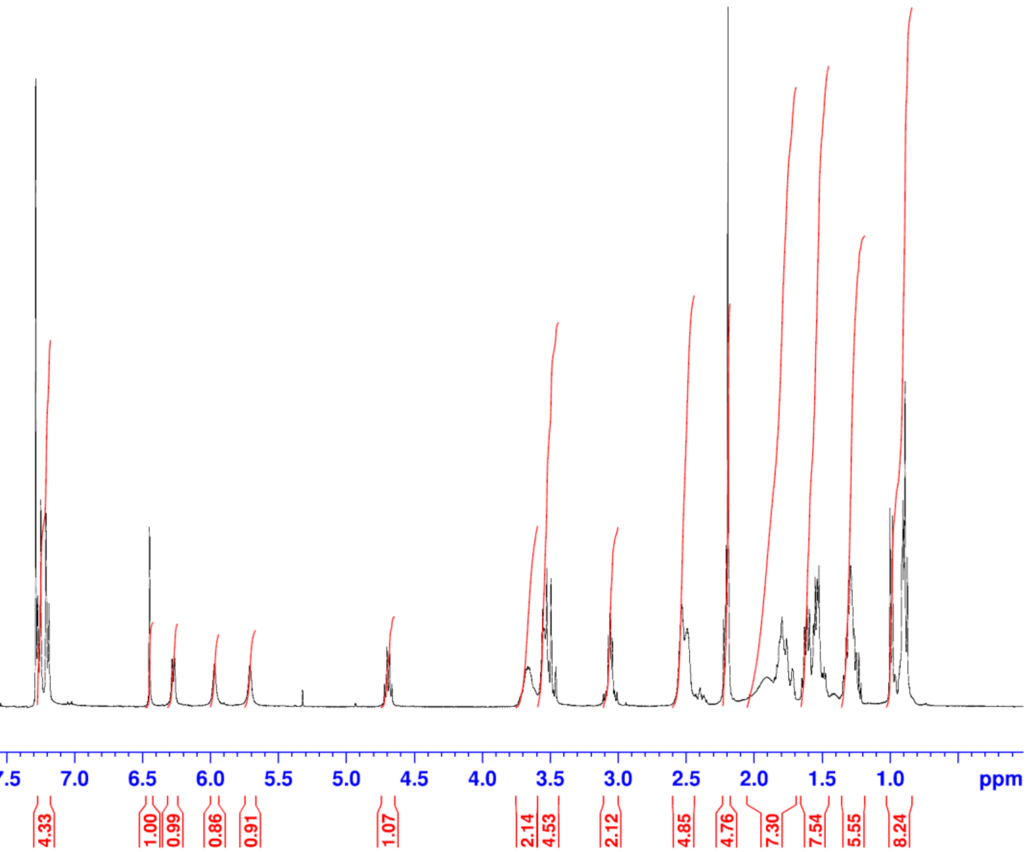

2317 - 30 PURE, 300K, CDCL3

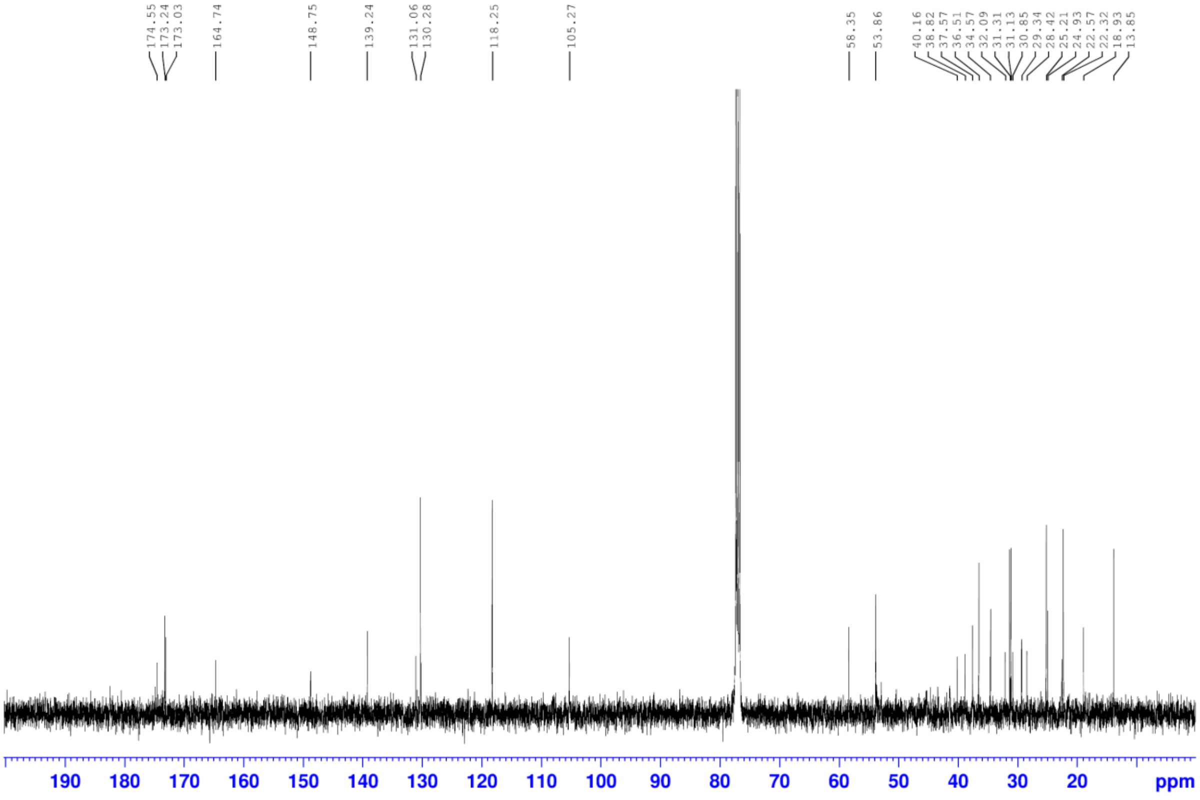

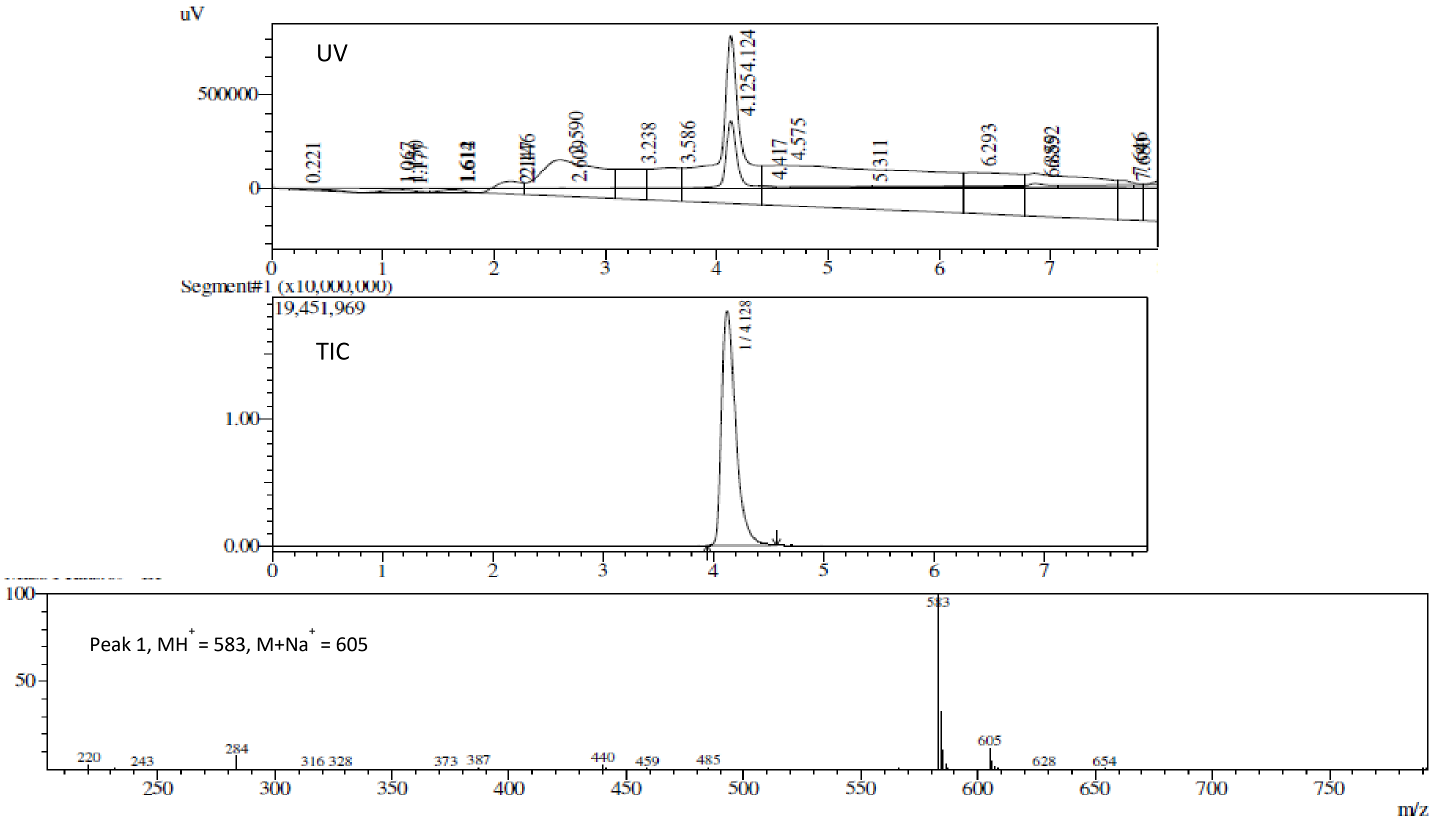
uV
UV
500000
0
4.1254.124
3.586
3.238
4.575
6.293
5.311
0.221
Segment#1 (x10,000,000)
19,451,969
TIC
1.00
0.00
1/4.128
100
50
Peak 1, MH+ = 583, M+Na+ = 605
583
605
220
243
284
316 328
373 387
440
459
485
628
654
m/z

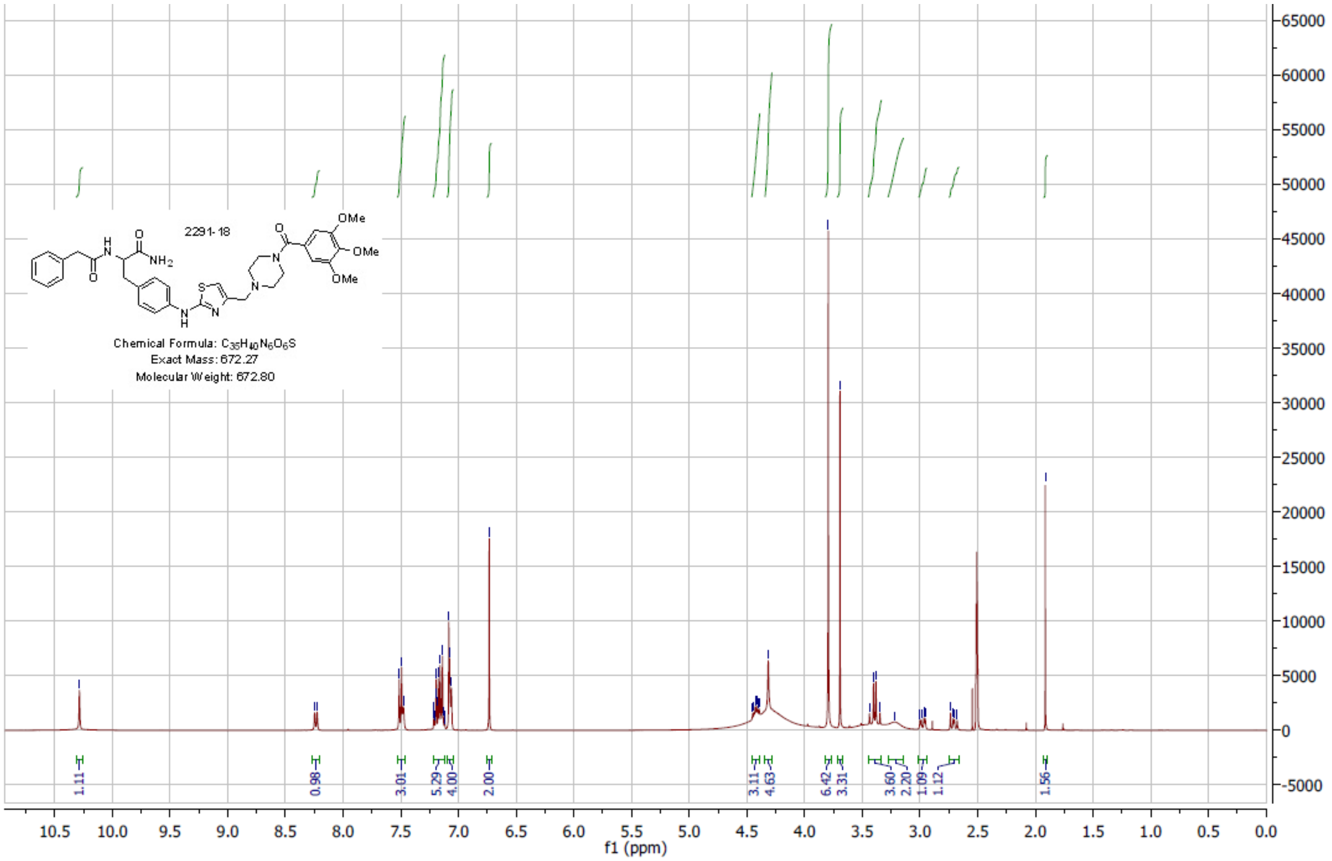

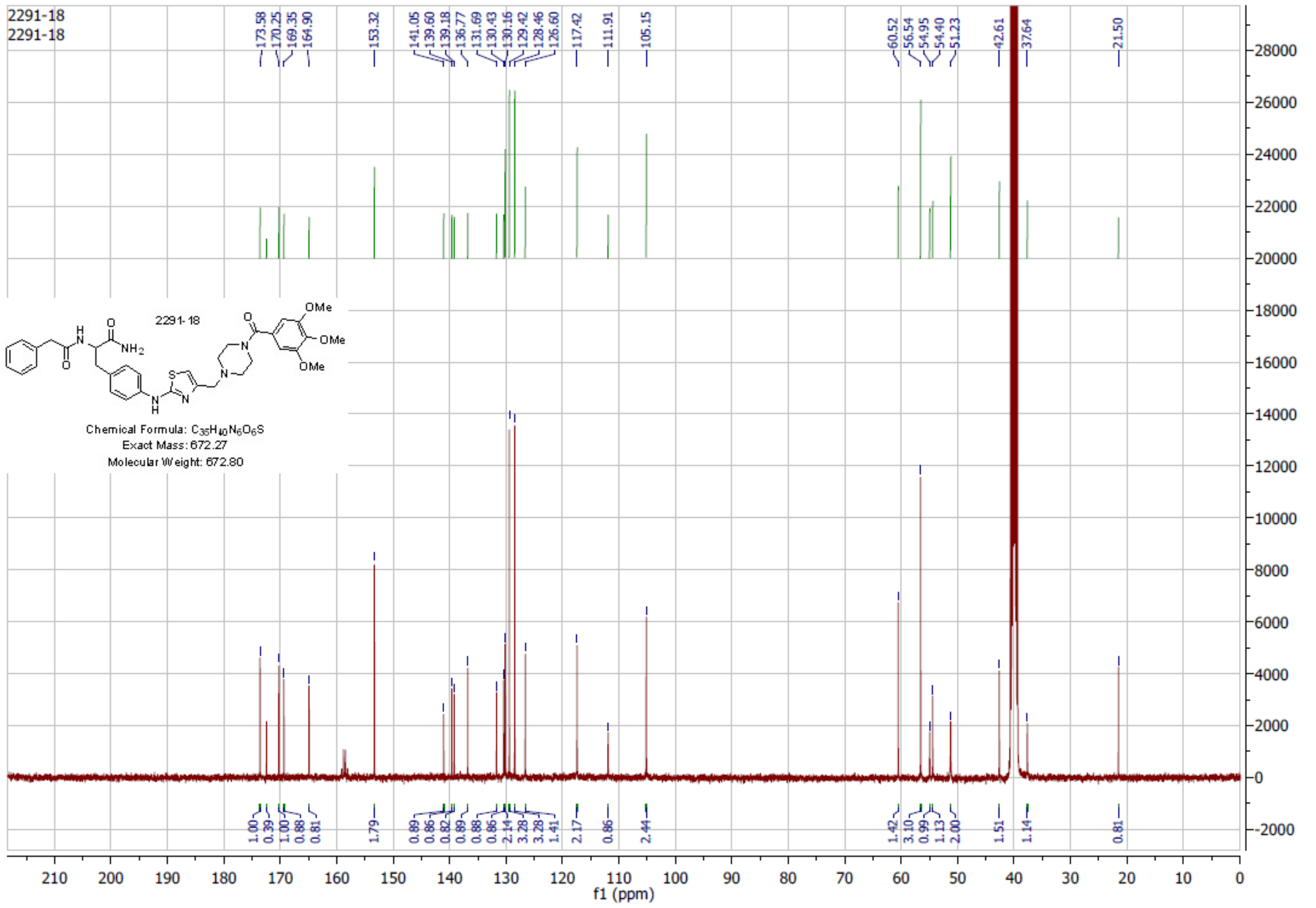

Compound ID Library 2291	Structure	Dd2 EC50 (μM) n=3
2291-61	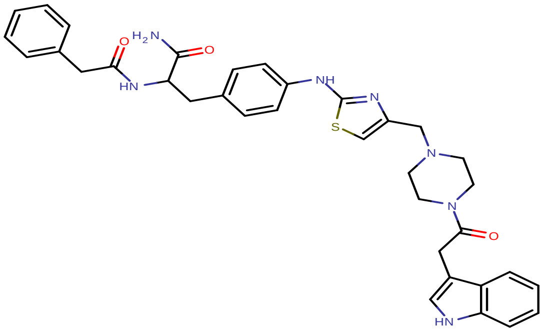	0.102 ± 0.01
2291-18	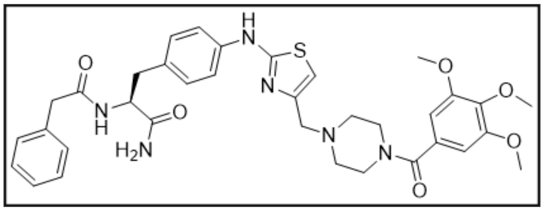	0.220 ± 0.02
2291-135	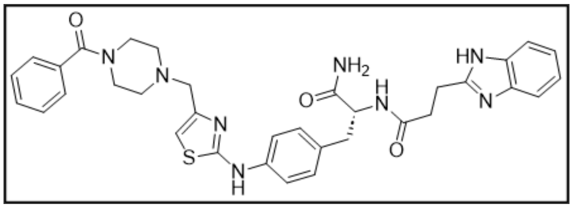	0.190 ± 0.01
2291-134	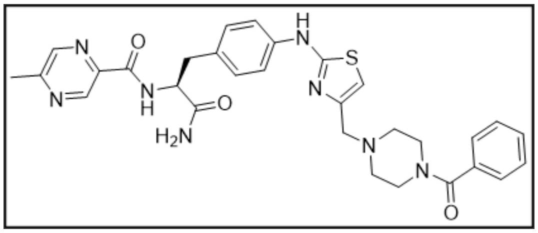	0.200 ± 0.01

SB1-1	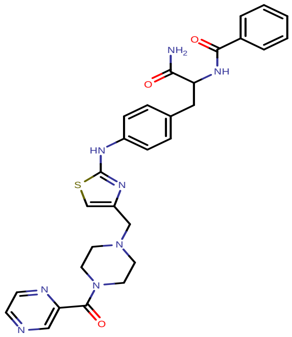	**0.836 ± 0.11**
SB1-2	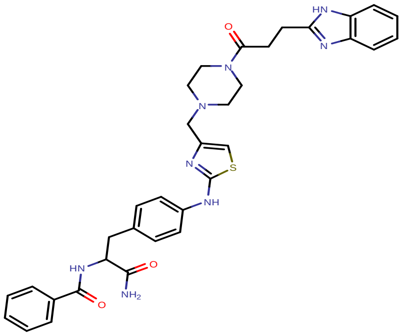	**0.520 ± 0.08**
SB1-3	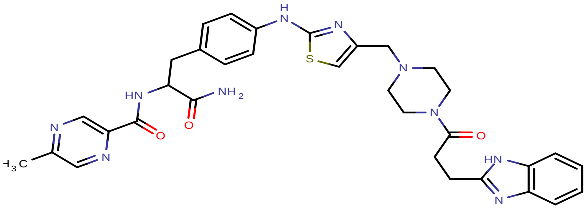	**>2.5**
SB1-4	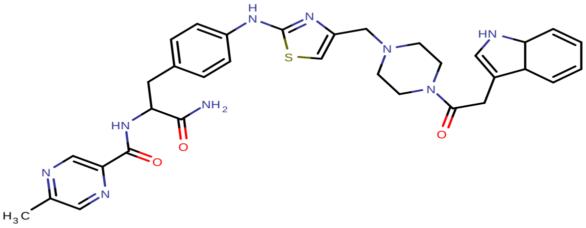	**0.104 ± 0.03**

SB1-5	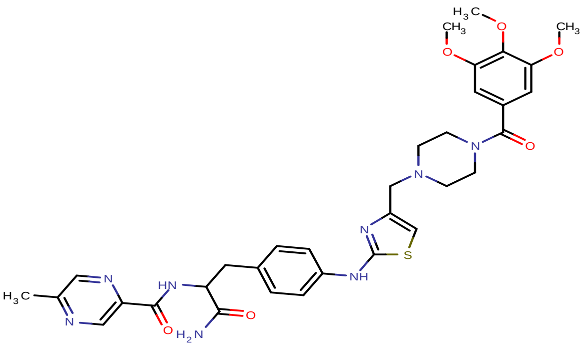	**1.02 ± 0.3**
SB1-8	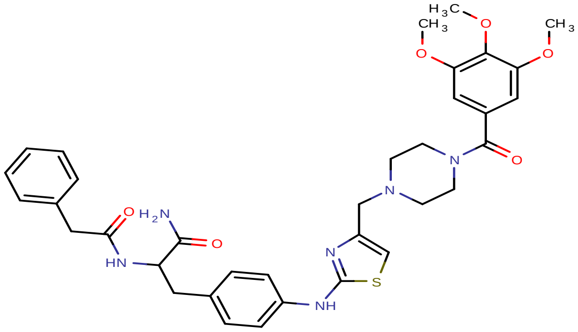	**0.698 ± 0.03**
SB1-9	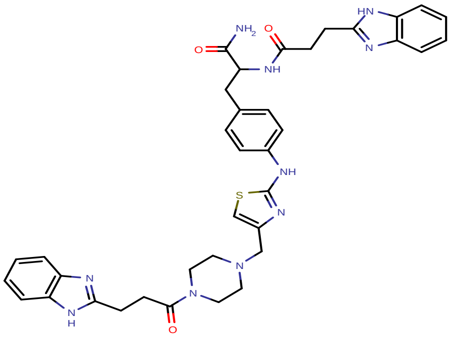	**0.132 ± 0.03**
SB1-10	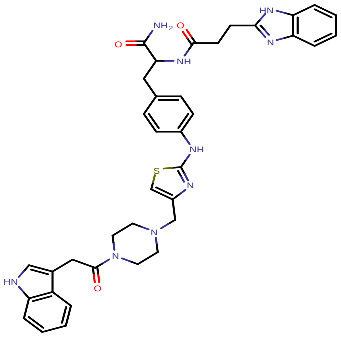	**0.317 ± 0.08**

SB1-11	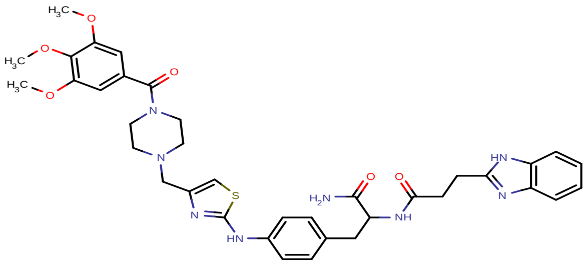	**0.381 ± 0.04**
SB1-12	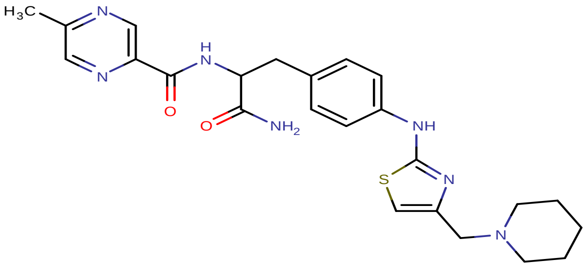	**0.562 ± 0.06**
SB1-13	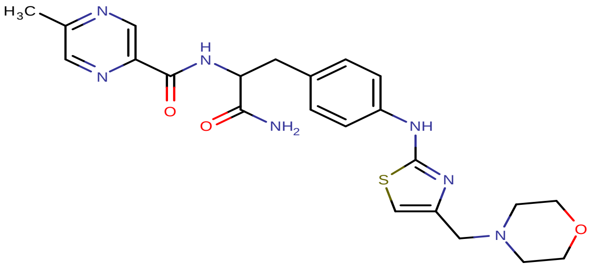	**0.250 ± 0.08**
SB1-14	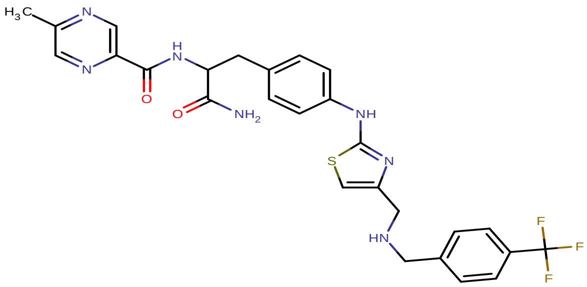	**0.682 ± 0.15**

SB1-15	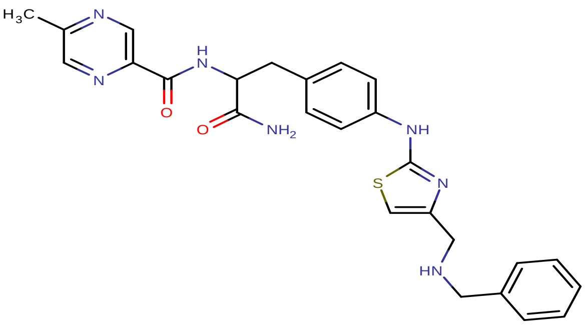	**0.336 ± 0.07**
SB1-16	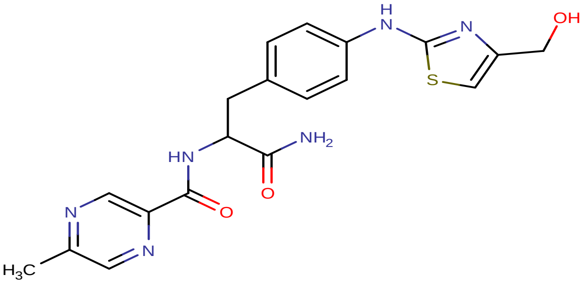	**0.732 ± 0.09**
SB1-19	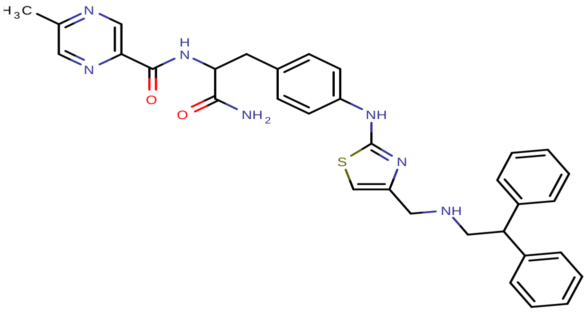	**1.210 ± 0.11**
SB1-20	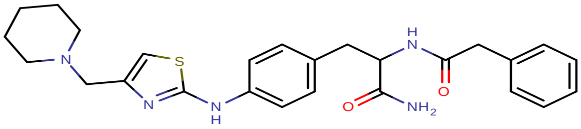	**0.662 ± 0.12**

SB1-21	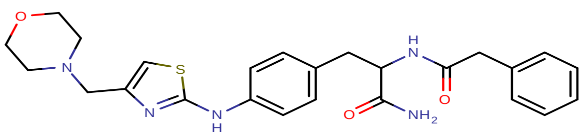	**0.551 ± 0.07**
SB1-22	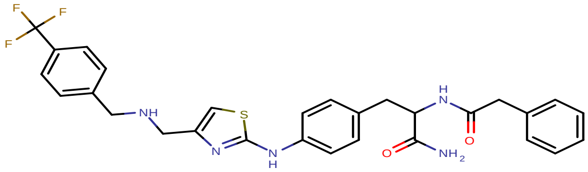	**0.759 ± 0.10**
SB1-23	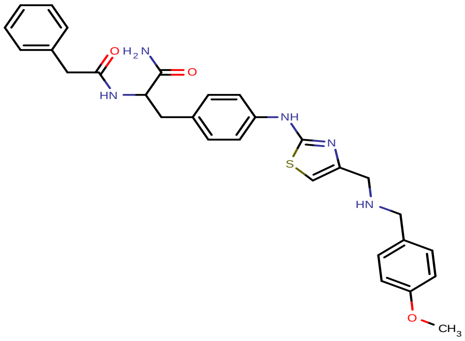	**0.489 ± 0.07**
SB1-25	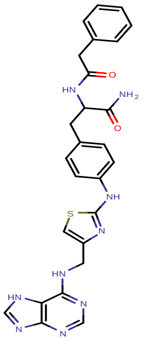	**0.297 ± 0.05**

SB1-27	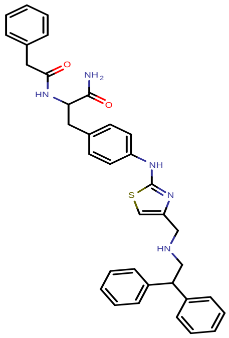	**0.827 ± 0.05**
SB1-29	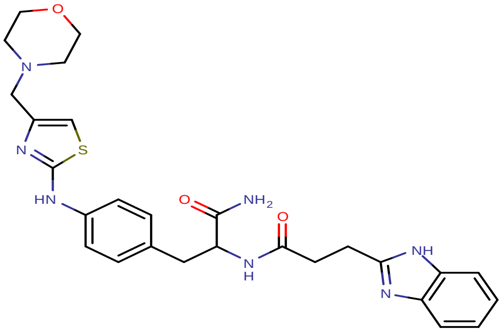	**0.460 ± 0.05**
SB1-32	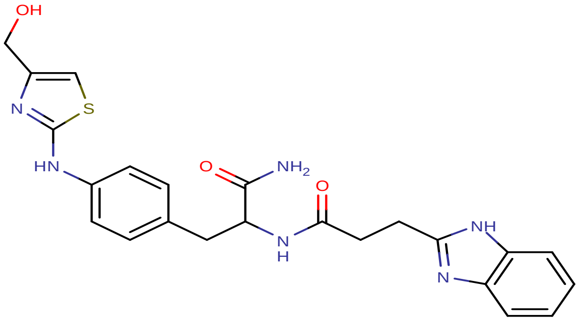	**0.807 ± 0.07**
SB1-37	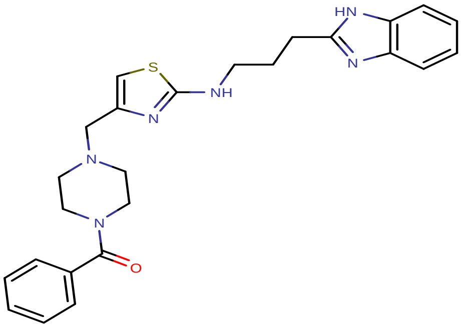	**0.349 ± 0.05**

SB1-41	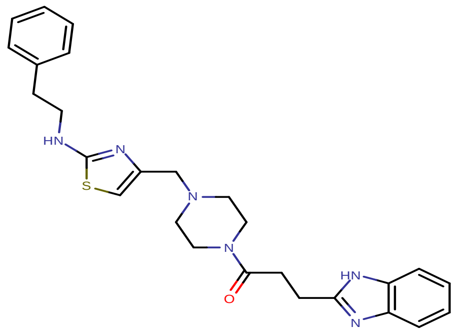	**0.743 ± 0.09**
SB1-42	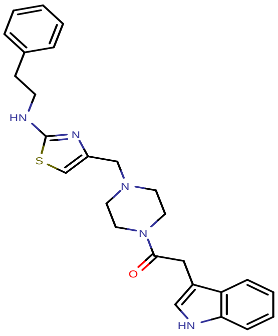	**0.487 ± 0.04**
SB1-43	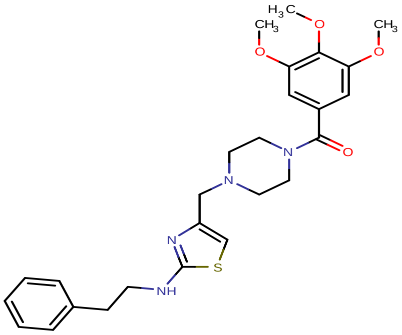	**0.680 ± 0.07**
SB1-44	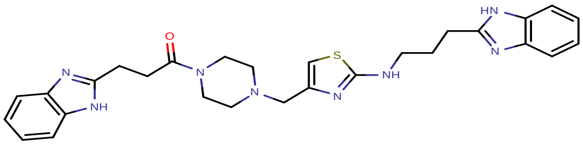	**0.746 ± 0.09**

SB1-45	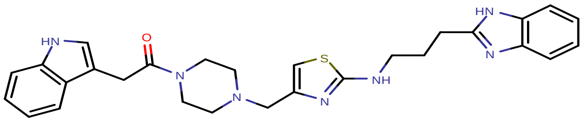	**1.103 ± 0.17**
SB1-46	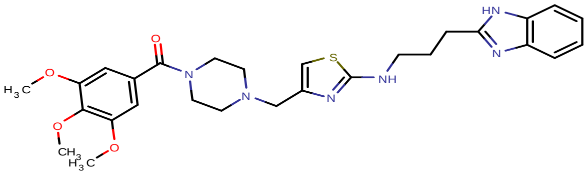	**0.310 ± 0.09**
SB1-49	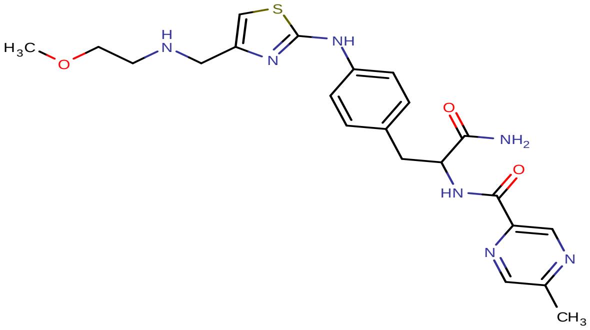	**0.747 ± 0.07**
SB1-50	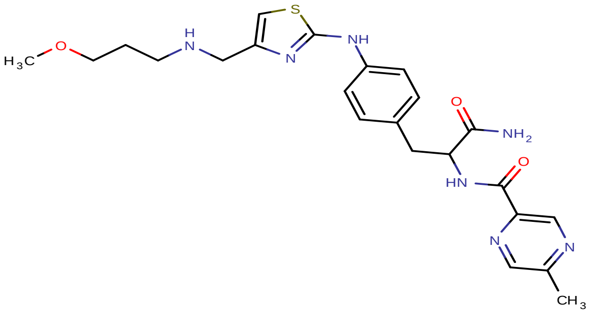	**0.748 ± 0.10**

SB1-51	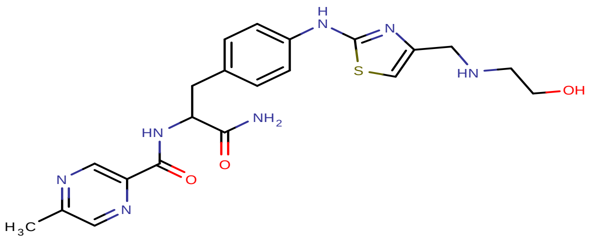	**>2.5**
SB1-52	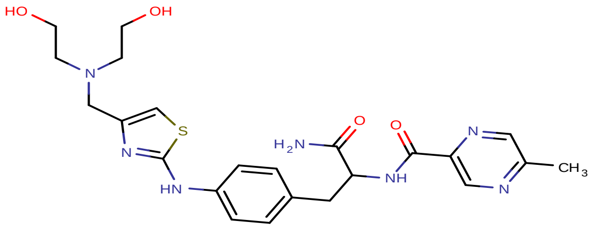	**0.499 ± 0.06**
SB1-53	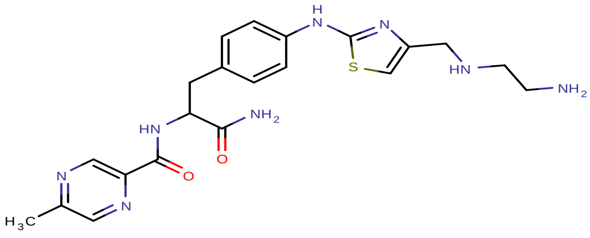	**0.684 ± 0.04**
SB1-54	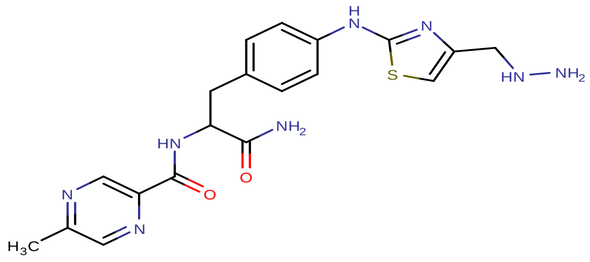	**0.416 ± 0.04**

SB1-55	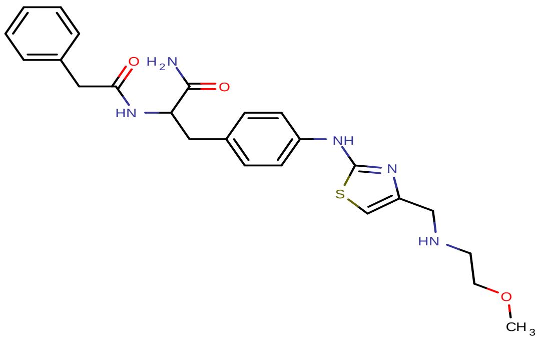	**0.555 ± 0.06**
SB1-56	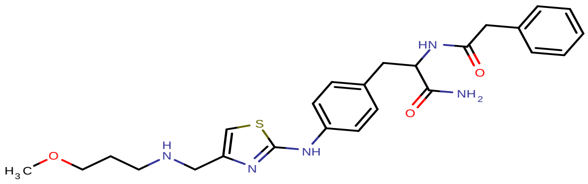	**0.513 ± 0.05**
SB1-57	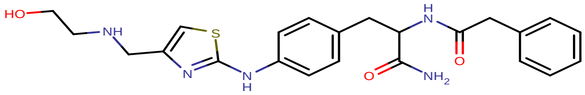	**0.985 ± 0.15**
SB1-58	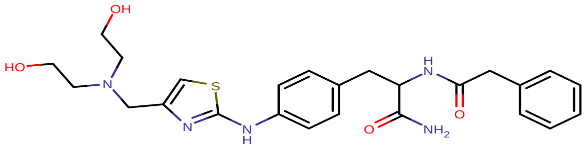	**0.655 ± 0.03**

SB1-59	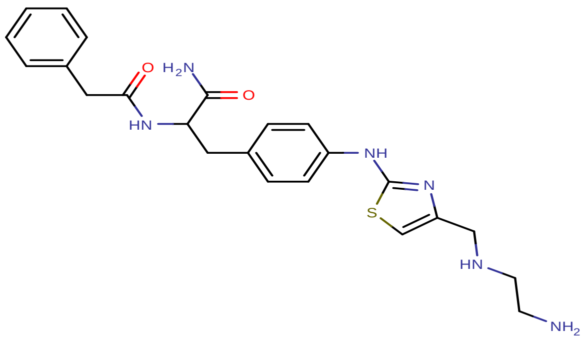	**0.531 ± 0.07**
SB1-60	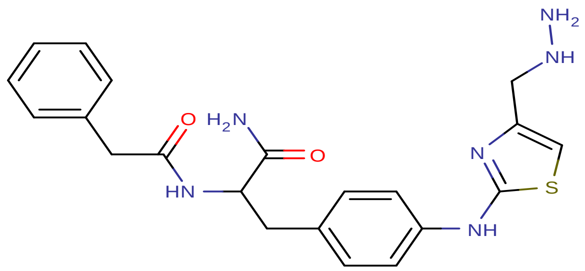	**0.524 ± 0.06**
SB1-64	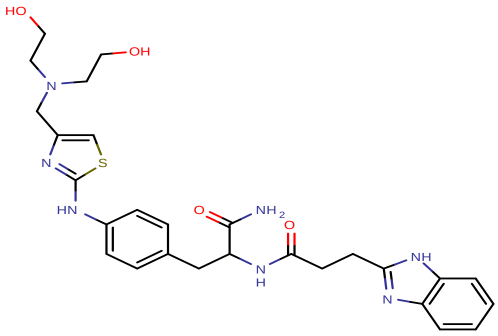	**0.267 ± 0.05**
